# KAT7/HBO1/MYST2 Regulates CENP-A Chromatin Assembly by Antagonizing Suv39h1-Mediated Centromere Inactivation

**DOI:** 10.1016/j.devcel.2016.05.006

**Published:** 2016-06-06

**Authors:** Jun-ichirou Ohzeki, Nobuaki Shono, Koichiro Otake, Nuno M.C. Martins, Kazuto Kugou, Hiroshi Kimura, Takahiro Nagase, Vladimir Larionov, William C. Earnshaw, Hiroshi Masumoto

**Affiliations:** 1 Laboratory of Cell Engineering, Department of Frontier Research, Kazusa DNA Research Institute, 2-6-7 Kazusa-Kamatari, Kisarazu 292-0818, Japan; 2 Wellcome Trust Centre for Cell Biology, University of Edinburgh, Edinburgh EH9 3BF, UK; 3 Department of Biological Sciences, Graduate School of Bioscience and Biotechnology, Tokyo Institute of Technology, Yokohama 226-8501, Japan; 4 Public Relations Team, Kazusa DNA Research Institute, Kisarazu 292-0818, Japan; 5 Genome Structure and Function Group, Developmental Therapeutics Branch, Center for Cancer Research, National Cancer Institute, National Institutes of Health, Bethesda, MD 20892, USA

## Abstract

Centromere chromatin containing histone H3 variant CENP-A is required for accurate chromosome segregation as a foundation for kinetochore assembly. Human centromere chromatin assembles on a part of the long α-satellite (alphoid) DNA array, where it is flanked by pericentric heterochromatin. Heterochromatin spreads into adjacent chromatin and represses gene expression, and it can antagonize centromere function or CENP-A assembly. Here, we demonstrate an interaction between CENP-A assembly factor M18BP1 and acetyltransferase KAT7/HBO1/MYST2. Knocking out KAT7 in HeLa cells reduced centromeric CENP-A assembly. Mitotic chromosome misalignment and micronuclei formation increased in the knockout cells and were enhanced when the histone H3-K9 trimethylase Suv39h1 was overproduced. Tethering KAT7 to an ectopic alphoid DNA integration site removed heterochromatic H3K9me3 modification and was sufficient to stimulate new CENP-A or histone H3.3 assembly. Thus, KAT7-containing acetyltransferases associating with the Mis18 complex provides competence for histone turnover/exchange activity on alphoid DNA and prevents Suv39h1-mediated heterochromatin invasion into centromeres.

## Highlights

• The histone acetyltransferase KAT7 positively regulates centromeric CENP-A assembly
• Human Mis18 complex is a scaffold for assembly of KAT7 and HJURP, a CENP-A chaperone
• KAT7 or RSF1 stimulates histone turnover/exchange on alphoid DNA
• KAT7 antagonizes H3K9-trimethylase Suv39h1-mediated centromere inactivation

## Graphical Abstract

**Figure:**
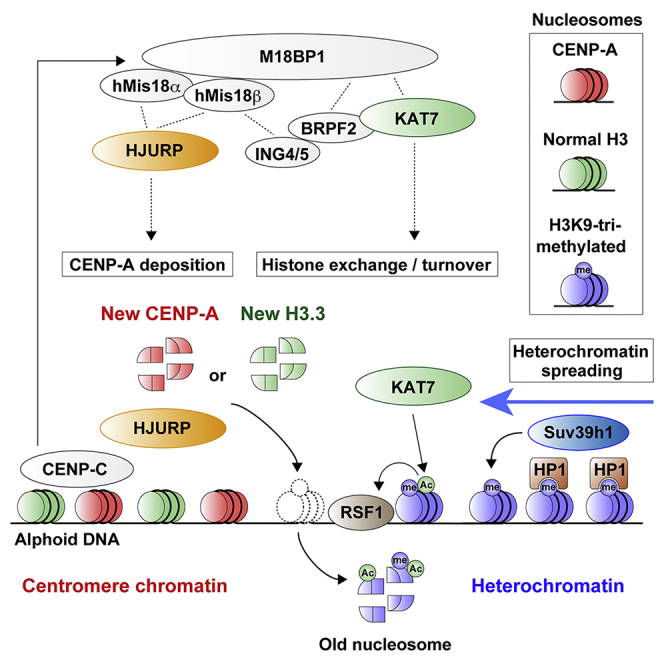

## Teaser

Human centromeres contain histone H3 variant CENP-A and are flanked by pericentric heterochromatin. Spreading of heterochromatin into the centromere can impede centromere function. Ohzeki et al. demonstrate that the KAT7 histone acetyltransferase promotes CENP-A assembly and preserves centromere function by preventing H3K9-trimethylase Suv39h1-mediated heterochromatin invasion via a histone turnover mechanism.

## Introduction

The centromere is a specialized chromatin locus of eukaryotic chromosome containing the conserved specific histone H3 variant CENP-A (Earnshaw and Rothfield, 1985). During mitosis, kinetochore proteins assembled on centromeric chromatin direct accurate chromosomal segregation processes by interacting with microtubules (Cleveland et al., 2003, Allshire and Karpen, 2008, Fukagawa and Earnshaw, 2014). CENP-A is an essential component for the maintenance of centromere chromatin identity, providing a mark on which other centromere-specific proteins (i.e., the constitutive centromere associated network, CCAN) assemble throughout the cell cycle (Cheeseman and Desai, 2008). Sixteen CCAN proteins, including CENP-C and CENP-H, are currently known to assemble on nucleosomes containing CENP-A in vertebrates (Carroll et al., 2010, Black and Cleveland, 2011, Basilico et al., 2014).

In humans, CENP-A chromatin localizes to a portion of the α-satellite (alphoid) locus, a pleiomorphic, repetitive, megabase-sized locus composed of ∼171-bp alphoid repeating units (Willard and Waye, 1987). The flanking portions of the huge alphoid locus are occupied by other chromatin structures containing mostly normal H3 highly trimethylated on lysine 9 (H3K9me3), creating the so-called pericentric heterochromatin (Sullivan and Karpen, 2004, Grewal and Jia, 2007). This pericentric heterochromatin structure containing H3K9-metyltransferases is conserved in regional centromeres from yeast to human (e.g., *Schizosaccharomyces pombe*, Clr4; *Drosophila melanogaster*, SU(VAR)3–9; mouse and human, Suv39h1, and Suv39h2). Suv39h1 and Suv39h2 double knockout mice lose H3K9 trimethylation from the pericentric regions of the chromosomes (Peters et al., 2001). HP1 proteins that interact with Suv39 can bind to H3K9me3 modifications and to each other. These interactions are thought to spread the pericentric heterochromatin structure and silence adjacent gene expression, as exemplified by position-effect variegation (PEV) (Muller, 1930, Allshire et al., 1994, Talbert and Henikoff, 2006, Grewal and Jia, 2007). As a result, it is important to understand how these different chromatin clusters, CENP-A chromatin or H3K9me3 chromatin, assemble and maintain themselves on the same underlying simple repetitive DNA sequences (alphoid DNA in human).

Excessive heterochromatin spreading (invasion) can inactivate centromere function. De novo centromere chromatin assembly accompanied by pericentromeric heterochromatin can occur on alphoid DNA sequences containing the CENP-B binding motif (CENP-B box). Stable human artificial chromosomes (HACs) can be obtained by transfecting appropriate DNAs into human HT1080 cells and mouse embryonic fibroblasts (Harrington et al., 1997, Ikeno et al., 1998, Ohzeki et al., 2002, Ohzeki et al., 2015, Okada et al., 2007). A new version of the HAC (alphoid^tetO^-HAC) has also been generated using a synthetic alphoid array containing a tetracycline operator (tetO) sequence (the alphoid^tetO^ array) (Nakano et al., 2008). Tethering HP1 or Suv39h1 to the alphoid^tetO^-HAC, as a tetracycline repressor (tetR) fusion protein, abrogates centromere chromatin assembly on the HAC. Consequently, the alphoid^tetO^-HAC missegregates and forms micronuclei (Nakano et al., 2008, Cardinale et al., 2009, Ohzeki et al., 2012, Bergmann et al., 2012). The co-existence of centromere chromatin and heterochromatin at the same centromeric locus suggests the presence of intrinsic mechanisms that prevent the spread of heterochromatin into the centromere chromatin.

Histone acetyltransferase (HAT) activity antagonizes heterochromatin-mediated centromere inactivation. Many human cell lines, including HeLa cells, are incompetent for stable de novo centromere formation and have stronger H3K9-trimethylation activity on alphoid DNA than do HAC formation-competent HT1080 cells. We observed that tethering the HAT domain of p300 or PCAF protein (p300HD or PCAFHD) to the transfected alphoid^tetO^ DNA enabled active de novo centromere assembly and stable HAC formation, even in HeLa cells. Once established, those alphoid^tetO^-HACs required no further assistance from HAT tethering for maintenance of the centromere chromatin. Furthermore, temporally regulated chromatin acetylation activity has been detected on HAC centromeres, as well as natural centromeres, during G_1_ phase (Ohzeki et al., 2012). These results suggest that intrinsic HAT activity may be involved in the maintenance of established centromere chromatin, possibly by preventing strong heterochromatin from spreading onto centromeric alphoid DNA.

In each cell cycle, the amount of CENP-A nucleosomes is reduced by half during DNA replication and is not replenished until mitosis is complete (Jansen et al., 2007). Instead, they are replenished during the next G_1_ phase by the CENP-A-specific deposition factor HJURP (Foltz et al., 2009, Dunleavy et al., 2009). The Mis18 protein complex (consisting of hMis18α, hMis18β, and M18BP1), an upstream factor required for the assembly of HJURP at centromeres, temporarily localizes at the centromere from mitotic exit to G_1_ phase by interacting with CENP-C (Fujita et al., 2007, Maddox et al., 2007, Moree et al., 2011, Dambacher et al., 2012, McKinley and Cheeseman, 2014).

Interestingly, loss of CENP-A assembly following hMis18α depletion can be suppressed by treatment with the histone deacetylase inhibitor trichostatin A (TSA) (Fujita et al., 2007). Therefore, in the present study we hypothesized that the Mis18 complex may interact with specific HAT complexes at centromeres during early G_1_ phase. However, which of the 17 human HAT activities is involved in centromere chromatin assembly and maintenance remained to be determined. We screened Mis18 complex-associated acetyltransferases with a newly developed protein-protein interaction assay using the ectopic alphoid^tetO^ array integration site, and found the KAT7/HBO1/MYST2 to be an M18BP1-interacting partner. KAT7 localized at endogenous centromeres in G_1_ phase, and knocking out KAT7 reduced centromeric CENP-A assembly. A combination of knocking out KAT7 and overproducing Suv39h1 synthetically perturbed chromosomal segregation, leading to increased micronucleus formation. Furthermore, KAT7 tethered to a heterochromatinized alphoid^tetO^ site reduced H3K9me3 modifications and provided competence for new histone H3.3 or CENP-A assembly. Based on these results, we propose that KAT7 protects centromeres from the surrounding heterochromatin by promoting removal of H3 trimethylated on Lys9 via histone turnover/exchange activity. This activity may also contribute to increasing CENP-A deposition activity via interactions with M18BP1 scaffold in the canonical CENP-A replenishment pathway.

## Results

### Screening for an Intrinsic Acetyltransferase that Interacts with the Human Mis18 Complex

**Figure 1:**
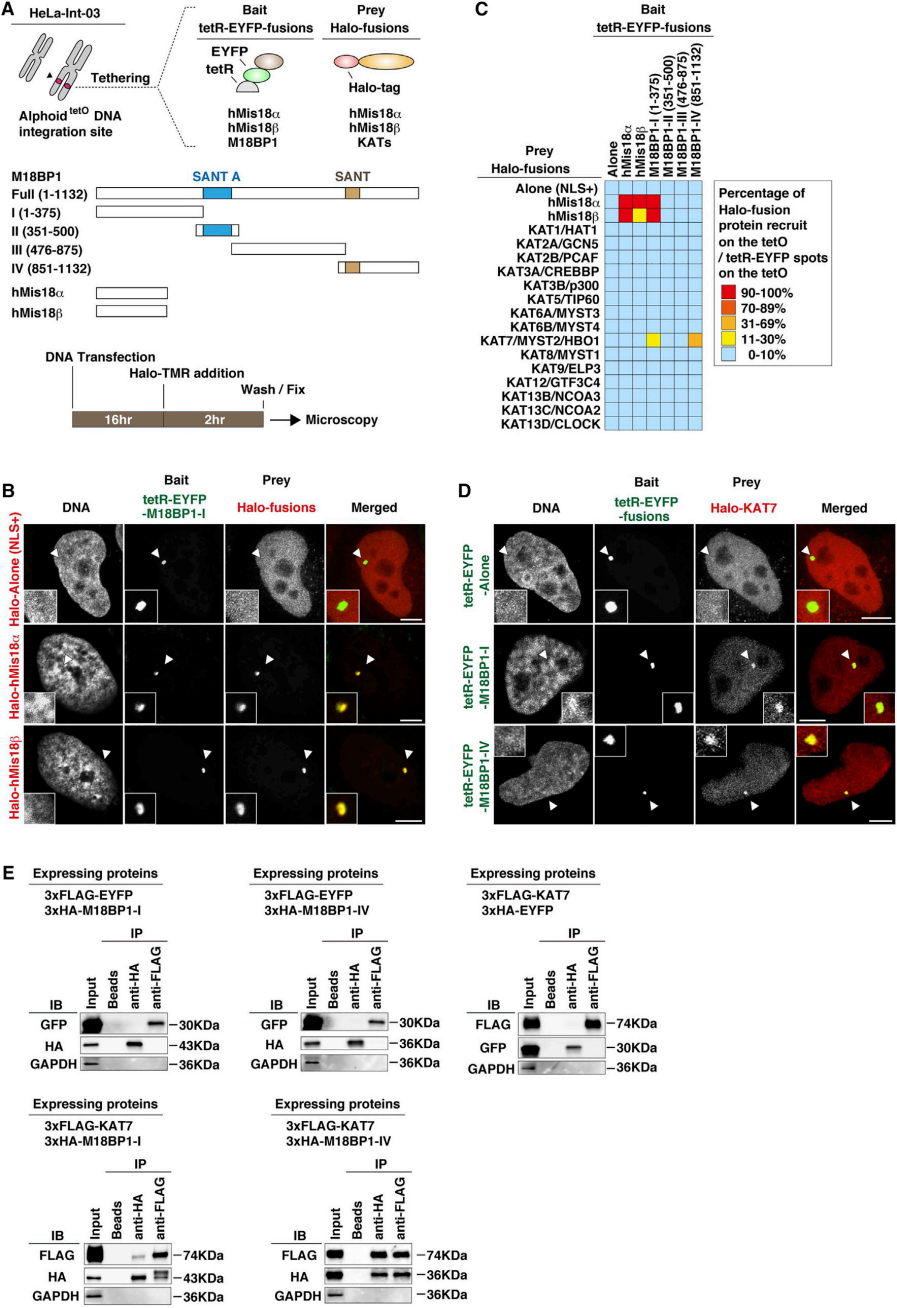
M18BP1 Recruits the Acetyltransferase KAT7/HBO1/MYST2 (A) HeLa-Int-03 cells have an ectopic integration site of alphoid^tetO^ DNA repeats, which contains ∼15,000 copies of the tetO sequences on a chromosome 21-derived synthetic alphoid DNA repeat. Halo-tag fused protein recruits were tested by tetR-fused protein tethering. (B) M18BP1 tethering recruits hMis18α or hMis18β. Fluorescence images were obtained with DAPI (DNA), EYFP (green), and Halo-TMR ligand (red). The Halo tag covalently binds to the Halo-TMR ligand via the enzymatic activity of the Halo tag. White arrowheads indicate the loci of tetR-EYFP-fusion protein spots on the alphoid^tetO^ DNA integration site. Scale bar, 5 μm. (C) Heatmap summarizing. Each cell indicates a combination of tetR-EYFP- and Halo-fusion proteins. Colors indicate the observed frequency of recruitment of Halo-fusion proteins by tetR-EYFP-fusion protein tethering (percentage of cells with a detectable signal). See also Figure S1A. (D) KAT7 recruited by M18BP1 tethering. Fluorescence images were obtained with DAPI (DNA), EYFP (green), and Halo-TMR ligand (red). White arrowheads indicate the loci of tetR-EYFP-fusion protein spots on the alphoid^tetO^ DNA integration site. Scale bar, 5 μm. (E) Co-immunoprecipitation experiments. Transiently expressed 3xFLAG-KAT7 and 3xHA-M18PB1 peptides were harvested in the cytosolic extract and used for immunoprecipitation (IP).

To explore and identify HATs that interact with the Mis18 protein complex, we carried out a fluorescence microscopy-based interaction-trap assay on human chromosomes. HeLa-Int-03 cells have an ectopic integration of alphoid^tetO^ DNA that lacks detectable CENP-A assembly and has a high level of H3K9me3 modification (Ohzeki et al., 2012). We tethered Mis18 complex subunits to this ectopic alphoid^tetO^ DNA integration site as tetR-EYFP fusions and tested whether candidate Halo-tag-fused “prey” proteins were recruited (Figure 1A).

In this assay, tetR-EYFP-fused M18BP1 was divided into four domains because full-length M18BP1 has a stronger affinity for centromeres than tetR does for the alphoid^tetO^ DNA integration site. With full-length M18BP1 it is difficult to distinguish which tetR-EYFP-M18BP1 spot is the signal coming from the alphoid^tetO^ DNA integration site. First, we confirmed the known interactions among Mis18 complex subunits. Using fluorescent microscopy, we detected Halo-hMis18α and Halo-hMis18β recruitment to the tethered M18BP1 amino-terminal domain (1–375) (Figure 1B). Reciprocal recruitment of hMis18α and hMis18β was also confirmed (Figure 1C) (Fujita et al., 2007). When we expressed 15 of the >17 known human lysine acetyltransferases (KATs) (Allis et al., 2007) as Halo-tag fusions, only KAT7 was recruited to tethered M18BP1 amino-terminal (1–375) and carboxyl-terminal (851–1,132) domains (Figures 1C, 1D, and S1A). KAT7 recruited the M18BP1 C-terminal domain and vice versa (Figure S1B). These protein interactions were confirmed by pull-down experiments (Figure 1E) and cytological analysis. Furthermore, EYFP-KAT7 co-localized with Halo-M18BP1 in HeLa cell nuclei (Figure S1C).

### Mis18 and KAT7 Complex Includes HJURP and Recruits RSF1

KAT7 was first identified as a HAT that binds to ORC (HBO1) (Iizuka and Stillman, 1999), and belongs to the MYST family protein complexes. Most of the known MYST complexes are heterotetramers containing the MYST domain HAT, an ING subunit, EAF6/CENP-28, and EPL1-like platform proteins (Doyon et al., 2006) (Figure S2A). KAT7 is recruited to the origin of replication and contributes to MCM2-7 loading in G_1_ phase (Miotto and Struhl, 2008). However, KAT7 function on centromere chromatin has not previously been described.

**Figure 2:**
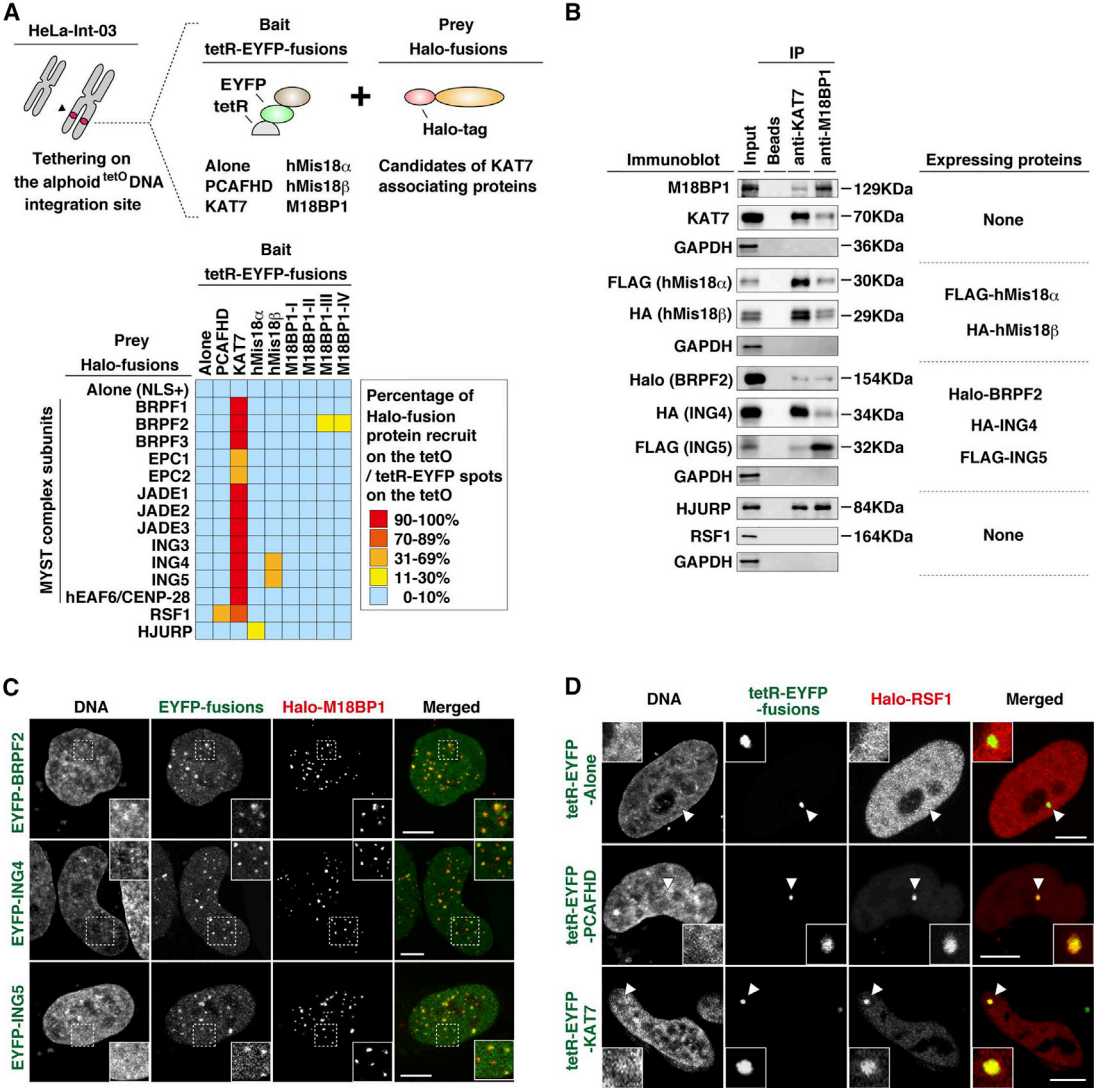
The M18BP1 and KAT7 Complex Associates with HJURP and RSF1 (A) Schematic diagram (top) and heatmap summarizing (bottom). Each cell indicates a combination of tetR-EYFP- and Halo-fusion proteins. See also Figures 1A, 1C, and S2C. (B) Cytosolic extract was harvested and used for immunoprecipitation with anti-KAT7 and anti-M18BP1 antibodies. Ectopically expressed tagged proteins in each experiment are indicated on the right. (C and D) EYFP-BRPF2, -ING4, or -ING5 co-localization with Halo-M18BP1 (C). HAT tethering recruits RSF1 (D). Fluorescence images were obtained with DAPI (DNA), EYFP (green), and Halo-TMR ligand (red). Arrowheads indicate the loci of tetR-EYFP-fusion protein spots on the alphoid^tetO^ DNA integration site. Scale bars, 5 μm.

We therefore tested whether KAT7 tethering to the alphoid^tetO^ DNA array recruits other MYST complex family members. Indeed, MYST complex subunits plus remodeling and spacing factor 1 (RSF1) were recruited in the KAT7 tethering assay (Figure S2B). Next, we tested whether these proteins and HJURP interact with Mis18 complex subunits (Figure 2A). BRPF2 was recruited by M18BP1 tethering, and ING4 and ING5 were recruited by Mis18β tethering. HJURP was recruited by hMis18α tethering (Figures 2A and S2C).

We also tested whether these recruited proteins co-immunoprecipitate with KAT7 or M18BP1 (Figures 2B and S2D). BRPF2, ING4, ING5, and HJURP co-immunoprecipitated with both KAT7 and M18BP1 (Figure 2B). Co-localization of EYFP-BRPF2, -ING4, or -ING5 with Halo-M18BP1 was also observed in cytological analysis (Figure 2C). Taken together, the results suggest that BRPF2, ING4, ING5, and HJURP interact with the Mis18 complex and KAT7.

Interestingly, RSF1 was also recruited by the PCAF HAT domain tethered at the ectopic site (Figures 2A and 2D), indicating that chromatin acetylation on the alphoid^tetO^ DNA promotes RSF1 binding.

### KAT7 Localizes to Centromeres in G_1_ Phase

Next, we asked whether tetR-EYFP-KAT7 and Halo-fused BRPF2, ING4, and ING5 co-localize with CENP-A at centromeres. The MYST proteins were distributed broadly throughout chromatin but, in a subpopulation of cells, showed some co-localization with CENP-A (Figure S3A). We then tested whether these proteins localize at centromeres in a cell-cycle-dependent manner.

**Figure 3:**
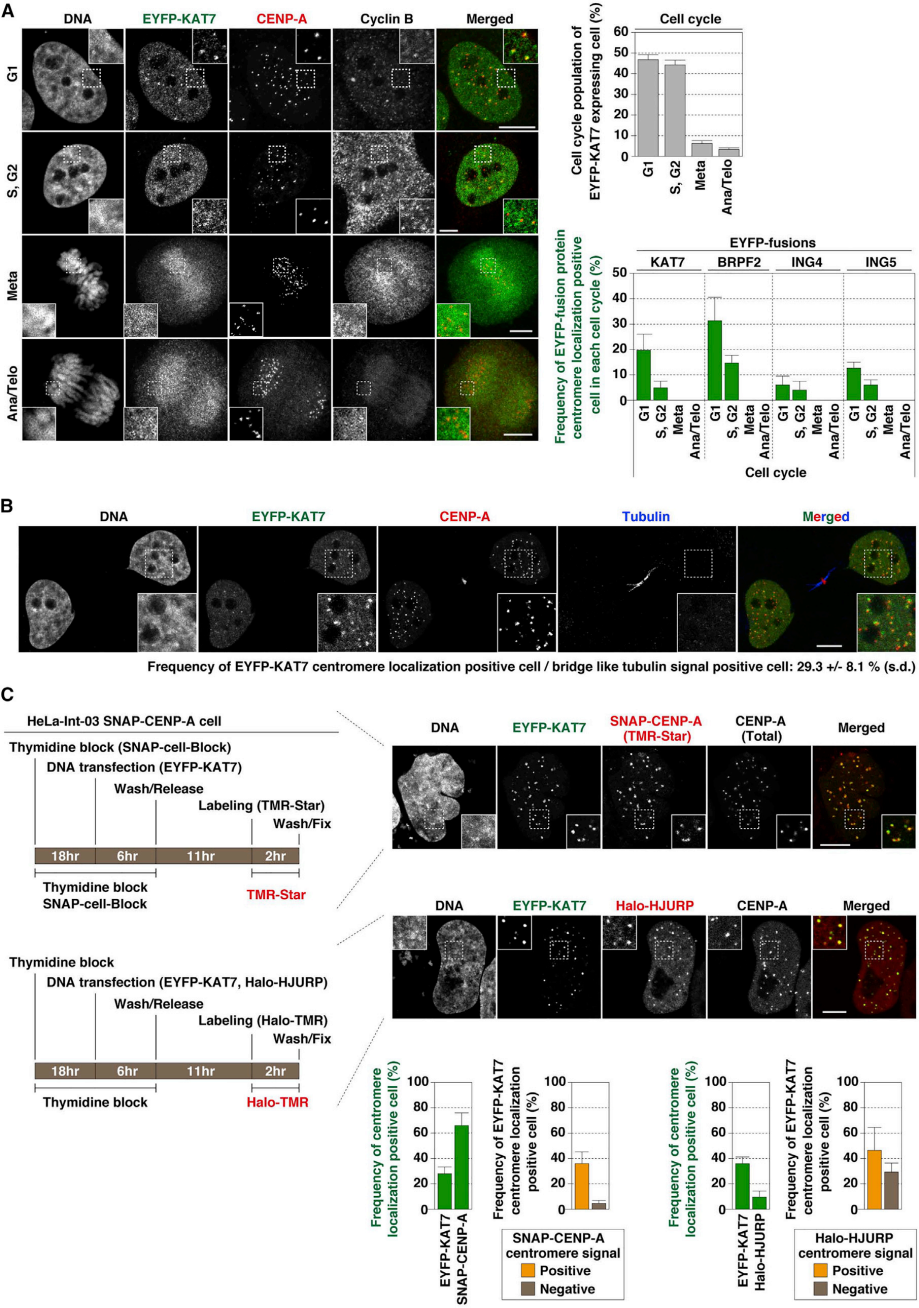
KAT7 and Other MYST Subunits Localize to the Centromere (A) KAT7 and CENP-A co-localization. Fluorescence images were obtained with DAPI (DNA), EYFP (green), anti-CENP-A antibody (red), and anti-cyclin B antibody. Cell cycles were distinguished by images of DAPI and cyclin B staining, and these distributions are shown in the upper right panel. The observed frequency of KAT7 centromere localization positive cells (i.e., co-localization of KAT7 and CENP-A) is plotted in the bottom right panel. EYFP-BRPF2, -ING4, and -ING5 were also analyzed. See also Figure S3B. (B) EYFP-KAT7 localized to centromeres at an earlier stage of G_1_ phase, using the presence of an intercellular bridge of tubulin as a marker for early G_1_ cells. Fluorescence images were obtained with DAPI (DNA), EYFP (green), anti-CENP-A antibody (red), and anti-tubulin antibody (blue). (C) KAT7 co-localization with new CENP-A and Halo-HJURP in G_1_ cells. (Left) Scheme for a quench chase pulse labeling of SNAP-CENP-A in G_1_ phase or G_1_ cell observation is shown on the left. Fluorescence images were obtained with DAPI (DNA), EYFP (green), TMR ligand (red; TMR-Star or Halo-TMR), and anti-CENP-A antibody. Data in this figure are presented as mean ± SD. (n = 3, >50 cells counted in each observation). Scale bars, 5 μm.

HeLa-Int-03 cells stably expressing EYFP-KAT7 (Int-03 + KAT7) were created and co-immunostained with anti-CENP-A antibody for centromeres and anti-cyclin B antibody as a cell-cycle marker. EYFP-KAT7 localized at centromeres in G_1_ phase (Figure 3A). Centromere localization of BRPF2, ING4, and ING5 in G_1_ phase was also observed in transient protein expression experiments (Figures 3A and S3B).

Indeed, KAT7 and CENP-A co-localized more frequently in early G_1_ cells having midbodies (29%) than in bulk G_1_ cells (20%) (Figures 3A and 3B). KAT7 also co-localized with newly assembled SNAP-CENP-A and Halo-HJURP (Figure 3C). These results suggest an involvement of KAT7 in the CENP-A assembly pathway.

### KAT7 Positively Regulates CENP-A Localization Level at Centromeres

We tested whether Mis18-KAT7 complex subunits were necessary for Halo-CENP-A localization at canonical centromeres using small interfering RNA (siRNA)-based protein depletion. Consistent with previous reports, M18BP1, hMis18α, hMis18β, and HJURP depletion substantially reduced Halo-CENP-A localization at the centromeres. Depletion of KAT7, BRPF2, ING4, and ING5 also reduced Halo-CENP-A localization at centromeres. Although these reductions were significant compared with the negative control, the effects were significantly weaker than those seen after HJURP depletion (Figure S4A).

**Figure 4:**
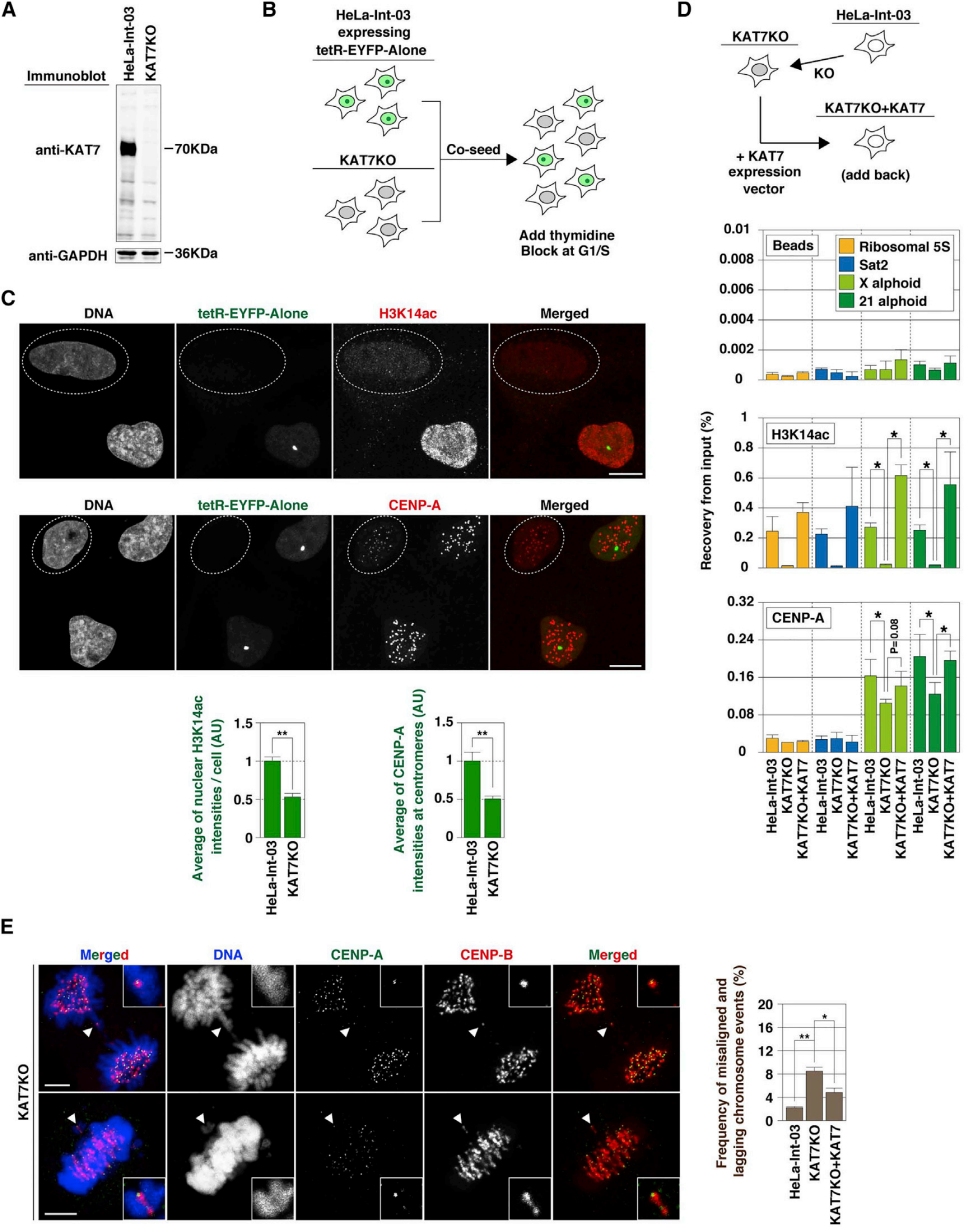
Centromeric CENP-A Assembly Was Reduced in KAT7-Knockout Cells (A) Total cell lysate was applied for KAT7 immunoblotting. GAPDH was a loading control. (B) Scheme of co-seeding. HeLa-Int-03 cells expressing tetR-EYFP alone were co-seeded with KAT7KO cells on a coverslip. The tetR-EYFP alone was used as a marker for distinguishing these cells. (C) Fluorescent image quantification. Co-seeded cells were stained with DAPI, EYFP (green), and anti-H3K14ac or anti-CENP-A antibody (red). The KAT7KO cells are circled with white dotted lines. Scale bar, 10 μm. Signal intensities of the total nuclear H3K14ac (mean ± SE, n ≥ 50) and CENP-A at each centromere focus (mean ± SE, n ≥ 60) are shown at the bottom. (D) ChIP analysis. ChIP was carried out with anti-H3K14ac antibody, anti-CENP-A antibody, or beads alone (no antibody). Data are presented as mean ± SD (n = 3). See also Figure S4D. (E) Chromosome missegregation. Mitotic cells were stained with DAPI (DNA; blue), anti-CENP-A antibody (green), and anti-CENP-B antibody (red). White arrowheads indicate lagging or misaligned chromosomes. Scale bar, 5 μm. Frequencies of misaligned and lagging chromosome events were plotted (>100 metaphase cells were counted in each cell line). Data are presented as mean ± SE. (n = 6). ^∗^p < 0.05, ^∗∗^p < 0.005 (t test).

To further investigate human KAT7 function in centromeres, we knocked out KAT7 in HeLa-Int-03 cells using the CRISPR/Cas system and isolated cell lines lacking KAT7 expression (KAT7KO; Figure 4A). Although KAT7 is essential for mouse post-gastrulation development, KAT7-knockout embryonic and immortalized fibroblasts are viable. A typical feature of mouse KAT7-knockout cells is reduced acetylation of the histone H3-K14 (H3K14ac) (Kueh et al., 2011). We checked H3K14ac levels by immunostaining and confirmed a strong reduction of nuclear H3K14ac signals in comparison with the parental HeLa-Int-03 cells in mixed cultures (Figures 4B, 4C, and S4B).

In chromatin immunoprecipitation (ChIP) assays, a strong reduction of H3K14ac levels was detected on chromosome X and 21 centromeric alphoid DNAs, ribosomal 5S DNA, and pericentromeric satellite DNA (Sat2) in KAT7KO cells. Levels of H3K14ac were rescued by *KAT7* expression in KAT7KO cells (Figure 4D). These results, together with the previous reports indicating that centromeric H3K14ac levels are low in fission yeast, fly, and human (Mellone et al., 2003, Sullivan and Karpen, 2004), suggest that H3K14 acetylating activity might be temporally regulated. To test this hypothesis we carried out ChIP with synchronized cells, and confirmed that H3K14ac levels on alphoid DNA are higher in early G_1_ phase (Figure S4D).

We quantitated and compared centromeric CENP-A localization levels between KAT7KO cells and the parental HeLa cells in mixed culture. CENP-A levels were reduced by half at the endogenous centromeres in KAT7KO cells (Figures 4B, 4C, S4B, and S4C). The reduction of CENP-A levels on the chromosome 21 alphoid DNA was rescued by *KAT7* overexpression. The timing of new CENP-A localization at centromeres was similar in KAT7KO and HeLa-Int-03 cells (Figure S4E). However, the relative intensity of CENP-A at centromeres was significantly reduced (Figure S4F). These results suggest that KAT7 positively controls CENP-A assembly at centromeres.

Such a reduction of CENP-A level at centromeres might cause chromosome instability. We therefore evaluated chromosome instability by counting the frequencies of lagging and misaligned chromosomes in mitotic cells. Compared with HeLa-Int-03 cells, lagging and misaligned chromosomes were significantly (3.8-fold) increased in KAT7KO cells (Figure 4E). This instability was suppressed by expressing exogenous KAT7 (KAT7KO + KAT7 cells).

### KAT7 Absence and Suv39h1 Overproduction Synthetically Induce Centromere Dysfunction

**Figure 5:**
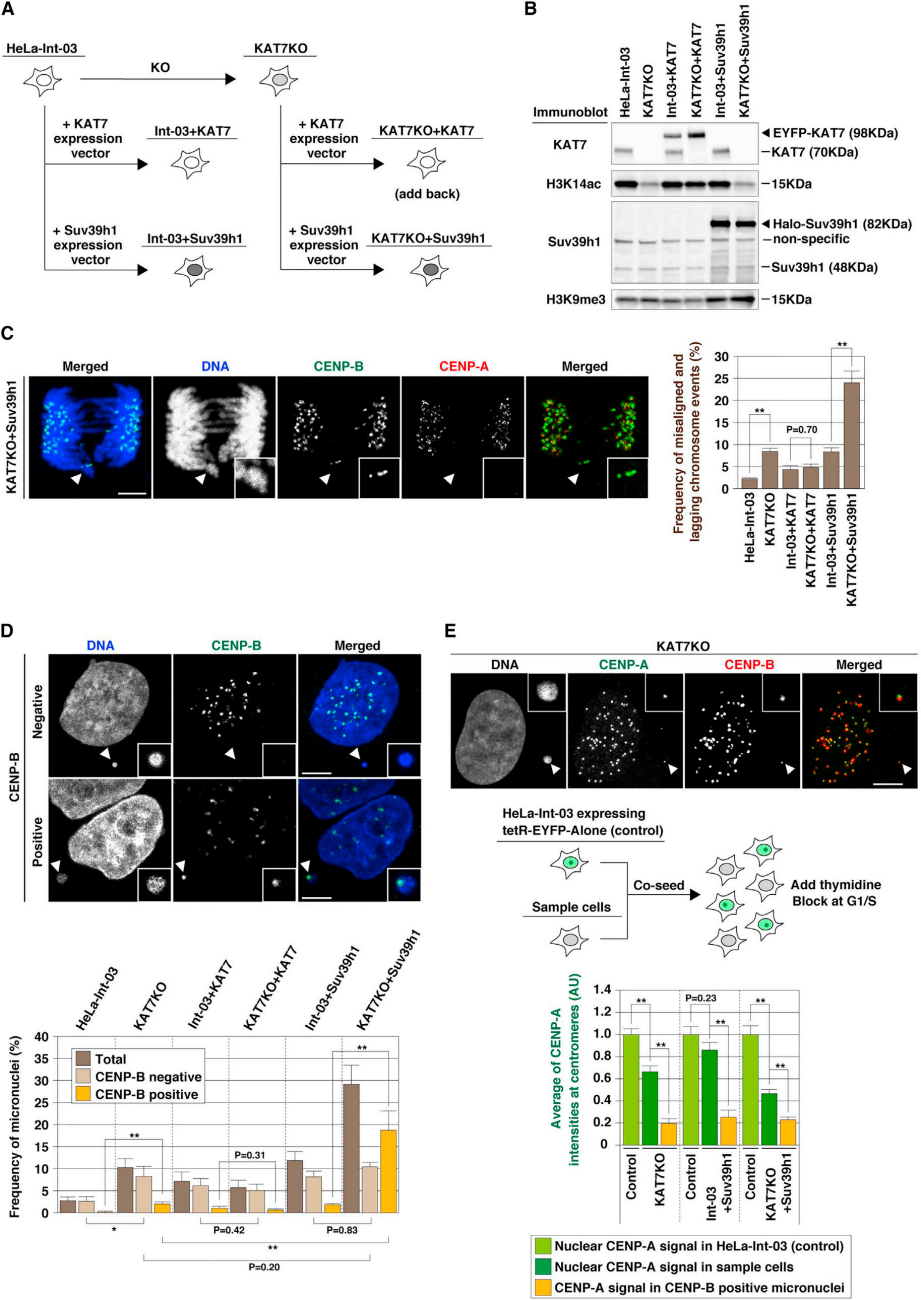
Combination of KAT7 Absence and Suv39h1 Overproduction Synthetically Destabilizes Chromosome Segregation (A) Graphic explanation of the cell lines. (B) Total cell lysate was applied for immunoblotting. (C) Frequencies of misaligned and lagging chromosomes. Cells were stained with DAPI (DNA; blue), anti-CENP-B antibody (green), and anti-CENP-A antibody (red). White arrowheads indicate a lagging chromosome. Frequencies of the misaligned and lagging chromosomes were plotted (>100 metaphase cells were counted in each cell line). Data are presented as mean ± SE (n ≥ 3). (D) Frequencies of the CENP-B-positive and -negative micronuclei. Cells were stained with DAPI (DNA; blue) and anti-CENP-B antibody (green). White arrowheads indicate the locations of micronuclei. Frequencies of the micronuclei were plotted (>200 cells were counted in each cell line). Data are presented as mean ± SD (n = 3). (E) CENP-A intensity at centromeres, including CENP-B-positive micronuclei. Cells were stained with DAPI, anti-CENP-A antibody (green), and anti-CENP-B antibody (red). White arrowheads indicate the locations of micronuclei. Control (HeLa-Int-03 expressing tetR-EYFP) and target cells were co-seeded on coverslips, and arrested at G_1_/S with thymidine. CENP-A signal at centromeres including the CENP-B-positive micronuclei was quantitated and plotted in the bottom panel. Data are presented as mean ± SE (n ≥ 10 micronuclei). ^∗^p < 0.05, ^∗∗^p < 0.005 (t test). Scale bars, 5 μm.

Centromeres are flanked by heterochromatin, and we previously showed that excess heterochromatin is incompatible with centromere maintenance and CENP-A assembly (Nakano et al., 2008, Cardinale et al., 2009). We therefore investigated whether KAT7 helps centromeres to resist invasion by heterochromatin by creating HeLa-Int-03 and KAT7KO cells overexpressing Suv39h1 (Figures 5A, 5B, and S5A). Suv39h1 tethering to the alphoid^tetO^-HAC destabilized its centromere function, resulting in a high HAC loss rate (∼22% per cell division) (Ohzeki et al., 2012). Suv39h1 is responsible for pericentric H3K9me3 modification in mice (Peters et al., 2001) and enhances H3K9me3 levels on alphoid DNAs in human HT1080 cells (Ohzeki et al., 2012). Therefore, we anticipated a possible synthetic effect of the absence of KAT7 and Suv39h1 overproduction in centromere function.

We quantitated chromosome stability by counting the frequencies of lagging and misaligned chromosomes in mitotic cells. Suv39h1 overproduction (Int-03 + Suv39h1 cells) also increased misaligned chromosomes by 3.6-fold. Strikingly, in KAT7KO + Suv39h1 cells, misaligned chromosomes were observed 10-fold more frequently, and some of the misaligned or lagging chromosomes were negative for centromeric CENP-A (Figure 5C).

Missegregated chromosomes often form micronuclei. The frequency of micronucleus formation correlated with that of misaligned chromosomes, and both loss of KAT7 and Suv39h1 overproduction synthetically increased micronucleus formation. In KAT7KO, Int-03 + Suv39h1, and KAT7KO + Suv39h1 cells, micronucleus formation was increased by 3.7-, 4.4-, or 11-fold, respectively, compared with HeLa-Int-03 cells (Figure 5D).

Micronucleus formation also occurs subsequent to DNA breakage, particularly if the breakage produces an acentric chromosome fragment. KAT7 depletion could lead to DNA breakage, as KAT7 also contributes to DNA replication, and increased Suv39h1 protein levels harm DNA repair in the heterochromatin (Liu et al., 2013). Therefore, to investigate whether micronucleus formation was caused by a loss of centromere DNA or function, we determined the frequency of CENP-B-positive and -negative micronuclei. CENP-B can bind both active and inactive centromeres on alphoid DNA (Earnshaw et al., 1989) through the CENP-B box (Masumoto et al., 1989). Therefore, CENP-B-positive micronuclei would be formed by events in which a centromere was present but had become inactivated, and thus presumably not as a result of chromosome breakage. In HeLa-Int-03 cells, most micronuclei were negative for CENP-B. In KAT7KO and Int-03 + Suv39h1 cells, most micronuclei were CENP-B negative (3.2-fold and 3.9-fold increased versus HeLa-Int-03, respectively), but a significant population of CENP-B-positive micronuclei was also observed (13-fold [p < 0.005] and 12-fold [p < 0.005] increase for KAT7KO and Int-03 + Suv39h1 versus HeLa-Int-03, respectively). Interestingly, in KAT7KO + Suv39h1 cells, CENP-B-positive, but not -negative micronuclei were significantly increased compared with levels seen after KAT7KO (positive micronuclei, 9.3-fold increase [p < 0.005]; negative micronuclei, 1.3-fold increase [p = 0.2]) and Int-03 + Suv39h1 cells (positive micronuclei, 10-fold increase [p < 0.005]; negative micronuclei, 1.0-fold increase [p = 0.83]) (Figure 5D). We confirmed the reduction of centromeric CENP-A intensity (>74% reduction) at the CENP-B-positive micronuclei in all of these cases (Figure 5E).

Thus, either the absence of KAT7 or Suv39h1 overproduction predominantly results in formation of CENP-B-negative micronuclei, presumably due to defects in DNA replication or repair. However, the combination of KAT7 depletion and Suv39h1 overproduction increases CENP-B-positive micronuclei, presumably reflecting a loss of centromere function due to heterochromatin spreading. We detected a 1.2- to 1.3-fold increase in H3K9me3 levels on alphoid DNAs in KAT7KO cells (Figure S5B). Thus, one function of KAT7 may be to antagonize the spread of heterochromatin.

### KAT7 and RSF1 Antagonize H3K9me3 Modification and Contribute to Centromere Stability

A combination of KAT7 absence and Suv39h1 overproduction synthetically affects chromosome instability (Figure 5). This could be explained by the reduction in centromeric CENP-A assembly caused by the absence of KAT7, if that increases centromere sensitivity to Suv39h1-mediated heterochromatin invasion. We therefore decided to test whether KAT7 and its associated proteins can antagonize H3K9 trimethylation. We focused on the non-centromeric ectopic alphoid^tetO^ DNA integration site because it has high levels of H3K9me3 (Ohzeki et al., 2012).

**Figure 6:**
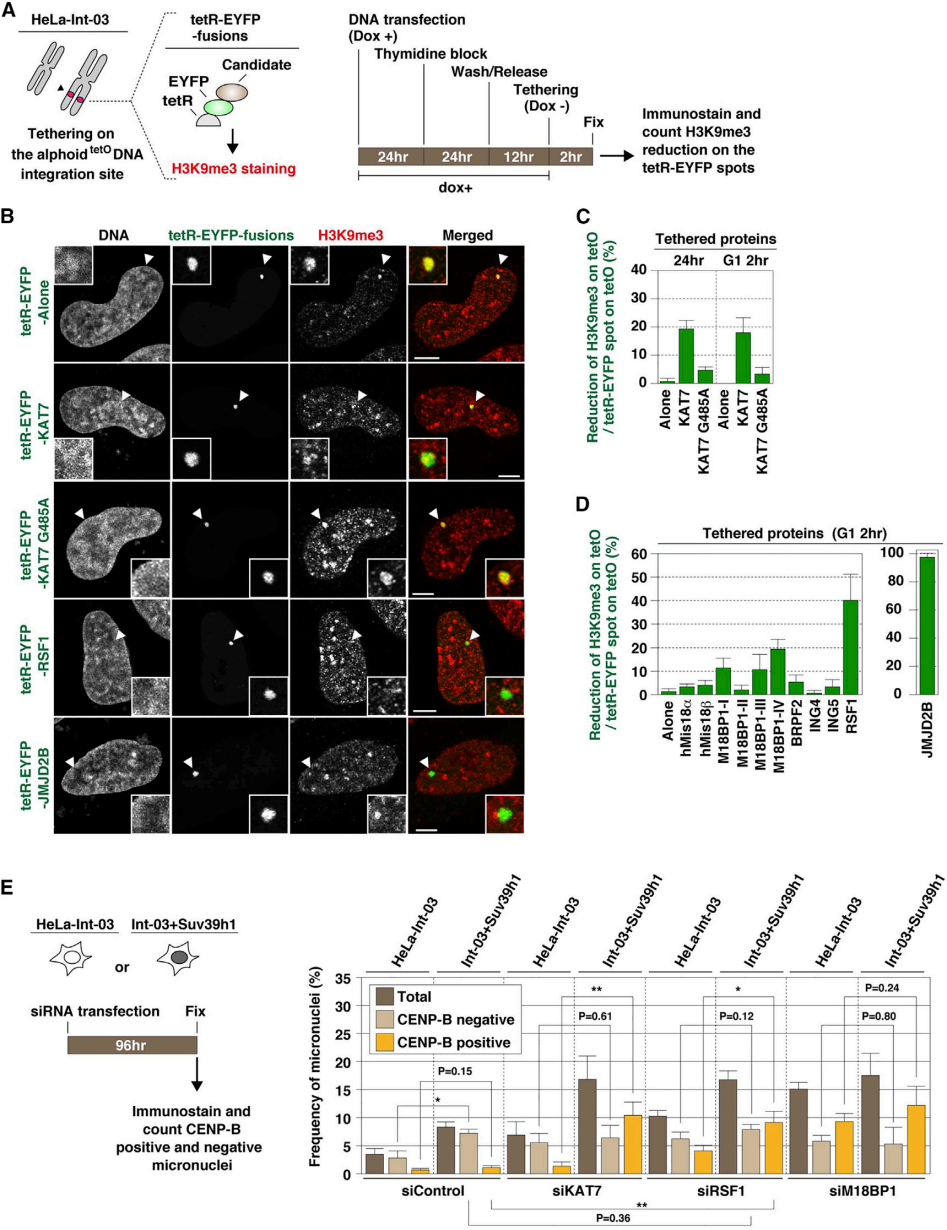
KAT7 and RSF1 Tetherings Reduce H3K9me3 Modification (A) Scheme of candidate protein tethering and H3K9me3 immunostaining in G_1_ phase. (B) Examples of H3K9me3 staining. Fluorescence images were obtained with DAPI (DNA), EYFP (green), and anti-H3K9me3 antibody (red). Arrowheads indicate loci of tetR-EYFP-fusion protein spots on the alphoid^tetO^ DNA integration site. Scale bars, 5 μm. (C and D) Observed H3K9me3 reduction frequency was plotted (>50 tetR-EYFP spots were counted in each sample). Data are presented as mean ± SD (n = 3). (E) Scheme of micronuclei formation combined with siRNA transfection (left). Cells were stained and observed as shown in Figure 5D. The frequencies of the CENP-B-positive and -negative micronuclei were plotted (>200 cells were counted in each cell line). Data are presented as mean ± SD (n = 3). ^∗^p < 0.05, ^∗∗^p < 0.005 (t test).

Tethering tetR-EYFP alone did not affect the bright H3K9me3 staining pattern at the ectopic alphoid^tetO^ site. In almost all observed cells (99%), the tetR-EYFP spot clearly overlapped with the H3K9me3 signal. In contrast, tethering tetR-EYFP-KAT7 reduced the H3K9me3 signal in 19% of tetR-EYFP spots (Figures 6A and 6B). In controls, tethering the tetR-EYFP-KAT7 G485A acetyltransferase catalytic domain mutant was less effective than the normal KAT7 (4.7%). No significant difference was found in the frequencies between 2 hr of tethering in cells synchronized at G_1_ phase and 24 hr of continuous tethering in asynchronously cultured cells (Figure 6C). Thus, the reduction in H3K9me3 appears to be independent of effects on DNA replication.

We counted the number of cells exhibiting reduced H3K9me3 staining following tethering of several tetR-EYFP-fusion proteins in G_1_ phase. Tethered BRPF2, ING4, or ING5 were less potent than tethered KAT7. M18BP1 domain IV tethering reduced H3K9me3 levels to an extent similar to that seen after tethering KAT7 (Figure 6D). Thus, the reduction in H3K9me3 was possibly due to endogenous KAT7 recruitment. Interestingly, RSF1 was the most potent factor at reducing H3K9 methylation (by 40%) (Figure 6D). The H3K9me3 reduction by KAT7 and RSF1 was also confirmed by ChIP-qPCR analysis (Figure S6A). We also tethered tetR-EYFP-JMJD2B, a H3K9me3 demethylase, to the alphoid^tetO^ DNA integration site. As expected, JMJD2B reduced H3K9 trimethylation (Figures 6B and 6D).

We next investigated the role of RSF1 as a factor reducing H3K9me3 using cells overexpressing Suv39h1 to induce heterochromatin spreading, and measuring CENP-B-positive micronuclei as a measure of loss of centromere function. Comparing HeLa-Int-03 and Int-03 + Suv39h1 cells, RSF1 depletion significantly increased CENP-B-positive micronucleus formation (2.6-fold, p < 0.05), but not CENP-B-negative micronuclei (1.3-fold increase, p = 0.12) (Figure 6E). Next, centromeric CENP-A signal intensities both in nuclei and CENP-B-positive micronuclei were quantitated. RSF1 depletion and Suv39h1 overproduction synergistically reduced normal centromeric CENP-A levels in nuclei (45% reduction), and CENP-A reduction was even more pronounced in the CENP-B-positive micronuclei (81% reduction) (Figure S6B). These data suggest that RSF1 depletion synthetically destabilizes centromere function in the presence of Suv39h1 overproduction. A similar synthetic destabilization was confirmed following KAT7 depletion (Figure 6E). On the other hand, M18BP1 depletion, which disrupts the CENP-A assembly machinery (Figure S4A), destabilized centromere function at a similarly high level independent of Suv39h1 overproduction (Figures 6E and S6B).

### KAT7 and RSF1 Promote Histone Exchange/Turnover

**Figure 7:**
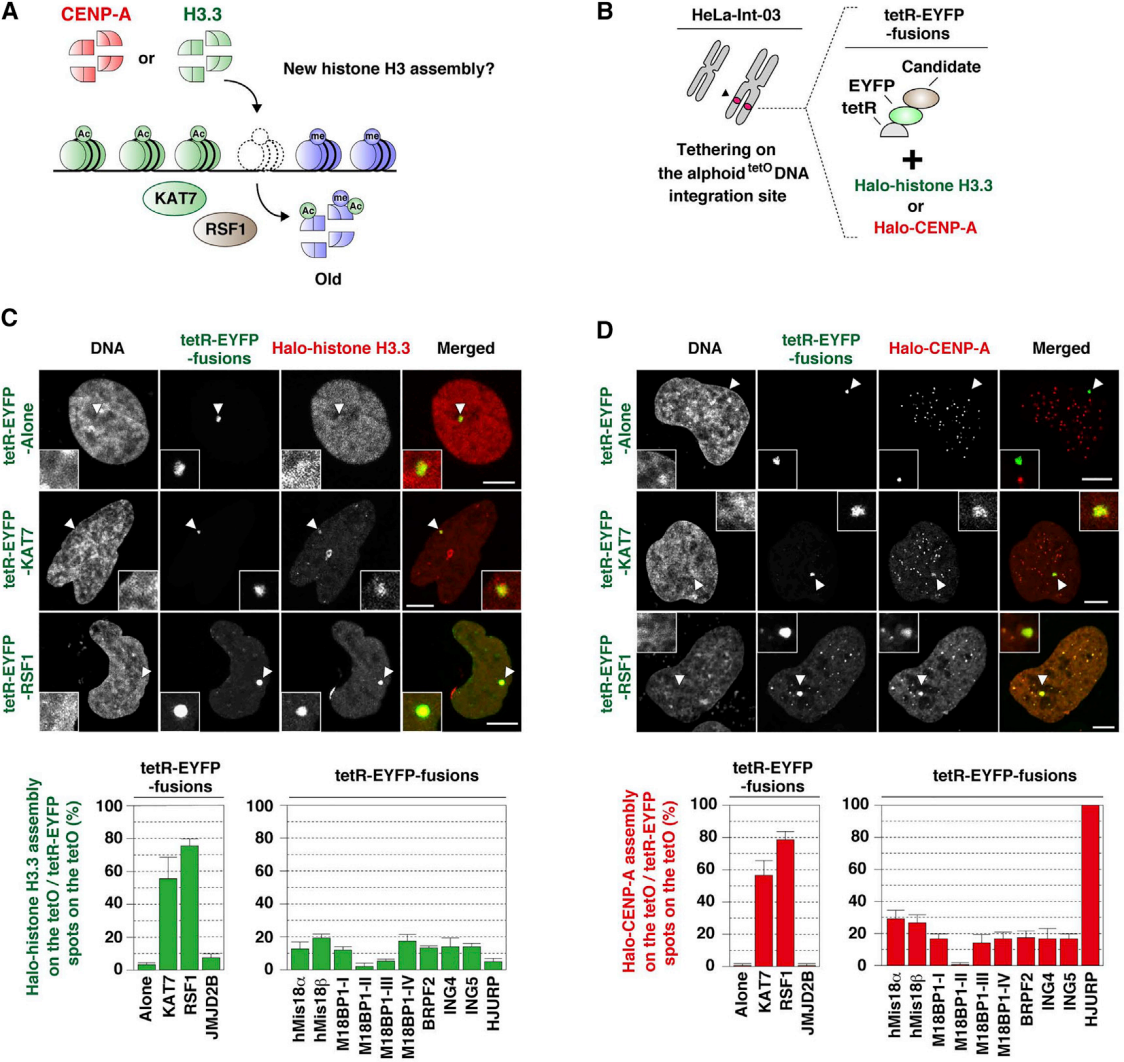
KAT7 and RSF1 Promote Histone Exchange/Turnover (A) A model for histone turnover. Note that KAT7 acetylates not only histone H3 but also H4 (Miotto and Struhl, 2010). (B) Scheme for experiments. (C and D) Fluorescence images were obtained with DAPI (DNA), EYFP (green), and Halo-TMR ligand (red). The Halo-histone H3.3 (C) or -CENP-A (D) assembly frequency was plotted (>50 tetR-EYFP spots were counted in each sample). Data are presented as mean ± SD (n = 3). Arrowheads indicate loci of tetR-EYFP-fusion protein spots on the alphoid^tetO^ DNA integration site. Scale bars, 5 μm.

The observed H3K9me3 reduction could be explained by demethylation at the H3-K9 residue or histone H3 eviction by a histone turnover mechanism. To detect histone H3 turnover, we focused on the histone H3 variant, H3.3, because its deposition is independent of DNA replication (Szenker et al., 2011). We also tested whether newly expressed Halo-histone H3.3 assembles at the alphoid^tetO^ DNA integration site following tetR-EYFP-KAT7 or tetR-EYFP-RSF1 tethering (Figures 7A–7C). Halo-histone H3.3 efficiently assembled after both KAT7 and RSF1 tethering in G_1_ phase (Figure 7C). When Halo-CENP-A was expressed, it was similarly assembled to both KAT7 and RSF1 tethering sites (Figure 7D). Although KAT7 or RSF1 tethering promoted CENP-A and histone H3.3 assembly, neither histone H3.3 nor CENP-A assembly was induced solely by the reduction of H3K9 trimethylation (i.e., JMJD2B tethering) (Figures 7C and 7D). These observations suggest that KAT7 or RSF1 tethering at the alphoid^tetO^ DNA integration site promoted histone turnover/exchange, resulting in H3.3 and CENP-A assembly (see also Shono et al., 2015).

Together, these results suggest that KAT7 and RSF1 prevent Suv39h1-induced heterochromatin invasion, possibly via a histone turnover/exchange mechanism.

## Discussion

In this study, we identified KAT7 as a HAT that interacts with M18BP1 to positively regulate centromeric CENP-A assembly and prevent Suv39h1-mediated centromere inactivation via a mechanism that involves increased histone turnover/exchange mediated by the Mis18 complex interacting with other factors.

The Mis18 complex is critical for epigenetic centromere maintenance, especially in CENP-A replenishment. In this study, we show that the M18BP1 amino-terminal region recruited hMis18α and hMis18β while its C-terminal region recruited KAT7 and BRPF2. Both hMis18α and hMis18β are involved in the efficient HJURP-CENP-A assembly pathway. Tethering KAT7 to the alphoid^tetO^ DNA resulted in histone turnover/exchange activity. In KAT7KO cells, centromeric CENP-A assembly levels decreased by half. Thus, coordinated assembly of HJURP and KAT7 on the same M18BP1 scaffold via other interacting proteins may be necessary for normal CENP-A deposition at centromeres. Interestingly, tethered KAT7 recruited RSF1 to the acetylated chromatin. RSF1 reportedly co-purifies with CENP-A nucleosomes and contributes to stable CENP-A assembly (Perpelescu et al., 2009). RSF1 recruited by KAT7 may also regulate centromeric CENP-A assembly during Mis18 complex formation, as RSF1 tethering strongly promotes H3.3 and CENP-A turnover/exchange.

PEV around pericentric heterochromatin is thought to result from a stochastic balance between heterochromatin spreading and its containment. In fission yeast, the cluster of tRNA genes between the centromere core (cnt) and pericentric (dg and dh) DNA functions as a chromatin boundary to prevent such heterochromatin spread. Deletion of those tRNA genes causes chromosomal instability, resulting from reduced CENP-A^Cnp1^ assembly (Scott et al., 2006). In *D. melanogaster*, GAGA factor and FACT achieve transcription-coupled histone H3.3 turnover at their binding sites located between the white gene and pericentric heterochromatin. This H3.3 replacement counteracts spreading of the H3-K9-methylated chromatin (Nakayama et al., 2007). Thus, heterochromatin spreading can be prevented by transcriptional activity or associated histone turnover.

Although no obvious boundary DNA sequence element akin to fission yeast tRNA genes has been identified between the centromere and heterochromatin on the long alphoid repetitive arrays in human cells, we found that KAT7 acetyltransferase (in the MYST2 HAT complex) was recruited by M18BP1. Tethering KAT7 or RSF1 to an ectopic non-centromeric alphoid^tetO^ DNA array was sufficient to reduce the level of H3K9me3 modification, and this reaction was coupled with new H3.3 or CENP-A assembly. Depletion of KAT7 or RSF1 combined with Suv39h1 overproduction resulted in centromere dysfunction.

These results strongly suggest that M18BP1 recruits KAT7 to the centromere to prevent Suv39h1-mediated heterochromatin spreading both via acetylation and as a result of removal of H3K9me3 nucleosomes by histone turnover/exchange mechanisms associated with RSF1. Interestingly, fission yeast KAT7^Mst2^ has been shown to antagonize pericentric heterochromatin nucleation by promoting transcription (Reddy et al., 2011) and Suv39h1^Clr4^-mediated gene silencing (Wang et al., 2015). In human mesenchymal stem cells, loss of Suv39h1 methyltransferase activity increases the amount of alphoid DNA transcripts (Zhang et al., 2015). In addition, RSF1 enhances transcription on acetylated chromatin but not heterochromatic templates in vitro (Loyola et al., 2001). Tethering KAT7 or RSF1 to the alphoid^tetO^ array indeed enhanced its transcription level by 1.7- to 2.1-fold (Figure S7B). Transcriptional events and/or KAT7 assembly on alphoid DNAs could stimulate histone turnover. Interestingly, transcription-coupled CENP-A incorporation events in *D. melanogaster* and *S. pombe* have recently been reported (Chen et al., 2015, Catania et al., 2015).

Simultaneous absence of KAT7 and overproduction of Suv39h1 caused a synthetic chromosome missegregation phenotype. Possible mechanisms for H3K9me3-mediated centromere inactivation have been suggested by previous studies. Tethering H3-K9 trimethylases or HP1 induces the sequential disassembly of centromere proteins. CENP-H is lost, followed by CENP-C and CENP-A (Nakano et al., 2008, Cardinale et al., 2009). Heterochromatin invasion of the centromere chromatin apparently destabilizes these centromere proteins and may eventually inactivate the kinetochore assembly and/or CENP-A replenishment pathway. Although H3K9me3 can promote centromere inactivation, it is observed by super-resolution microscopy between CENP-A-containing nucleosomes, even in a functional centromere (Ribeiro et al., 2010). Possibly, isolated patches of H3K9me3 may be permissive for centromere function unless HP1 assembles at this site (Martins et al., 2015). KAT7 or RSF1 could remove the H3K9me3 marks before HP1 assembly and/or CENP-A replenishment.

In the competition between centromere and heterochromatin assembly, alphoid DNA sequences may also play important roles. KAT7 and RSF1 tethering assembled CENP-A on the alphoid^tetO^ DNA but not on a synthetic non-alphoid DNA^tetO^ array, RF322^tetO^ (Ohzeki et al., 2002) (Figure S7C). Some properties of alphoid DNA, such as the CENP-B box/CENP-B interaction, are necessary for this CENP-A assembly activity (Okada et al., 2007). CENP-B stabilizes preassembled CENP-A, and recruits CENP-C for CENP-A and kinetochore assembly (Suzuki et al., 2004, Fachinetti et al., 2013, Fachinetti et al., 2015, Fujita et al., 2015).

On the other hand, CENP-B can also efficiently heterochromatinize the ectopically integrated alphoid DNA in mouse chromosomes via interactions with Suv39h1 (Okada et al., 2007). Disrupting heterochromatin by H3K9 acetylation is required for de novo CENP-A and HAC assembly on satellite DNAs in heterochromatin-rich cells (Ohzeki et al., 2012). Acetylation activity on alphoid DNA-associated chromatin might bypass the requirement for hMis18α in CENP-A accumulation. As previously reported, TSA treatment suppresses CENP-A centromere localization defects in cells lacking hMis18α (Fujita et al., 2007), and we confirmed that KAT7 overproduction also bypasses the requirement for hMis18α (Figure S7D). Although it is not known how acetylation bypasses the requirement for hMis18α, alphoid DNA and CENP-B might function in acetylation-induced CENP-A assembly. It is also interesting to speculate whether the acetylation and histone turnover/exchange can switch between the heterochromatin-antagonizing and heterochromatin-promoting properties of CENP-B.

Centromere DNA sequences are widely divergent between species. Although once established the centromere is epigenetically maintained through CENP-A replenishment mechanisms, it is not known how current centromeres were originally formed at their present genomic loci de novo. We recently found that CENP-C (or CENP-I) tethering is sufficient for de novo endogenous CENP-A assembly on the ectopically integrated alphoid^tetO^ array. This de novo endogenous CENP-A assembly pathway requires M18BP1. However, tethering of CENP-A replenishment factors (M18BP1, hMis18α, hMis18β, KAT7, or RSF1) was insufficient to promote de novo endogenous CENP-A assembly (Shono et al., 2015), even though those replenishment factors were sufficient for de novo assembly of overexpressed CENP-A at the ectopic site. Interestingly, in those experiments HJURP tethering could promote endogenous CENP-A assembly, indicating that the CENP-A pool size was not a limiting factor for de novo assembly. Taken together, these results suggest that for de novo endogenous CENP-A assembly, some properties of CENP-C (or CENP-I) as an epigenetic and a functional centromere mark are also necessary in addition to the recruitment of CENP-A replenishing factors by M18BP1, although CENP-A overproduction or forced DNA binding of HJURP via tetR can bypass this constraint. Further studies are required to determine how centromere integrity is established and maintained by these CENP-A de novo assembly and replenishing factors.

## Experimental Procedures

### Cell Culture, Transfection and Staining

Human cultured cells, 293T, HeLa, HeLa-Int03, and HeLa-Int-03 derivative cells were grown in a DMEM Glutamax I (Invitrogen), supplemented with 10% fetal bovine serum at 37°C in a 5% CO_2_ atmosphere. For transfections, Lipofectamine 2000 (Invitrogen) or FuGENE HD (Promega) transfection reagent was used for siRNA or plasmid vector DNA, respectively. Used siRNA sequences and plasmid vectors are provided in Tables S1 and S2. The cell lines used in this study are provided in Table S3. Specifically, HeLa-Int-03 derived cell lines expressing EYFP-KAT7 or Halo-Suv39h1 were produced with the Jump-in integration system (Life Technologies) using pJ3-EYFP-KAT7 or pJ4IB-Halo7-Suv39h1 gene-expression vector by co-transfection with pJTI PhiC31 Int vector (expression vector for Phi-C31 integrase; Life Technologies). *KAT7* gene knockout was carried out with a CRISPR/Cas9-mediated gene deletion method by transient co-transfection of plasmids pJ4IB-Cas9 and pU6CR-KAT7 (expression vector for Cas9 protein and guide RNA against *KAT7* gene, respectively). The targeted sequence of *KAT7* gene was 5′-GACATGTCCCTGAAGGACTCAGG. In isolated single clones, *KAT7* gene deletions with frameshift mutations at all the alleles were confirmed by sequencing of genomic PCR products using an MiSeq sequencer (Illumina) (more than 9,000 reads were obtained in each sample). The PCR primer set used for *KAT7* gene locus was 5′-CCATGATTAGCAACTCAGGAGAAATACAG and 5′-CAGGGTCTCATGTGTACTCCTAATTTC, respectively. For Halo-tag labeling, Halo-TMR ligand (Promega, G8251) was added to the cell-culture medium (1 nM). For SNAP-tag labeling, 3 μM of SNAP-Cell Block reagent (NEB) was used for blocking of pre-existing SNAP-fused protein, and 1 μM of SNAP-Cell TMR-Star reagent (NEB) was used for newly expressed SNAP-fused protein staining.

### ChIP and qPCR

The main ChIP procedure was described previously (Ohzeki et al., 2002, Ohzeki et al., 2012), but in this study Protein G Dynabeads (Life Technologies, 1004D) were used instead of Protein G Sepharose. The antibodies used are provided in Table S4. Immunoprecipitated DNAs or harvested total RNA was quantified by real-time PCR. The primer sequences for quantification are provided in Table S5.

### Protein Immunoprecipitation and Immunoblotting

HeLa or 293T cells were transfected with protein expression vectors and harvested 2 days after transfection. Cells were suspended in an extraction buffer (10 mM Tris [pH 7.4], 150 mM NaCl, 0.05 mM spermidine, 0.125 mM spermine, 0.5 mM EDTA, 0.5 mM DTT, and 0.1% digitonin) with a protease inhibitor cocktail (Sigma, P8340), incubated on ice for 5 min, and centrifuged at 16,100 × *g* for 5 min. The supernatant was harvested and used for immunoprecipitation with the Protein G Dynabeads and antibodies. SDS-PAGE was carried out using 4%–15% Mini-PROTEAN TGX precast gels (Bio-Rad), and proteins were transferred onto membrane. Membrane was blocked with 1% BSA and then used for immunoblotting. Antibodies used in immunoprecipitation and immunoblotting are shown in Table S4.

### Microscopy and Image Quantification

Cell images were acquired on an Axio Observer.Z1 (Zeiss) equipped with an LSM700 scanning module and an Objective Plan-Apochromat 63×/1.46 oil lens (Zeiss) using ZEN 2009 software (Zeiss). For image quantification, z-stack images were acquired with a spacing of 0.22 μm. Maximum-intensity projection of obtained slices was produced with the ZEN software, and ImageJ (NIH) software was used for image quantification.

## Author Contributions

J.O., N.S., K.O., N.M.C.M., and K.K. conducted experiments; H.K. and T.N. produced antibodies and DNA clones, respectively; J.O., V.L., W.C.E., and H.M. wrote the manuscript.

## Supplemental Information

Document S1. Figures S1–S7 and Tables S1–S5

Document S2. Article plus Supplemental Information
