# Supplementary material for: KAT7/HBO1/MYST2 Regulates CENP-A Chromatin Assembly by Antagonizing Suv39h1-Mediated Centromere Inactivation
Source: Dev Cell. 2016 Jun 6;37(5):413–27. doi: 10.1016/j.devcel.2016.05.006 (PMC4906249; doi:10.1016/j.devcel.2016.05.006)
Supplement: Document S1. Figures S1–S7 and Tables S1–S5 [file mmc1.pdf]

Developmental Cell, Volume *37*

Supplemental Information

KAT7/HBO1/MYST2 Regulates CENP-A Chromatin Assembly by Antagonizing Suv39h1-Mediated Centromere Inactivation

Jun-ichirou Ohzeki, Nobuaki Shono, Koichiro Otake, Nuno M.C. Martins, Kazuto Kugou, Hiroshi Kimura, Takahiro Nagase, Vladimir Larionov, William C. Earnshaw, and Hiroshi Masumoto

Supplemental Inventory

Supplemental Figure S1, Related to Figure 1.
Supplemental Figure S2, Related to Figure 2.
Supplemental Figure S3, Related to Figure 3.
Supplemental Figure S4, Related to Figure 4.
Supplemental Figure S5, Related to Figure 5.
Supplemental Figure S6, Related to Figure 6.
Supplemental Figure S7, Related to Figure 7.

Supplemental Figure legends

Supplemental Table S1, Related to Experimental Procedure.
Supplemental Table S2, Related to Experimental Procedure.
Supplemental Table S3, Related to Experimental Procedure.
Supplemental Table S4, Related to Experimental Procedure.
Supplemental Table S5, Related to Experimental Procedure.

Supplemental References

Figure S1

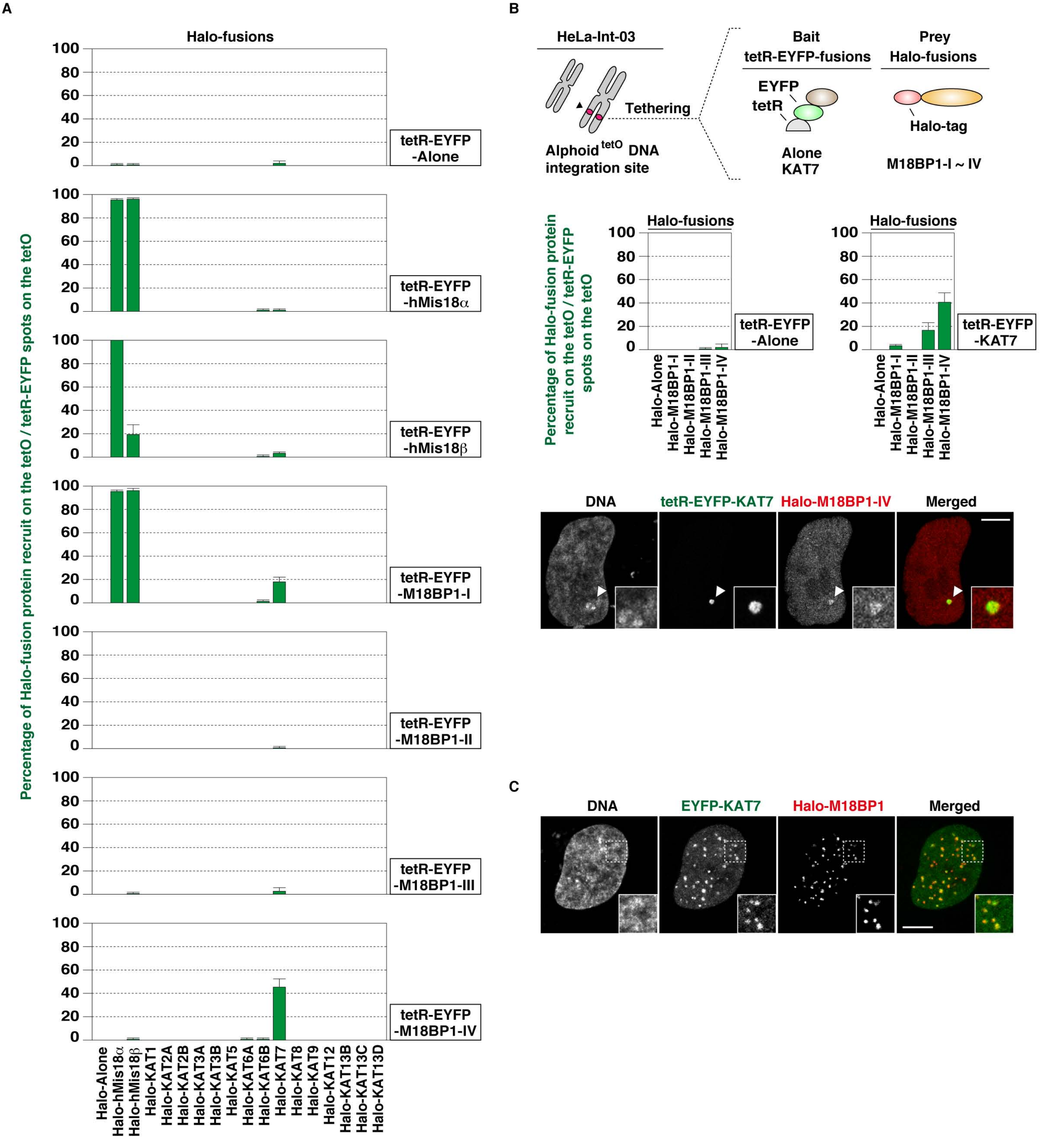
A
Halo-fusions
Percentage of Halo-fusion protein recruit on the tetO / tetR-EYFP spots on the tetO
tetR-EYFP -Alone
tetR-EYFP -hMis18α
tetR-EYFP -hMis18β
tetR-EYFP -M18BP1-I
tetR-EYFP -M18BP1-II
tetR-EYFP -M18BP1-III
tetR-EYFP -M18BP1-IV
100
80
60
40
20
0
Halo-Alone
Halo-hMis18α
Halo-hMis18β
Halo-KAT1
Halo-KAT2A
Halo-KAT2B
Halo-KAT3A
Halo-KAT3B
Halo-KAT5
Halo-KAT6A
Halo-KAT6B
Halo-KAT7
Halo-KAT8
Halo-KAT9
Halo-KAT12
Halo-KAT13B
Halo-KAT13C
Halo-KAT13D
B
HeLa-Int-03
Tethering
Alphoid tetO DNA integration site
Bait
tetR-EYFP-fusions
EYFP
tetR
Alone
KAT7
Prey
Halo-fusions
Halo-tag
M18BP1-I ~ IV
Percentage of Halo-fusion protein recruit on the tetO / tetR-EYFP spots on the tetO
Halo-fusions
tetR-EYFP -Alone
tetR-EYFP -KAT7
Halo-Alone
Halo-M18BP1-I
Halo-M18BP1-II
Halo-M18BP1-III
Halo-M18BP1-IV
DNA
tetR-EYFP-KAT7
Halo-M18BP1-IV
Merged
C
DNA
EYFP-KAT7
Halo-M18BP1
Merged

Figure S2

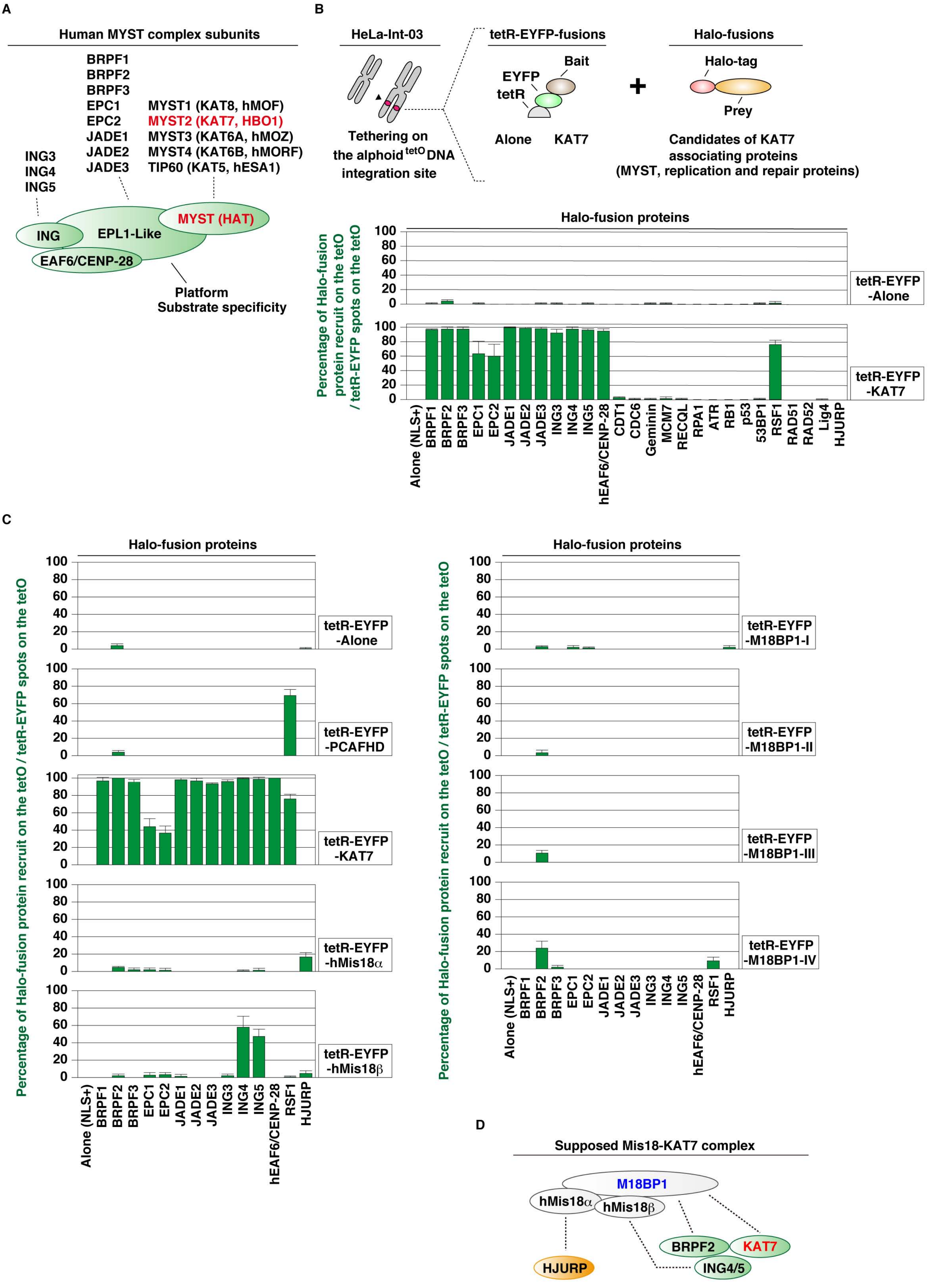
A
Human MYST complex subunits
BRPF1
BRPF2
BRPF3
EPC1
EPC2
JADE1
JADE2
JADE3
MYST1 (KAT8, hMOF)
MYST2 (KAT7, HBO1)
MYST3 (KAT6A, hMOZ)
MYST4 (KAT6B, hMORF)
TIP60 (KAT5, hESA1)
ING3
ING4
ING5
ING
EPL1-Like
MYST (HAT)
EAF6/CENP-28
Platform
Substrate specificity
B
HeLa-Int-03
Tethering on
the alphoid tetO DNA
integration site
tetR-EYFP-fusions
EYFP
tetR
Bait
Alone
KAT7
+
Halo-fusions
Halo-tag
Prey
Candidates of KAT7
associating proteins
(MYST, replication and repair proteins)
Halo-fusion proteins
Percentage of Halo-fusion
protein recruit on the tetO
/ tetR-EYFP spots on the tetO
100
80
60
40
20
0
tetR-EYFP
-Alone
tetR-EYFP
-KAT7
Alone (NLS+)
BRPF1
BRPF2
BRPF3
EPC1
EPC2
JADE1
JADE2
JADE3
ING3
ING4
ING5
hEAF6/CENP-28
CDT1
CDC6
Geminin
MCM7
RECQL
RPA1
ATR
RB1
p53
53BP1
RSF1
RAD51
RAD52
Lig4
HJURP
C
Halo-fusion proteins
Percentage of Halo-fusion protein recruit on the tetO / tetR-EYFP spots on the tetO
tetR-EYFP
-Alone
tetR-EYFP
-PCAFHD
tetR-EYFP
-KAT7
tetR-EYFP
-hMis18α
tetR-EYFP
-hMis18β
tetR-EYFP
-M18BP1-I
tetR-EYFP
-M18BP1-II
tetR-EYFP
-M18BP1-III
tetR-EYFP
-M18BP1-IV
Alone (NLS+)
BRPF1
BRPF2
BRPF3
EPC1
EPC2
JADE1
JADE2
JADE3
ING3
ING4
ING5
hEAF6/CENP-28
RSF1
HJURP
D
Supposed Mis18-KAT7 complex
M18BP1
hMis18α
hMis18β
HJURP
BRPF2
KAT7
ING4/5

Figure S3

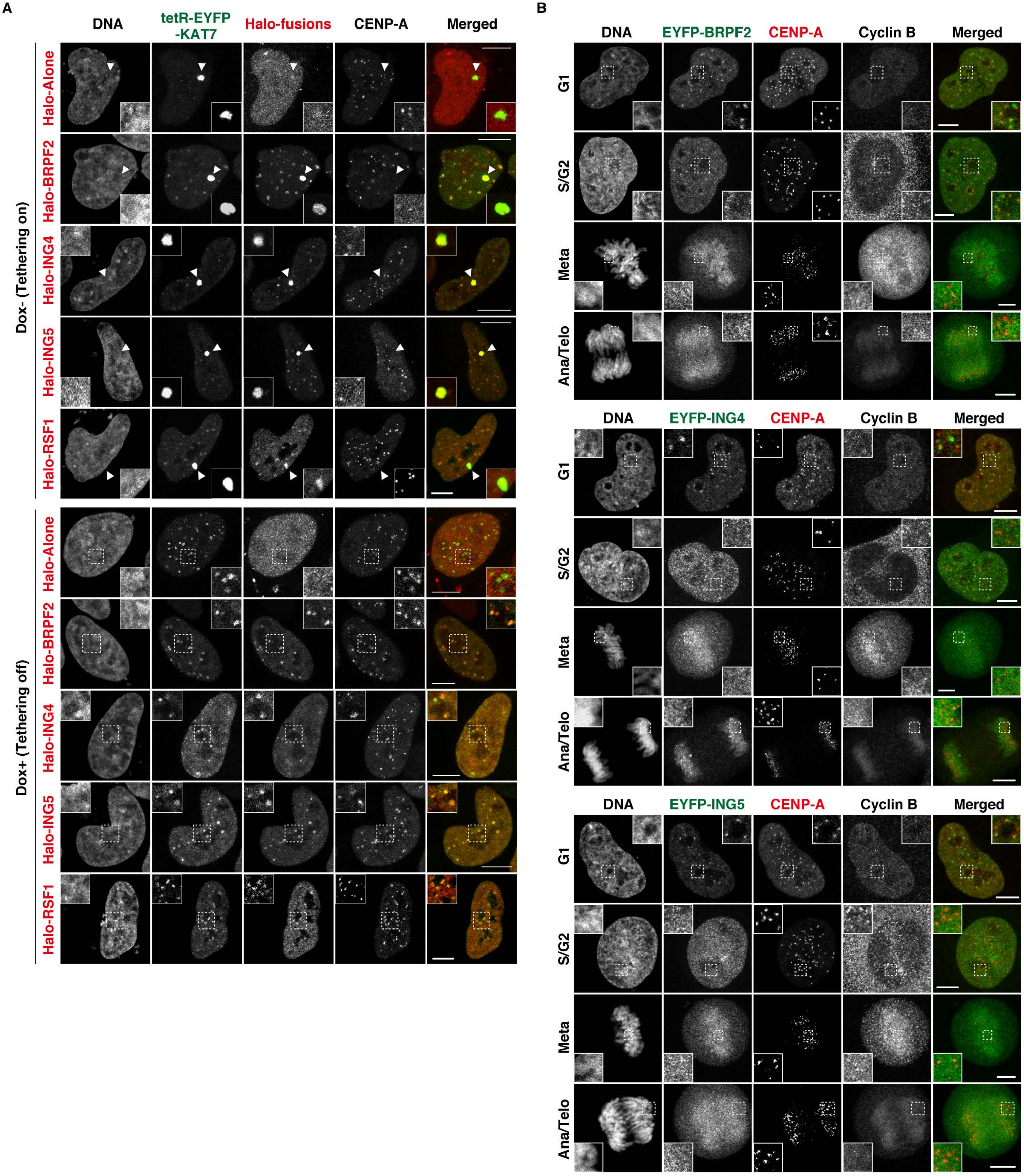

A
DNA
tetR-EYFP -KAT7
Halo-fusions
CENP-A
Merged
Dox- (Tethering on)
Halo-Alone
Halo-BRPF2
Halo-ING4
Halo-ING5
Halo-RSF1
Dox+ (Tethering off)
Halo-Alone
Halo-BRPF2
Halo-ING4
Halo-ING5
Halo-RSF1
B
DNA
EYFP-BRPF2
CENP-A
Cyclin B
Merged
G1
S/G2
Meta
Ana/Telo
DNA
EYFP-ING4
CENP-A
Cyclin B
Merged
G1
S/G2
Meta
Ana/Telo
DNA
EYFP-ING5
CENP-A
Cyclin B
Merged
G1
S/G2
Meta
Ana/Telo

Figure S4

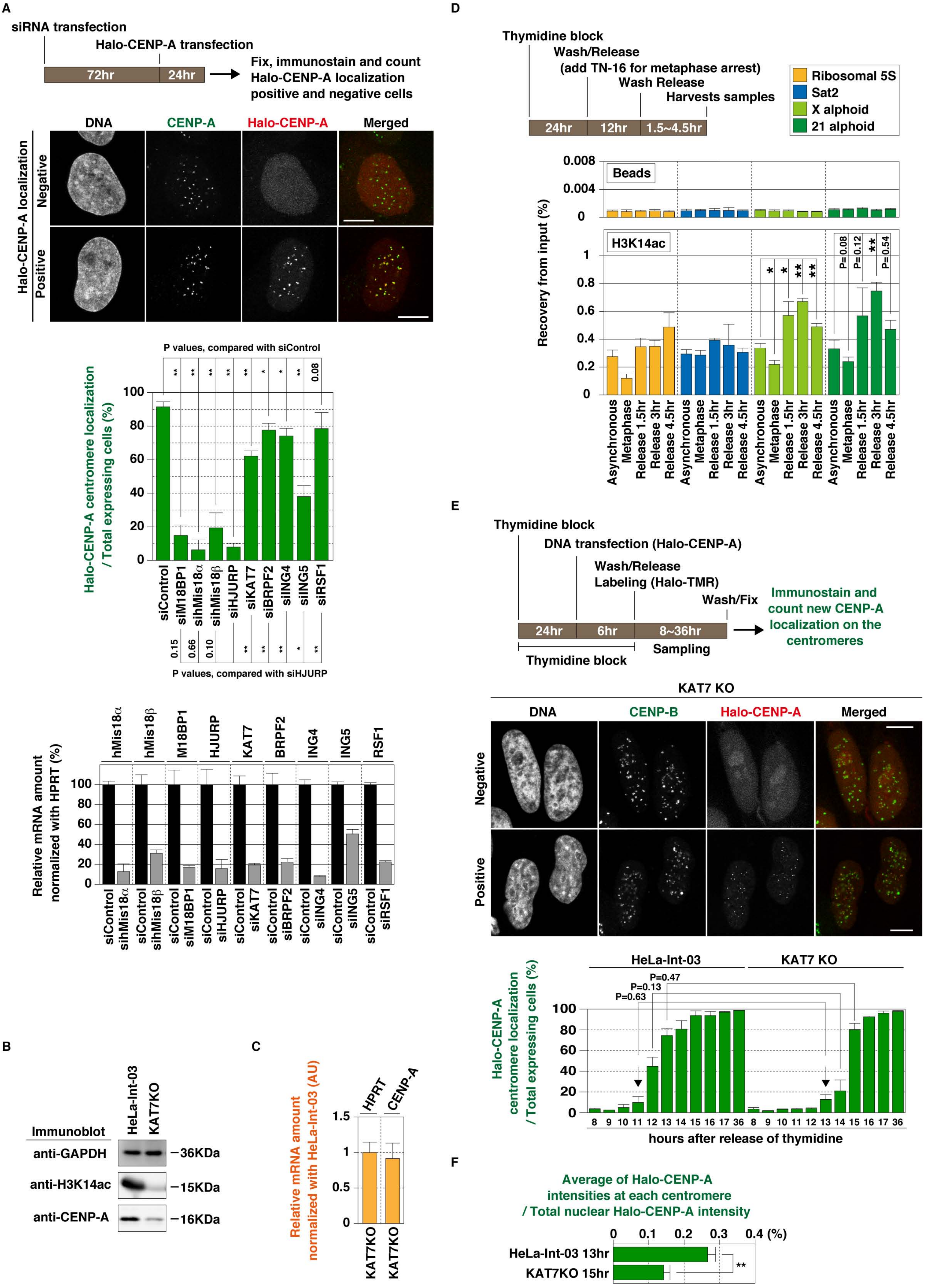

A
siRNA transfection
Halo-CENP-A transfection
72hr
24hr
Fix, immunostain and count Halo-CENP-A localization positive and negative cells
DNA
CENP-A
Halo-CENP-A
Merged
Halo-CENP-A localization
Negative
Positive
P values, compared with siControl
Halo-CENP-A centromere localization / Total expressing cells (%)
siControl
siM18BP1
sihMis18α
sihMis18β
siHJURP
siKAT7
siBRPF2
siING4
siING5
siRSF1
P values, compared with siHJURP
Relative mRNA amount normalized with HPRT (%)
hMis18α
hMis18β
M18BP1
HJURP
KAT7
BRPF2
ING4
ING5
RSF1
B
Immunoblot
HeLa-Int-03
KAT7KO
anti-GAPDH
anti-H3K14ac
anti-CENP-A
–36KDa
–15KDa
–16KDa
C
Relative mRNA amount normalized with HeLa-Int-03 (AU)
HPRT
CENP-A
KAT7KO
D
Thymidine block
Wash/Release
(add TN-16 for metaphase arrest)
Wash Release
Harvests samples
24hr
12hr
1.5~4.5hr
Ribosomal 5S
Sat2
X alphoid
21 alphoid
Beads
H3K14ac
Recovery from input (%)
P=0.08
P=0.12
P=0.54
Asynchronous
Metaphase
Release 1.5hr
Release 3hr
Release 4.5hr
E
Thymidine block
DNA transfection (Halo-CENP-A)
Wash/Release
Labeling (Halo-TMR)
Wash/Fix
24hr
6hr
8~36hr
Sampling
Immunostain and count new CENP-A localization on the centromeres
KAT7 KO
DNA
CENP-B
Halo-CENP-A
Merged
Negative
Positive
HeLa-Int-03
KAT7 KO
P=0.47
P=0.13
P=0.63
Halo-CENP-A centromere localization / Total expressing cells (%)
hours after release of thymidine
F
Average of Halo-CENP-A intensities at each centromere / Total nuclear Halo-CENP-A intensity
0 0.1 0.2 0.3 0.4 (%)
HeLa-Int-03 13hr
KAT7KO 15hr

Figure S5

A

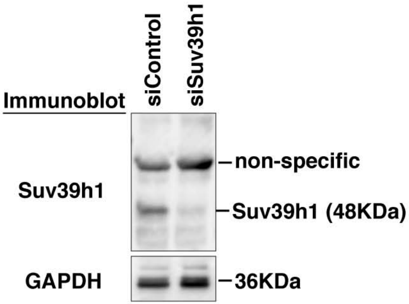

Immunoblot
siControl
siSuv39h1
Suv39h1
non-specific
Suv39h1 (48KDa)
GAPDH
36KDa

B

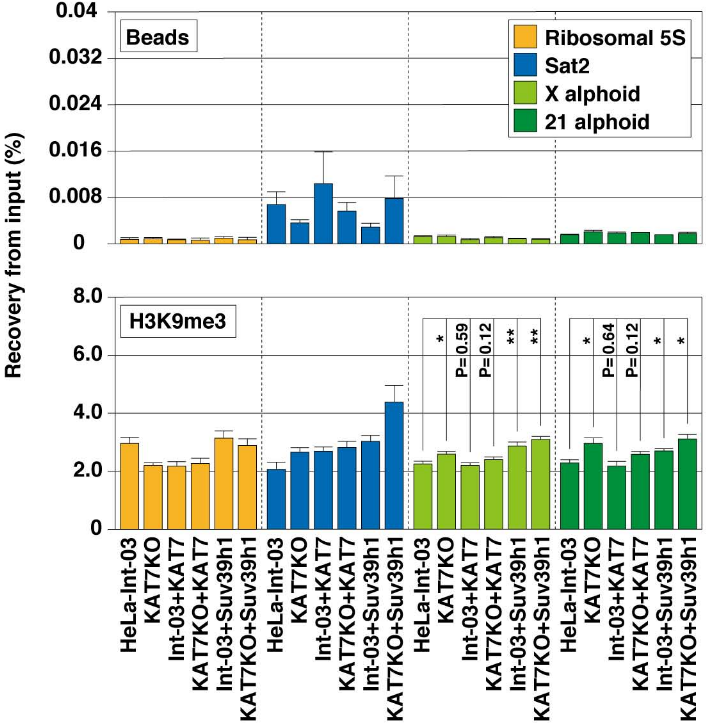

Recovery from input (%)
Beads
H3K9me3
Ribosomal 5S
Sat2
X alphoid
21 alphoid
0.04
0.032
0.024
0.016
0.008
0
8.0
6.0
4.0
2.0
0
P=0.59
P=0.12
P=0.64
P=0.12
HeLa-Int-03
KAT7KO
Int-03+KAT7
KAT7KO+KAT7
Int-03+Suv39h1
KAT7KO+Suv39h1

Figure S6

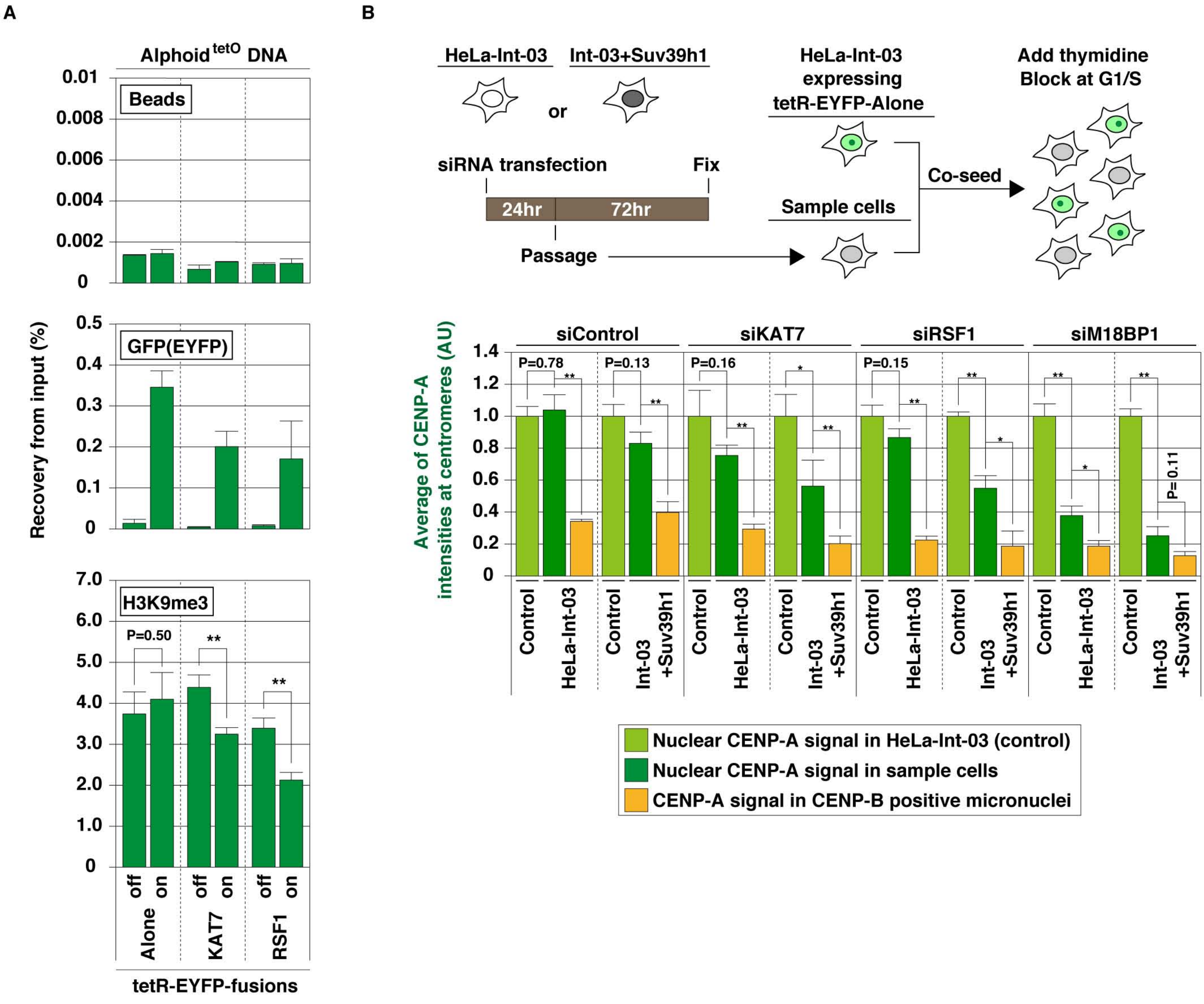
A
Alphoid tetO DNA
Beads
0.01
0.008
0.006
0.004
0.002
0
GFP(EYFP)
0.5
0.4
0.3
0.2
0.1
0
Recovery from input (%)
H3K9me3
7.0
6.0
5.0
4.0
3.0
2.0
1.0
0
P=0.50
**
**
off
on
off
on
off
on
Alone
KAT7
RSF1
tetR-EYFP-fusions
B
HeLa-Int-03
Int-03+Suv39h1
or
siRNA transfection
Fix
24hr
72hr
Passage
HeLa-Int-03 expressing tetR-EYFP-Alone
Sample cells
Co-seed
Add thymidine Block at G1/S
Average of CENP-A intensities at centromeres (AU)
siControl
siKAT7
siRSF1
siM18BP1
1.4
1.2
1.0
0.8
0.6
0.4
0.2
0
P=0.78
P=0.13
P=0.16
P=0.15
P= 0.11
**
*
Control
HeLa-Int-03
Int-03 +Suv39h1
Nuclear CENP-A signal in HeLa-Int-03 (control)
Nuclear CENP-A signal in sample cells
CENP-A signal in CENP-B positive micronuclei

Figure S7

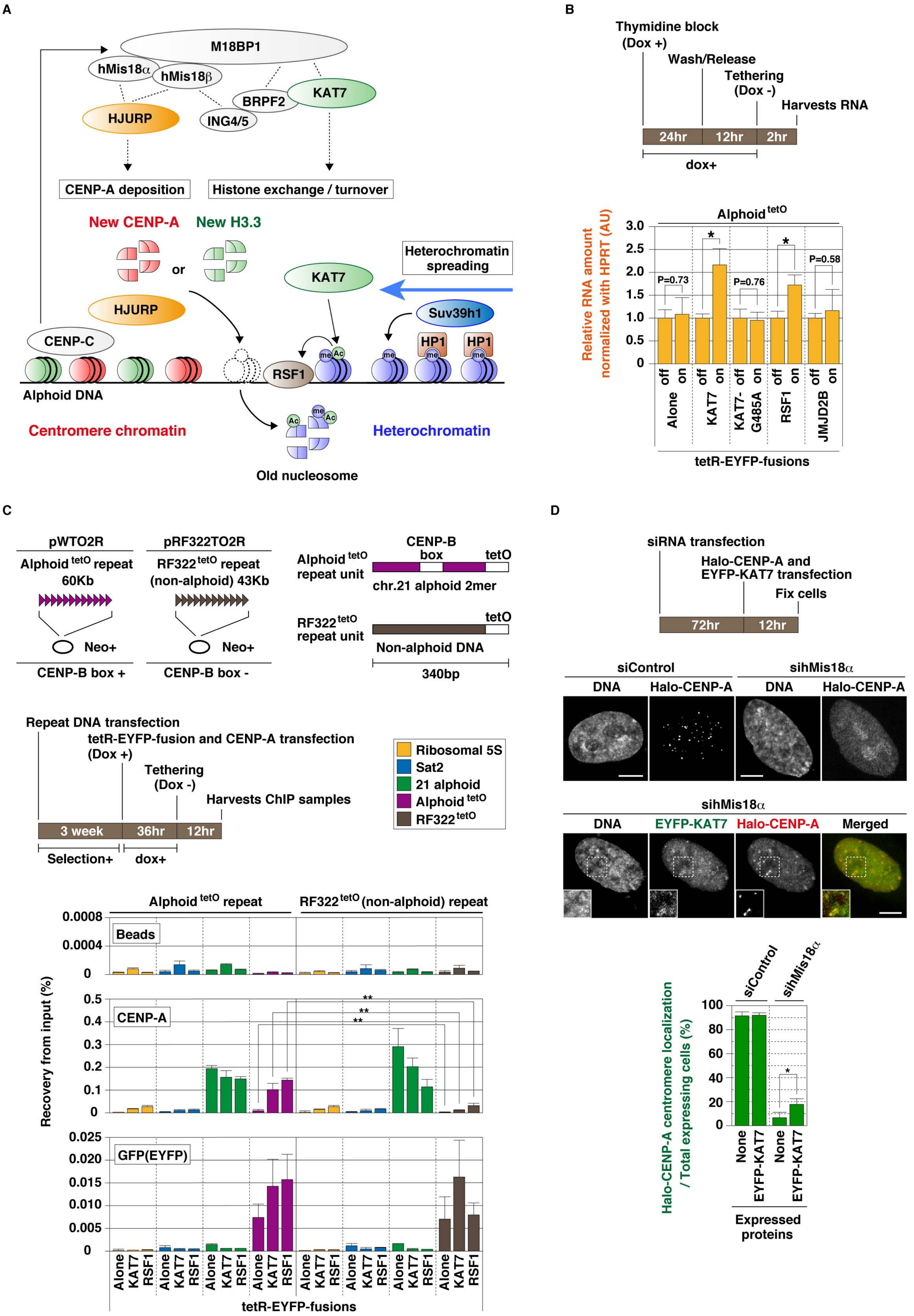
A
M18BP1
hMis18α
hMis18β
BRPF2
KAT7
HJURP
ING4/5
CENP-A deposition
Histone exchange / turnover
New CENP-A
New H3.3
or
Heterochromatin spreading
KAT7
HJURP
Suv39h1
CENP-C
HP1
HP1
me
Ac
RSF1
Alphoid DNA
Centromere chromatin
Heterochromatin
Old nucleosome
B
Thymidine block (Dox +)
Wash/Release
Tethering (Dox -)
Harvests RNA
24hr
12hr
2hr
dox+
Alphoid tetO
Relative RNA amount normalized with HPRT (AU)
3.0
2.5
2.0
1.5
1.0
0.5
0
P=0.73
P=0.76
P=0.58
*
off
on
Alone
KAT7
KAT7-G485A
RSF1
JMJD2B
tetR-EYFP-fusions
C
pWTO2R
Alphoid tetO repeat 60Kb
pRF322TO2R
RF322 tetO repeat (non-alphoid) 43Kb
Neo+
CENP-B box +
CENP-B box -
Alphoid tetO repeat unit
CENP-B box
tetO
chr.21 alphoid 2mer
RF322 tetO repeat unit
Non-alphoid DNA
340bp
Repeat DNA transfection
tetR-EYFP-fusion and CENP-A transfection (Dox +)
Tethering (Dox -)
Harvests ChIP samples
3 week
36hr
12hr
Selection+
dox+
Ribosomal 5S
Sat2
21 alphoid
Alphoid tetO
RF322 tetO
Alphoid tetO repeat
RF322 tetO (non-alphoid) repeat
Beads
0.0008
0.0004
0
CENP-A
0.5
0.4
0.3
0.2
0.1
0
**
GFP(EYFP)
0.025
0.020
0.015
0.010
0.005
0
Recovery from input (%)
Alone
KAT7
RSF1
tetR-EYFP-fusions
D
siRNA transfection
Halo-CENP-A and EYFP-KAT7 transfection
Fix cells
72hr
12hr
siControl
sihMis18α
DNA
Halo-CENP-A
EYFP-KAT7
Merged
Halo-CENP-A centromere localization / Total expressing cells (%)
100
80
60
40
20
0
*
None
EYFP-KAT7
Expressed proteins

Supplemental Figure Legends

Figure S1. Supplemental information for Figure 1, Related to Figure 1

(A) Graphical representation of the data in Figure 1C. Data are presented as mean + s.d. (n=3). (B) M18BP1 recruitment with KAT7 tethering. (Top) Schema for the experiments. (Middle) The observed frequency of recruitment of Halo-fusion proteins by tetR-EYFP-fusion protein tethering (% of cells that have a detectable signal) was plotted (>50 tetR-EYFP spots were counted in each sample). Data are presented as mean + s.d. (n=3). (Bottom) Fluorescence images were obtained by DAPI staining (DNA), EYFP (green), and Halo-TMR-ligand (red). White arrowheads indicate the loci of tetR-EYFP-fusion protein spots on the alphoid^{tetO} DNA integration site. (C) Co-localization of EYFP-KAT7 and Halo-M18BP1. Fluorescence images were obtained by DAPI staining (DNA), EYFP (green), and Halo-TMR-ligand (red). Scale bars = 5 μm.

Figure S2. Screening of KAT7-associating proteins, Related to Figure 2

(A) Scheme of the human MYST complexes formed on the EPL1-like motif-containing platform proteins. The MYST domain-containing HAT proteins interact with this EPL1-like motif. Inhibitor of growth (ING) subunits and EAF6/CENP-28 proteins are also components of the MYST complexes (Doyon et al., 2006; Ohta et al., 2010; Avvakumov et al., 2012). Most of the known MYST complexes are heterotetramers of the MYST domain HAT, ING subunit, EAF6/CENP-28, and EPL1-like platform proteins. In addition, KAT7 and BRPF2 heterodimer has been reported (Mishima et al., 2011). (B) Scheme of screening KAT7-associating proteins. TetR-EYFP-Alone or -KAT7 proteins were tethered to the alphoid^{tetO} DNA integration site as "bait" and recruitment of Halo-tag-fused "prey" proteins tested. The Halo-fused genes were related to known

KAT7 functions, DNA replication, repair, and putative KAT7-interacting proteins. The frequency of recruitment of Halo-fusion proteins by tetR-EYFP-fusion protein tethering (% of cells that have a detectable signal) was plotted. Data are presented as mean + s.d. (n≥2). (C) A graphical representation of the data in Figure 2A heat map. Data are presented as mean + s.d. (n≥2). (D) A conceptual diagram of a hypothetical Mis18-KAT7 protein complex assumed from the results in Figure 2A.

Figure S3. Localizations of Mis18-KAT7 relating proteins, Related to Figure 3

(A) Localizations of tetR-EYFP-KAT7 and Halo-fused proteins in the presence or absence of doxycycline. Fluorescence images were obtained with DAPI (DNA), EYFP (green) and Halo-TMR-ligand (red). White arrowheads indicate the presence of tetR-EYFP-fusion protein spots on the alphoid^{tetO} DNA integration site. (B) Localization of EYFP-BRPF2, -ING4 and -ING5 during the cell cycle. Fluorescence images were obtained with DAPI (DNA), EYFP (green), anti-CENP-A antibody (red), and anti-cyclin B antibody (far red: shown in gray). Cell cycles were distinguished by images of DAPI and anti-cyclin B staining. Scale bars = 5 μm. See also Figure 3A.

Figure S4. Supplemental information for Figure 4, Related to Figure 4

(A) Halo-CENP-A localization at the centromere following depletion of Mis18 and KAT7-related proteins. (Top) Scheme of the depletion experiment and examples of Halo-CENP-A localization (siING5). Fluorescence images were obtained with DAPI (DNA), anti-CENP-A antibody (green), and Halo-TMR-Ligand (red). (Middle) The centromere localization frequency of Halo-CENP-A is plotted (>200 cells were counted in each sample). Data are presented as mean + s.d. (n=3). *P<0.05, **P<0.005 (t-test). (Bottom) Relative mRNA levels. Cells were transfected with siRNA and total RNA was

purified after 3 days. The total RNA was reverse transcribed and quantitated by qPCR. Quantitated target mRNA amounts were normalized to *HPRT* mRNA and plotted. The primer sets used are indicated at the top of the graph. The transfected siRNAs are indicated at the bottom of the graph. Data are presented as mean + s.d. (n=3). (B) H3K14ac and CENP-A immunoblotting using total cell lysate. GAPDH was used as a loading control. (C) RT-PCR quantitation of mRNA levels. Data are presented as mean + s.d. (n=3). (D) Time course ChIP analysis. Cells were harvested at each time point and fixed for ChIP analysis. ChIP was carried out with anti-H3K14ac antibody or beads alone (no antibody) as a control. Data are presented as mean + s.d. (n=3). *P<0.05, **P<0.005 (t-test). (E) (Top) Scheme of detecting a new CENP-A localization at the centromere. Cells were synchronized at G1/S using thymidine and then transfected with the vector expressing Halo-CENP-A. Cells were fixed at each time point after release of the thymidine block. (Middle) Fluorescence images were obtained with DAPI (DNA), anti-CENP-B antibody (green), and Halo-TMR-ligand (red). Scale bars = 5 μm. (Bottom) Centromere localization frequency of Halo-CENP-A (>200 cells were counted in each sample). Black arrows indicate a time point when >50% of cells exit mitosis. Data are presented as mean + s.d. (n=2). (F) Average of Halo-CENP-A intensity at each centromere. From the panel E experiments, cell samples from a time point when >50% of cells had new CENP-A localization (i.e. HeLa-Int-03 13hr and KAT7KO 15hr) were selected, and used for image quantitation. Total nuclear Halo-CENP-A intensity and centromere-localized Halo-CENP-A intensities were quantitated. The average Halo-CENP-A intensity at each centromere was normalized to the total nuclear Halo-CENP-A intensity of each cell. Data are presented as mean + s.e. (n≥10 cells, >20 centromeres were analyzed in each cell). **P<0.005 (t-test).

Figure S5. Supplemental information for Figure 5, Related to Figure 5

(A) Suv39h1 immunoblotting. Total cell lysates of control and Suv39h1 depleted cells were analyzed. See also Figure 5B. (B) ChIP analysis. ChIP was carried out with anti-H3K9me3 antibody or beads alone (no antibody) as a control. Data are presented as mean + s.e. ($n \geq 3$). *$P<0.05$, **$P<0.005$ (t-test).

Figure S6. Supplemental information for Figure 6, Related to Figure 6

(A) Confirmation of H3K9me3 reduction by ChIP. ChIP was carried out with anti-GFP antibody, anti-H3K9me3 antibody or beads alone (no antibody) as a control. Data are presented as mean + s.d. ($n=3$). **$P<0.005$ (t-test). (B) CENP-A intensity quantitation of siRNA transfected cells in Figure 6E. Control (HeLa-Int-03 expressing tetR-EYFP) and target cells were co-seeded on coverslips, and arrested at G1/S with thymidine. The cells were fixed, stained with CENP-A and CENP-B antibody and then used for microscopy. The CENP-A signal at centromeres including CENP-B positive micronuclei was quantitated and plotted in the right panel. Data are presented as mean + s.e. ($n \geq 5$ micronuclei). *$P<0.05$, **$P<0.005$ (t-test). See also Figure 6E.

Figure S7. A models and Supplemental information about KAT7 functions, Related to Figure 7

(A) Models of KAT7 function. KAT7 acetylates not only histone H3 but also H4 (Miotto et al., 2010). CENP-C interacts with HJURP, and may have an additional essential role not shared by the Mis18-KAT7 complex in the *de novo* endogenous CENP-A assembly pathway (Zasadzińska et al., 2013; Müller et al., 2014; Tachiwana et al., 2015; Shono et al., 2015). See also the Discussion. (B) Quantitation of alphoid^{tetO} RNA levels. (Top) Schema of time course. TetR-EYFP-fusion protein-expressing cells were synchronized

at G1/S with thymidine. Cells were then released and cultured for 12 hour (at this time point, most of cells were in G1 phase) in the presence of doxycycline. Doxycycline was washed out 2 hour before RNA harvesting. The amount of RNA was quantified by quantitative RT-PCR. (Bottom) Alphoid^{tetO} RNA levels were normalized with *HPRT* and plotted in the graph. Data are presented as mean + s.d. (n=3). *P<0.05 (t-test). (C) ChIP analysis combined with transient transfection using a non-alphoid DNA sequence. (Top) Plasmids used for this study. Plasmid pWTO2R contains 60kb of alphoid^{tetO} repeat DNA insert (Ohzeki et al., 2012). Plasmid pRF322TO2R contains a synthetic repeat of pBR322 restriction fragment (Ohzeki et al., 2002) combined with the tetO site. These plasmids contain a Neomycin resistant gene cassette. Plasmid pWTO2R or pRF322TO2R, was respectively transfected into HeLa cells and selected with Geneticin (selective drug for Neomycin) for 3 weeks. Next, vectors expressing tetR-EYFP-fusion proteins and Halo-CENP-A were transfected to the cells. After 12 hours of tethering, cells were harvested for ChIP. (Bottom) ChIP analysis. ChIP was carried out with anti-CENP-A antibody, anti-GFP antibody or beads alone (no antibody) as a control. Data are presented as mean + s.d. (n=3). **P<0.005 (t-test). (D) KAT7 overproduction bypasses the absence of hMis18α. (Top) Scheme for hMis18α depletion and KAT7 overproduction. (Middle) Examples of staining. Fluorescence images were obtained by DAPI staining (DNA), EYFP (green), and Halo-TMR-ligand (red). Scale bars = 5 μm. (Bottom) Data are presented as mean + s.d. (n=3, >200 cells were counted in each sample). *P<0.05 (t-test).

Table S1. siRNAs Used in this Study, Related to Experimental Procedure

Target gene	Sequence	Reference
Control (siNegative)	No information	Ambion, AM4611
hMis18α	5'-CAGAAGCUAUCCAAACGUGTT-3'	Fujita et al., 2007
hMis18β	5'-CAAUCGCUUAAAAUCACUAtt-3'	Ambion, s22367
M18BP1	5'-GAAGUCUGGUGUUAGGAAATT-3'	Fujita et al., 2007
HJURP	5'-CUACUGGGCUCAACUGCAAUU-3'	Dunleavy et al., 2009
KAT7	5'-GGAUGCCCACUGUAUCAUAtt-3'	Ambion, s253
BRPF2	5'-GAAACUAUAGACAAGUUAAtt-3'	Ambion, s24388
ING4	5'-UGCUCGUGCUCGUUCCAAAtt-3'	Ambion, s27553
ING5	5'-CCUUACCACGAAACCCAAAtt-3'	Ambion, s38829
RSF1	5'-GGAUAUCAAGUCGGAAAAAtt-3'	Ambion, s28648
Suv39h1	5'-AGAACAGCUUCGUCAUGGAtt-3'	Ambion, s13658

Table S2. Plasmid Vectors Used in this Study, Related to Experimental Procedure

Name	Backbone	Cloned genes	Reference
pJETY3		tetR-EYFP-alone	Ohzeki et al., 2012
pJETY3-hMis18α	pJETY3	tetR-EYFP-hMis18α	Ohzeki et al., 2012
pJETY3-hMis18β	pJETY3	tetR-EYFP-hMis18β	This study
pJETY3-M18BP1-I	pJETY3	tetR-EYFP-M18BP1-I	This study
pJETY3-M18BP1-II	pJETY3	tetR-EYFP-M18BP1-II	This study
pJETY3-M18BP1-III	pJETY3	tetR-EYFP-M18BP1-III	This study
pJETY3-M18BP1-IV	pJETY3	tetR-EYFP-M18BP1-IV	This study
pJETY3-KAT7	pJETY3	tetR-EYFP-KAT7	This study
pJETY3-KAT7 G485A	pJETY3	tetR-EYFP-KAT7 G485A	This study
pJETY3-PCAFHD	pJETY3	tetR-EYFP-PCAFHD	Ohzeki et al., 2012
pJETY3-BRPF2	pJETY3	tetR-EYFP-BRPF2	This study
pJETY3-ING4	pJETY3	tetR-EYFP-ING4	This study
pJETY3-ING5	pJETY3	tetR-EYFP-ING5	This study
pJETY3-RSF1	pJETY3	tetR-EYFP-RSF1	This study
pJETY3-JMJD2B	pJETY3	tetR-EYFP-JMJD2B	This study
pJETB3		tetR-BFP-alone	This study
pJETB3-hMis18α	pJETB3	tetR-BFP-hMis18α	This study
pJETB3-hMis18β	pJETB3	tetR-BFP-hMis18β	This study
pJ3-EYFP		EYFP	This study
pJ3-EYFP-KAT7	pJ3-EYFP	EYFP-KAT7	This study
pJ3-EYFP-BRPF2	pJ3-EYFP	EYFP-BRPF2	This study
pJ3-EYFP-ING4	pJ3-EYFP	EYFP-ING4	This study
pJ3-EYFP-ING5	pJ3-EYFP	EYFP-ING5	This study
pJ4IB-3HA-M18BP1-I	pJ4IB-3HA	3xHA-M18BP1-I	This study
pJ4IB-3HA-M18BP1-IV	pJ4IB-3HA	3xHA-M18BP1-IV	This study
pJ4IB-3HA-hMis18β	pJ4IB-3HA	3xHA-hMis18β	This study
pJ4IB-3HA-ING4	pJ4IB-3HA	3xHA-ING4	This study
pJ4IB-3FL-KAT7	pJ4IB-3FL	3xFLAG-KAT7	This study
pJ4IB-3FL-hMis18α	pJ4IB-3FL	3xFLAG-hMis18α	This study
pJ4IB-3FL-ING5	pJ4IB-3FL	3xFLAG-ING5	This study
pJ4-Halo7		Halo-tag	
pJ4IB-Halo7-Suv39h1	pJ4-Halo7	Halo-Suv39h1	This study
pJ4IB-Halo7-M18BP1-I	pJ4-Halo7	Halo-M18BP1-I	This study
pJ4IB-Halo7-M18BP1-II	pJ4-Halo7	Halo-M18BP1-II	This study
pJ4IB-Halo7-M18BP1-III	pJ4-Halo7	Halo-M18BP1-III	This study
pJ4IB-Halo7-M18BP1-IV	pJ4-Halo7	Halo-M18BP1-IV	This study
pJTI PhiC31 Int		PhiC31 integrase	Life Technologies, A10894
pX260		Cas9, gRNA	Cong et al., 2013
pJ4IB-Cas9		Cas9	This study
pU6CR-KAT7		gRNA for KAT7 KO	This study
pWTO2R	pBAC108L	tetO alphoid DNA	Ohzeki et al., 2012
pRF322TO2R	pBAC108L	synthetic repeat DNA	This study

Name	Backbone	Cloned genes	Reference
pFN21A		Halo-tag	Promega, G2821
pFN21A-Halo-NLS	pFN21A	Halo-NLS	This study
pFN21A-Halo-hMis18α	pFN21A	Halo-hMis18α	FHC27152
pFN21A-Halo-hMis18β	pFN21A	Halo-hMis18β	FHC25353
pFN21A-Halo-M18BP1	pFN21A	Halo-M18BP1	FHC11044
pFN21A-Halo-KAT1	pFN21A	Halo-KAT1	FHC21224
pFN21A-Halo-KAT2A	pFN21A	Halo-KAT2A	FHC03762
pFN21A-Halo-KAT2B	pFN21A	Halo-KAT2B	FHC11610
pFN21A-Halo-KAT3A	pFN21A	Halo-KAT3A	FHC11778
pFN21A-Halo-KAT3B	pFN21A	Halo-KAT3B	FHC01787
pFN21A-Halo-KAT5	pFN21A	Halo-KAT5	FHC02643
pFN21A-Halo-KAT6A	pFN21A	Halo-KAT6A	FHC03163
pFN21A-Halo-KAT6B	pFN21A	Halo-KAT6B	FHC00523
pFN21A-Halo-KAT7	pFN21A	Halo-KAT7	FHC06090
pFN21A-Halo-KAT8	pFN21A	Halo-KAT8	FHC02573
pFN21A-Halo-KAT9	pFN21A	Halo-KAT9	FHC02650
pFN21A-Halo-KAT12	pFN21A	Halo-KAT12	FHC11853
pFN21A-Halo-KAT13B	pFN21A	Halo-KAT13B	FHC01529
pFN21A-Halo-KAT13C	pFN21A	Halo-KAT13C	FHC01199
pFN21A-Halo-KAT13D	pFN21A	Halo-KAT13D	FHC00509
pFN21A-Halo-Suv39h1	pFN21A	Halo-Suv39h1	FHC09879
pFN21A-Halo-BRPF1	pFN21A	Halo-BRPF1	FHC01228
pFN21A-Halo-BRPF2	pFN21A	Halo-BRPF2	FHC02120
pFN21A-Halo-BRPF3	pFN21A	Halo-BRPF3	FHC01683
pFN21A-Halo-EPC1	pFN21A	Halo-EPC1	FHC11123
pFN21A-Halo-EPC2	pFN21A	Halo-EPC2	FHC30908
pFN21A-Halo-JADE1	pFN21A	Halo-JADE1	FHC09414
pFN21A-Halo-JADE2	pFN21A	Halo-JADE2	FHC00474
pFN21A-Halo-JADE3	pFN21A	Halo-JADE3	FHC00463
pFN21A-Halo-ING3	pFN21A	Halo-ING3	FHC02414
pFN21A-Halo-ING4	pFN21A	Halo-ING4	FHC02495
pFN21A-Halo-ING5	pFN21A	Halo-ING5	FHC03674
pFN21A-Halo-CENP-28	pFN21A	Halo-CENP-28	FHC04343
pFN21A-Halo-RSF1	pFN21A	Halo-RSF1	FHC11761
pFN21A-Halo-HJURP	pFN21A	Halo-HJURP	FHC28790
pFN21A-Halo-CENP-A	pFN21A	Halo-CENP-A	FHC20935
pFN21A-Halo-hisone H3.3	pFN21A	Halo-hisone H3.3	This study
pFN21A-Halo-CDT1	pFN21A	Halo-CDT1	This study
pFN21A-Halo-CDC6	pFN21A	Halo-CDC6	FHC10183
pFN21A-Halo-Geminin	pFN21A	Halo-Geminin	FHC10644
pFN21A-Halo-MCM7	pFN21A	Halo-MCM7	FHC05950
pFN21A-Halo-RPA1	pFN21A	Halo-RPA1	FHC01462
pFN21A-Halo-ATR	pFN21A	Halo-ATR	FHC11858
pFN21A-Halo-RB1	pFN21A	Halo-RB1	FHC01227
pFN21A-Halo-p53	pFN21A	Halo-p53	FHC10374
pFN21A-Halo-53BP1	pFN21A	Halo-53BP1	FHC00998
pFN21A-Halo-RAD51	pFN21A	Halo-RAD51	FHC10574
pFN21A-Halo-RAD52	pFN21A	Halo-RAD52	FHC10450
pFN21A-Halo-Lig4	pFN21A	Halo-Lig4	FHC10347

Note; Halo-tag fused genes are available at Kazusa DNA Research Institute Human cDNA/ORF Clone distribution (http://www.kazusa.or.jp/kop/dsearch-e/).

Table S3. Cell Lines Used in this Study, Related to Experimental Procedure

Name	Host cell	Transfected alphoid DNA	Event	Modified genes	Expressing genes	Reference
HeLa-Int-03	HeLa	pWTO2R (Chr. 21-based tetO-Alphoid DNA repeat)	Integration	None	None	Ohzeki et al., 2012
Int-03+KAT7	HeLa	pWTO2R	Integration	None	EYFP-KAT7	This study
Int-03+Suv39h1	HeLa	pWTO2R	Integration	None	Halo-Suv39h1	This study
Int-03+tR-alone	HeLa	pWTO2R	Integration	None	tetR-EYFP-alone	This study
KAT7KO	HeLa	pWTO2R	Integration	KAT7 knockout	None	This study
KAT7KO+KAT7	HeLa	pWTO2R	Integration	KAT7 knockout	EYFP-KAT7	This study
KAT7KO+Suv39h1	HeLa	pWTO2R	Integration	KAT7 knockout	Halo-Suv39h1	This study

Table S4. Antibodies Used in this Study, Related to Experimental Procedure

Antibodies	Catalog number	Antibody produced in	Usage	Reference
anti-CENP-A (A1)	None	Mouse	Immunostain, Immunoblot, ChIP	Ohzeki et al., 2002
anti-CENP-A (6F2)	None	Rat	Immunostain	Kind gift from Kinya Yoda
anti-CENP-B (5E6C1)	None	Mouse	Immunostain	Ohzeki et al., 2002
anti-KAT7	abcam, ab70183	Rabbit	Immunostain, Immunoblot, IP	
anti-Suv39h1	SIGMA, S8316	Mouse	Immunoblot	
anti-M18BP1	NOVUS, NBP1-47290	Rabbit	Immunoblot, IP	
anti-HJURP (27D1)	None	Mouse	Immunoblot	This study
anti-RSF1 (EPR3749)	GeneTex, GTX62703	Rabbit	Immunoblot	
anti-H3K14ac (7G8)	None	Mouse	Immunostain, Immunoblot, ChIP	This study
anti-H3K9me3	WAKO, MABI 0308	Mouse	ChIP	
anti-H3K9me3	abcam, ab8898	Rabbit	Immunostain	
anti-Cyclin B	SIGMA, C-8831	Rabbit	Immunostain	
anti-GFP	MBL, 598	Rabbit	Immunoblot	
anti-Halo-tag	Promega, G928A	Rabbit	Immunoblot	
anti-HA-7	SIGMA, H3663	Mouse	Immunoblot	
anti-FLAGM2	SIGMA, F3165	Mouse	Immunoblot	
anti-tubulin	abcam, ab6160	Rat	Immunostain	

Table S5. Primer DNA Sequences Used for Quantitative PCR, Related to Experimental Procedure

Name	primer 1 (5' to 3')	primer 2 (5' to 3')	Reference
5S Ribosomal DNA	ACGCTGGGTTCCCTGCCGTT	TGGCTGGCGTCTGTGGCACCCGCT	Ohzeki et al., 2012
Satellite 2	TCGCATAGAATCGAATGGAA	GCA TTCGAGTCCGTGGA	Ohzeki et al., 2012
X alphoid	AGA TTTGGACCGCTTTGAGGC	CCGTTCAGTTATGGGAAGTTGA	Ohzeki et al., 2012
21-I alphoid	CTAGACAGAAGCCCTCTCAG	GGGAAGACA TTCCCTTTTTCACC	Ohzeki et al., 2012
tetO alphoid	GTGGAATCTGCAAGTGGATATTTGAC	CTGATAGGGAGAGCTCTGCTGCTAG	Ohzeki et al., 2012
RF322T(synthetic repeat)	TGGCCGGCATCACCGGCGCCAGAGGTC	TGATATCGCCCAACAGTCCCCCGGCCA	This study
HPRT mRNA	GGACCCCACGAAGTGTTGG	CTGGCGATGTCAATAGGACTCCAG	Ohzeki et al., 2012
CENP-A mRNA	AAAGCTTCAGAAGAGCACACACCTCTTG	CATGTAAGGTGAGGAGATAGGCGTCCTC	This study
hMis18α mRNA	CATCCTGCTTCGCTGTGTTTCCTG	TTGAGTGAGCACCCCGCGCAGCAC	Ohzeki et al., 2012
hMis18β mRNA	GGTGCACCTCGCCTGGGACCTGTCGCGG	AACTAGGAAGGGCGCTTCCAAAACGACG	This study
M18BP1 mRNA	ACTAAGAGCCTCAGTCCAAGGAGTTCCTC	GGCACTGAATCTTTTGTTTGACCGGGAGG	This study
HJURP mRNA	GCCATCATCACCCCTGGGGTGCAG	GCAACACATGTAGATGAAGGAGCCTC	Ohzeki et al., 2012
KAT7 mRNA	TCTCCGCTACCTGCATAATTTTCAAGGC	TTGGAGTTGGACCTTTTGGCCTCTTTGG	This study
BRPF2 mRNA	CAGTCTCAGCGAAGCTCACAGCAGAGAG	CCGCAGCTCCATGGCGACCTGCTCCACC	This study
ING4 mRNA	AACTTTCAGCTCATGAGGGACCTAGACC	TTTTCCTCGGAGCTCAGGCTGCGGGCAC	This study
ING5 mRNA	AACTTCCAGCTGATGCGAGAGCTGGACC	TGGAGACAGCGTCTTCACCGTGGAGATG	This study
RSF1 mRNA	CTGCAGACAGATGGGAAAAATATTTGATC	GACACTCACAGAGGTACTTTAAGAGTGC	This study

Supplemental references

Avvakumov, N., Lalonde, M.E., Saksouk, N., Paquet, E., Glass, K.C., Landry, A.J., Doyon, Y., Cayrou, C., Robitaille, G.A., Richard, D.E., et al. (2012). Conserved molecular interactions within the HBO1 acetyltransferase complexes regulate cell proliferation. Mol Cell Biol. *32*, 689-703.

Mishima, Y., Miyagi, S., Saraya, A., Negishi, M., Endoh, M., Endo, T.A., Toyoda, T., Shinga, J., Katsumoto, T., Chiba, T., et al. (2011). The Hbo1-Brd1/Brpf2 complex is responsible for global acetylation of H3K14 and required for fetal liver erythropoiesis. Blood. *118*, 2443-53.

Müller, S., Montes, de Oca, R,, Lacoste, N., Dingli, F., Loew, D., Almouzni, G. (2014). Phosphorylation and DNA binding of HJURP determine its centromeric recruitment and function in CenH3(CENP-A) loading. Cell Rep. *8*, 190-203.

Ohta, S., Bukowski-Wills, J.C., Sanchez-Pulido, L., Alves. Fde. L., Wood, L., Chen, Z.A., Platani, M., Fischer, L., Hudson, D.F., Ponting, C.P., et al. (2010). The protein composition of mitotic chromosomes determined using multiclassifier combinatorial proteomics. Cell. *142*, 810-21.

Tachiwana, H., Müller, S., Blümer, J., Klare, K., Musacchio, A., Almouzni, G. (2015). HJURP involvement in de novo CenH3(CENP-A) and CENP-C recruitment. Cell Rep. *11*, 22-32.

Zasadzińska, E., Barnhart-Dailey, M.C., Kuich, P.H., Foltz, D.R. (2013). Dimerization of the CENP-A assembly factor HJURP is required for centromeric nucleosome deposition. EMBO J. *32*, 2113-24.
