# Supplementary material for: KAT7/HBO1/MYST2 Regulates CENP-A Chromatin Assembly by Antagonizing Suv39h1-Mediated Centromere Inactivation
Source: Dev Cell. 2016 Jun 6;37(5):413–27. doi: 10.1016/j.devcel.2016.05.006 (PMC4906249; doi:10.1016/j.devcel.2016.05.006)
Supplement: Document S2. Article plus Supplemental Information [file mmc2.pdf]

Article

Developmental Cell

KAT7/HBO1/MYST2 Regulates CENP-A Chromatin Assembly by Antagonizing Suv39h1-Mediated Centromere Inactivation

Graphical Abstract

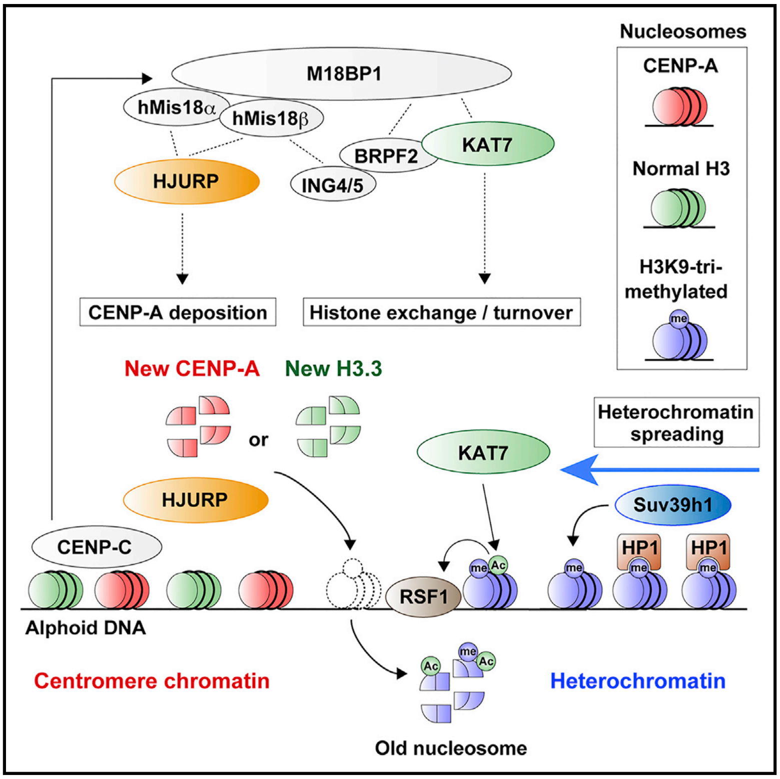

Authors

Jun-ichirou Ohzeki, Nobuaki Shono, Koichiro Otake, ..., Vladimir Larionov, William C. Earnshaw, Hiroshi Masumoto

Correspondence

masumoto@kazusa.or.jp

In Brief

Human centromeres contain histone H3 variant CENP-A and are flanked by pericentric heterochromatin. Spreading of heterochromatin into the centromere can impede centromere function. Ohzeki et al. demonstrate that the KAT7 histone acetyltransferase promotes CENP-A assembly and preserves centromere function by preventing H3K9-trimethylase Suv39h1-mediated heterochromatin invasion via a histone turnover mechanism.

Highlights

- The histone acetyltransferase KAT7 positively regulates centromeric CENP-A assembly
- Human Mis18 complex is a scaffold for assembly of KAT7 and HJURP, a CENP-A chaperone
- KAT7 or RSF1 stimulates histone turnover/exchange on alphoid DNA
- KAT7 antagonizes H3K9-trimethylase Suv39h1-mediated centromere inactivation

Ohzeki et al., 2016, Developmental Cell *37*, 413–427
June 6, 2016 © 2016 The Authors. Published by Elsevier Inc.
http://dx.doi.org/10.1016/j.devcel.2016.05.006

CellPress

Developmental Cell
Article

KAT7/HBO1/MYST2 Regulates CENP-A Chromatin Assembly by Antagonizing Suv39h1-Mediated Centromere Inactivation

Jun-ichirou Ohzeki,[1] Nobuaki Shono,[1] Koichiro Otake,[1] Nuno M.C. Martins,[2] Kazuto Kugou,[1] Hiroshi Kimura,[3] Takahiro Nagase,[4] Vladimir Larionov,[5] William C. Earnshaw,[2] and Hiroshi Masumoto[1,*]

[1]Laboratory of Cell Engineering, Department of Frontier Research, Kazusa DNA Research Institute, 2-6-7 Kazusa-Kamatari, Kisarazu 292-0818, Japan
[2]Wellcome Trust Centre for Cell Biology, University of Edinburgh, Edinburgh EH9 3BF, UK
[3]Department of Biological Sciences, Graduate School of Bioscience and Biotechnology, Tokyo Institute of Technology, Yokohama 226-8501, Japan
[4]Public Relations Team, Kazusa DNA Research Institute, Kisarazu 292-0818, Japan
[5]Genome Structure and Function Group, Developmental Therapeutics Branch, Center for Cancer Research, National Cancer Institute, National Institutes of Health, Bethesda, MD 20892, USA
*Correspondence: masumoto@kazusa.or.jp
http://dx.doi.org/10.1016/j.devcel.2016.05.006

SUMMARY

Centromere chromatin containing histone H3 variant CENP-A is required for accurate chromosome segregation as a foundation for kinetochore assembly. Human centromere chromatin assembles on a part of the long α-satellite (alphoid) DNA array, where it is flanked by pericentric heterochromatin. Heterochromatin spreads into adjacent chromatin and represses gene expression, and it can antagonize centromere function or CENP-A assembly. Here, we demonstrate an interaction between CENP-A assembly factor M18BP1 and acetyltransferase KAT7/HBO1/MYST2. Knocking out KAT7 in HeLa cells reduced centromeric CENP-A assembly. Mitotic chromosome misalignment and micronuclei formation increased in the knockout cells and were enhanced when the histone H3-K9 trimethylase Suv39h1 was overproduced. Tethering KAT7 to an ectopic alphoid DNA integration site removed heterochromatic H3K9me3 modification and was sufficient to stimulate new CENP-A or histone H3.3 assembly. Thus, KAT7-containing acetyltransferases associating with the Mis18 complex provides competence for histone turnover/exchange activity on alphoid DNA and prevents Suv39h1-mediated heterochromatin invasion into centromeres.

INTRODUCTION

The centromere is a specialized chromatin locus of eukaryotic chromosome containing the conserved specific histone H3 variant CENP-A (Earnshaw and Rothfield, 1985). During mitosis, kinetochore proteins assembled on centromeric chromatin direct accurate chromosomal segregation processes by interacting with microtubules (Cleveland et al., 2003; Allshire and Karpen, 2008; Fukagawa and Earnshaw, 2014). CENP-A is an essential component for the maintenance of centromere chromatin identity, providing a mark on which other centromere-specific proteins (i.e., the constitutive centromere associated network, CCAN) assemble throughout the cell cycle (Cheeseman and Desai, 2008). Sixteen CCAN proteins, including CENP-C and CENP-H, are currently known to assemble on nucleosomes containing CENP-A in vertebrates (Carroll et al., 2010; Black and Cleveland, 2011; Basilico et al., 2014).

In humans, CENP-A chromatin localizes to a portion of the α-satellite (alphoid) locus, a pleiomorphic, repetitive, megabase-sized locus composed of ~171-bp alphoid repeating units (Willard and Waye, 1987). The flanking portions of the huge alphoid locus are occupied by other chromatin structures containing mostly normal H3 highly trimethylated on lysine 9 (H3K9me3), creating the so-called pericentric heterochromatin (Sullivan and Karpen, 2004; Grewal and Jia, 2007). This pericentric heterochromatin structure containing H3K9-metyltransferases is conserved in regional centromeres from yeast to human (e.g., *Schizosaccharomyces pombe*, Clr4; *Drosophila melanogaster*, SU(VAR)3–9; mouse and human, Suv39h1, and Suv39h2). Suv39h1 and Suv39h2 double knockout mice lose H3K9 trimethylation from the pericentric regions of the chromosomes (Peters et al., 2001). HP1 proteins that interact with Suv39 can bind to H3K9me3 modifications and to each other. These interactions are thought to spread the pericentric heterochromatin structure and silence adjacent gene expression, as exemplified by position-effect variegation (PEV) (Muller, 1930; Allshire et al., 1994; Talbert and Henikoff, 2006; Grewal and Jia, 2007). As a result, it is important to understand how these different chromatin clusters, CENP-A chromatin or H3K9me3 chromatin, assemble and maintain themselves on the same underlying simple repetitive DNA sequences (alphoid DNA in human).

Excessive heterochromatin spreading (invasion) can inactivate centromere function. De novo centromere chromatin assembly accompanied by pericentromeric heterochromatin can occur on alphoid DNA sequences containing the CENP-B binding motif (CENP-B box). Stable human artificial chromosomes (HACs) can be obtained by transfecting appropriate DNAs into human

This is an open access article under the CC BY license (http://creativecommons.org/licenses/by/4.0/).

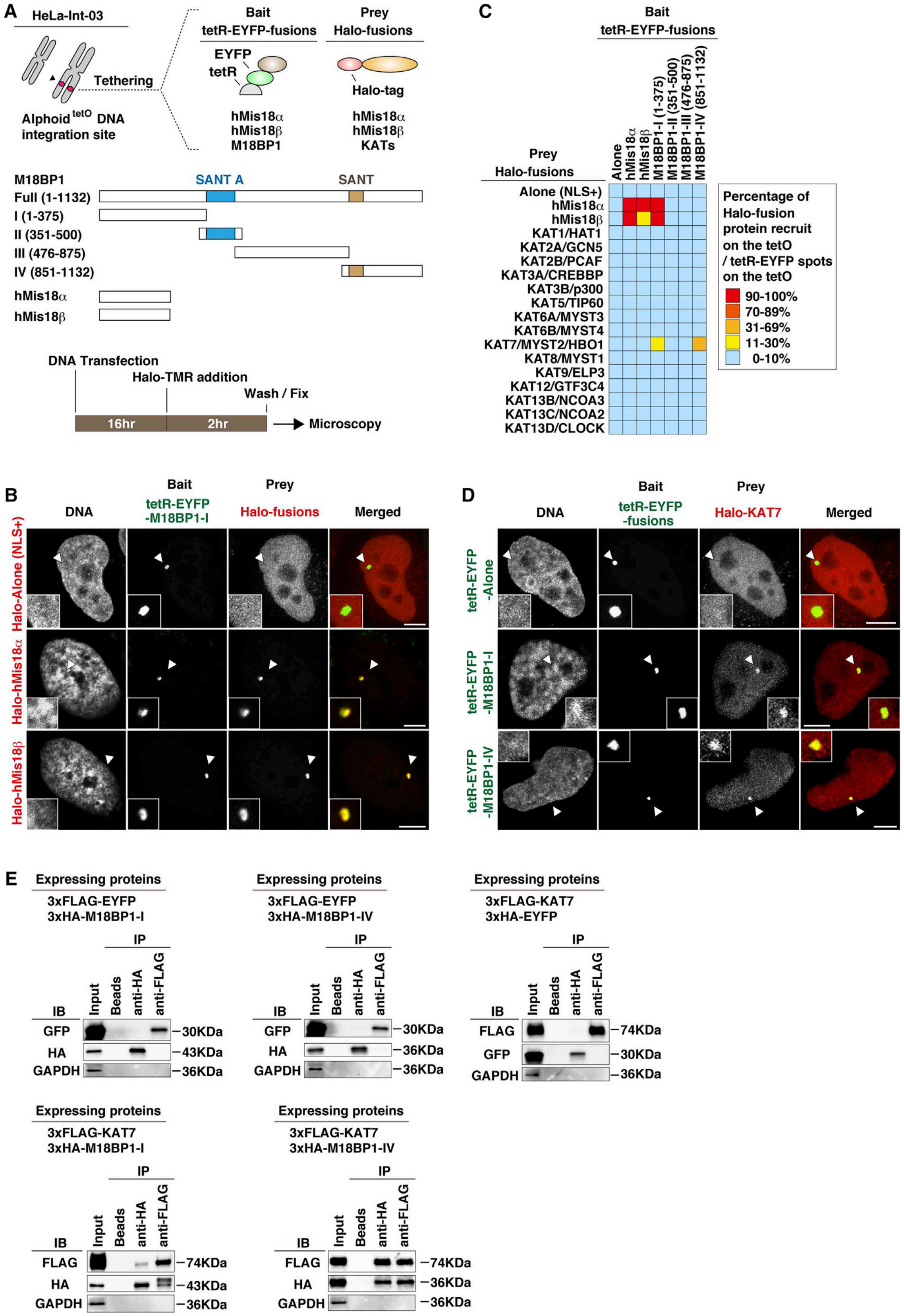

(legend on next page)

HT1080 cells and mouse embryonic fibroblasts (Harrington et al., 1997; Ikeno et al., 1998; Ohzeki et al., 2002, 2015; Okada et al., 2007). A new version of the HAC (alphoid^{tetO}-HAC) has also been generated using a synthetic alphoid array containing a tetracycline operator (tetO) sequence (the alphoid^{tetO} array) (Nakano et al., 2008). Tethering HP1 or Suv39h1 to the alphoid^{tetO}-HAC, as a tetracycline repressor (tetR) fusion protein, abrogates centromere chromatin assembly on the HAC. Consequently, the alphoid^{tetO}-HAC missegregates and forms micronuclei (Nakano et al., 2008; Cardinale et al., 2009; Ohzeki et al., 2012; Bergmann et al., 2012). The co-existence of centromere chromatin and heterochromatin at the same centromeric locus suggests the presence of intrinsic mechanisms that prevent the spread of heterochromatin into the centromere chromatin.

Histone acetyltransferase (HAT) activity antagonizes heterochromatin-mediated centromere inactivation. Many human cell lines, including HeLa cells, are incompetent for stable de novo centromere formation and have stronger H3K9-trimethylation activity on alphoid DNA than do HAC formation-competent HT1080 cells. We observed that tethering the HAT domain of p300 or PCAF protein (p300HD or PCAFHD) to the transfected alphoid^{tetO} DNA enabled active de novo centromere assembly and stable HAC formation, even in HeLa cells. Once established, those alphoid^{tetO}-HACs required no further assistance from HAT tethering for maintenance of the centromere chromatin. Furthermore, temporally regulated chromatin acetylation activity has been detected on HAC centromeres, as well as natural centromeres, during G_1 phase (Ohzeki et al., 2012). These results suggest that intrinsic HAT activity may be involved in the maintenance of established centromere chromatin, possibly by preventing strong heterochromatin from spreading onto centromeric alphoid DNA.

In each cell cycle, the amount of CENP-A nucleosomes is reduced by half during DNA replication and is not replenished until mitosis is complete (Jansen et al., 2007). Instead, they are replenished during the next G_1 phase by the CENP-A-specific deposition factor HJURP (Foltz et al., 2009; Dunleavy et al., 2009). The Mis18 protein complex (consisting of hMis18α, hMis18β, and M18BP1), an upstream factor required for the assembly of HJURP at centromeres, temporarily localizes at the centromere from mitotic exit to G_1 phase by interacting with CENP-C (Fujita et al., 2007; Maddox et al., 2007; Moree et al., 2011; Dambacher et al., 2012; McKinley and Cheeseman, 2014).

Interestingly, loss of CENP-A assembly following hMis18α depletion can be suppressed by treatment with the histone deacetylase inhibitor trichostatin A (TSA) (Fujita et al., 2007). Therefore, in the present study we hypothesized that the Mis18 complex may interact with specific HAT complexes at centromeres during early G_1 phase. However, which of the 17 human HAT activities is involved in centromere chromatin assembly and maintenance remained to be determined. We screened Mis18 complex-associated acetyltransferases with a newly developed protein-protein interaction assay using the ectopic alphoid^{tetO} array integration site, and found the KAT7/HBO1/MYST2 to be an M18BP1-interacting partner. KAT7 localized at endogenous centromeres in G_1 phase, and knocking out KAT7 reduced centromeric CENP-A assembly. A combination of knocking out KAT7 and overproducing Suv39h1 synthetically perturbed chromosomal segregation, leading to increased micronucleus formation. Furthermore, KAT7 tethered to a heterochromatinized alphoid^{tetO} site reduced H3K9me3 modifications and provided competence for new histone H3.3 or CENP-A assembly. Based on these results, we propose that KAT7 protects centromeres from the surrounding heterochromatin by promoting removal of H3 trimethylated on Lys9 via histone turnover/exchange activity. This activity may also contribute to increasing CENP-A deposition activity via interactions with M18BP1 scaffold in the canonical CENP-A replenishment pathway.

RESULTS

Screening for an Intrinsic Acetyltransferase that Interacts with the Human Mis18 Complex

To explore and identify HATs that interact with the Mis18 protein complex, we carried out a fluorescence microscopy-based interaction-trap assay on human chromosomes. HeLa-Int-03 cells have an ectopic integration of alphoid^{tetO} DNA that lacks detectable CENP-A assembly and has a high level of H3K9me3 modification (Ohzeki et al., 2012). We tethered Mis18 complex subunits to this ectopic alphoid^{tetO} DNA integration site as tetR-EYFP fusions and tested whether candidate Halo-tag-fused "prey" proteins were recruited (Figure 1A).

In this assay, tetR-EYFP-fused M18BP1 was divided into four domains because full-length M18BP1 has a stronger affinity for centromeres than tetR does for the alphoid^{tetO} DNA integration site. With full-length M18BP1 it is difficult to distinguish which tetR-EYFP-M18BP1 spot is the signal coming from the alphoid^{tetO} DNA integration site. First, we confirmed the known interactions among Mis18 complex subunits. Using fluorescent microscopy, we detected Halo-hMis18α and Halo-hMis18β recruitment to the tethered M18BP1 amino-terminal domain (1–375) (Figure 1B). Reciprocal recruitment of hMis18α and hMis18β was also confirmed (Figure 1C) (Fujita et al., 2007). When we expressed 15 of the >17 known human lysine acetyltransferases (KATs) (Allis et al., 2007) as Halo-tag fusions, only

Figure 1. M18BP1 Recruits the Acetyltransferase KAT7/HBO1/MYST2

(A) HeLa-Int-03 cells have an ectopic integration site of alphoid^{tetO} DNA repeats, which contains ~15,000 copies of the tetO sequences on a chromosome 21-derived synthetic alphoid DNA repeat. Halo-tag fused protein recruits were tested by tetR-fused protein tethering.

(B) M18BP1 tethering recruits hMis18α or hMis18β. Fluorescence images were obtained with DAPI (DNA), EYFP (green), and Halo-TMR ligand (red). The Halo tag covalently binds to the Halo-TMR ligand via the enzymatic activity of the Halo tag. White arrowheads indicate the loci of tetR-EYFP-fusion protein spots on the alphoid^{tetO} DNA integration site. Scale bar, 5 μm.

(C) Heatmap summarizing. Each cell indicates a combination of tetR-EYFP- and Halo-fusion proteins. Colors indicate the observed frequency of recruitment of Halo-fusion proteins by tetR-EYFP-fusion protein tethering (percentage of cells with a detectable signal). See also Figure S1A.

(D) KAT7 recruited by M18BP1 tethering. Fluorescence images were obtained with DAPI (DNA), EYFP (green), and Halo-TMR ligand (red). White arrowheads indicate the loci of tetR-EYFP-fusion protein spots on the alphoid^{tetO} DNA integration site. Scale bar, 5 μm.

(E) Co-immunoprecipitation experiments. Transiently expressed 3xFLAG-KAT7 and 3xHA-M18PB1 peptides were harvested in the cytosolic extract and used for immunoprecipitation (IP).

CellPress

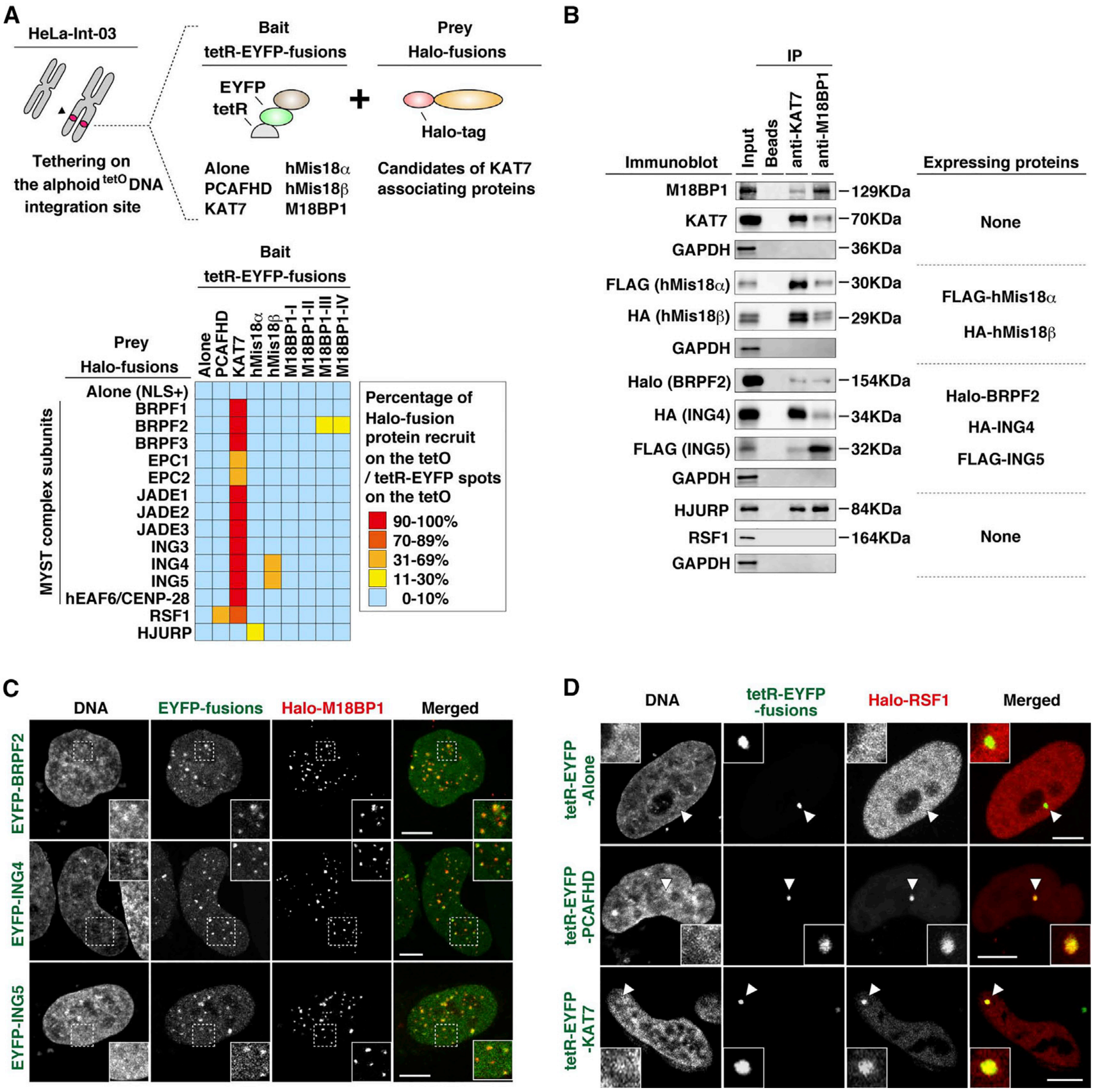

Figure 2. The M18BP1 and KAT7 Complex Associates with HJURP and RSF1

(A) Schematic diagram (top) and heatmap summarizing (bottom). Each cell indicates a combination of tetR-EYFP- and Halo-fusion proteins. See also Figures 1A, 1C, and S2C.

(B) Cytosolic extract was harvested and used for immunoprecipitation with anti-KAT7 and anti-M18BP1 antibodies. Ectopically expressed tagged proteins in each experiment are indicated on the right.

(C and D) EYFP-BRPF2, -ING4, or -ING5 co-localization with Halo-M18BP1 (C). HAT tethering recruits RSF1 (D). Fluorescence images were obtained with DAPI (DNA), EYFP (green), and Halo-TMR ligand (red). Arrowheads indicate the loci of tetR-EYFP-fusion protein spots on the alphoid^{tetO} DNA integration site. Scale bars, 5 μm.

KAT7 was recruited to tethered M18BP1 amino-terminal (1–375) and carboxyl-terminal (851–1,132) domains (Figures 1C, 1D, and S1A). KAT7 recruited the M18BP1 C-terminal domain and vice versa (Figure S1B). These protein interactions were confirmed by pull-down experiments (Figure 1E) and cytological analysis. Furthermore, EYFP-KAT7 co-localized with Halo-M18BP1 in HeLa cell nuclei (Figure S1C).

Mis18 and KAT7 Complex Includes HJURP and Recruits RSF1

KAT7 was first identified as a HAT that binds to ORC (HBO1) (Iizuka and Stillman, 1999), and belongs to the MYST family protein complexes. Most of the known MYST complexes are heterotetramers containing the MYST domain HAT, an ING subunit, EAF6/CENP-28, and EPL1-like platform proteins (Doyon et al., 2006) (Figure S2A). KAT7 is recruited to the origin of replication and contributes to MCM2-7 loading in G_1 phase (Miotto and Struhl, 2008). However, KAT7 function on centromere chromatin has not previously been described.

We therefore tested whether KAT7 tethering to the alphoid^{tetO} DNA array recruits other MYST complex family members. Indeed, MYST complex subunits plus remodeling and spacing factor 1 (RSF1) were recruited in the KAT7 tethering assay (Figure S2B). Next, we tested whether these proteins and HJURP

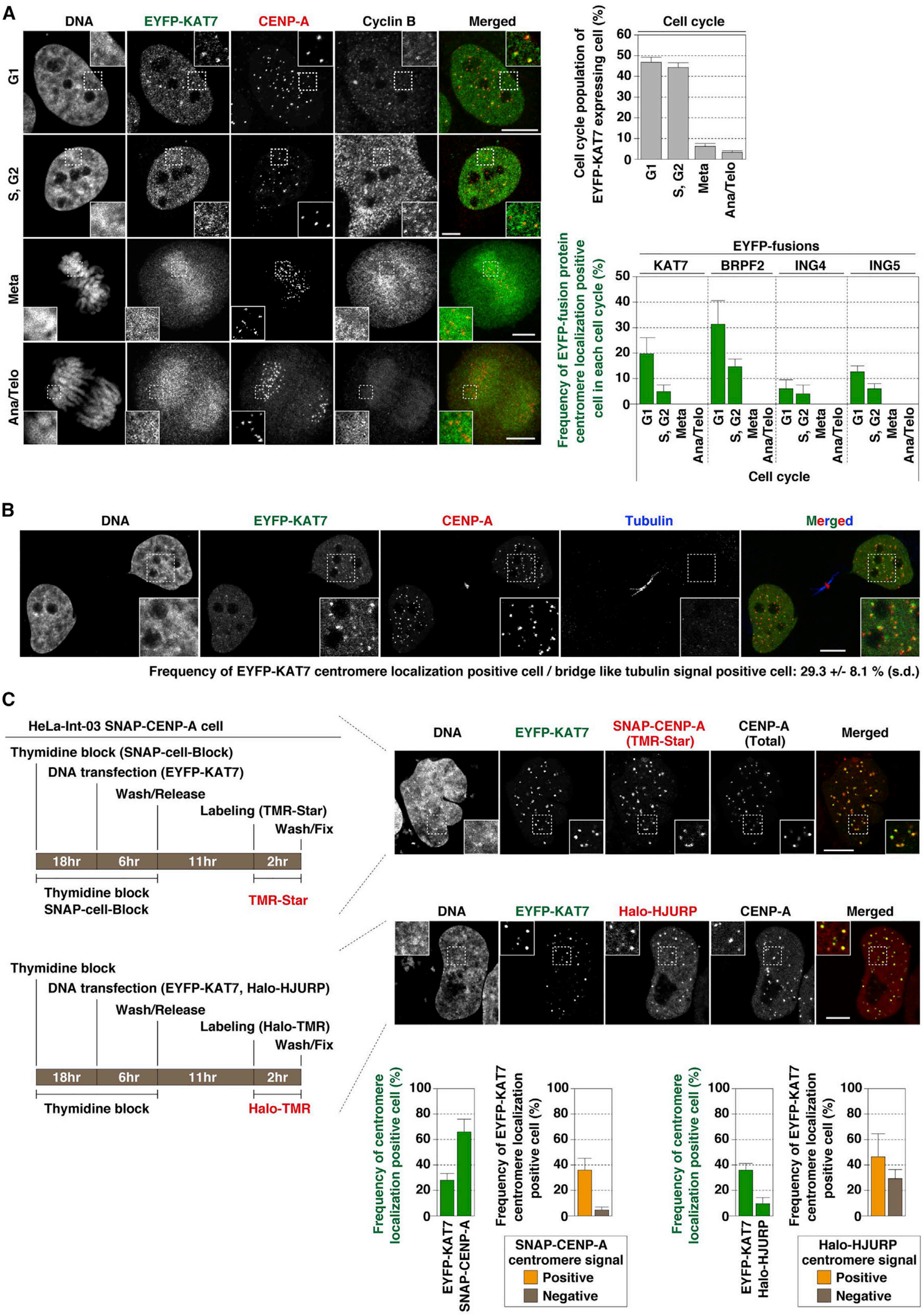

(legend on next page)

interact with Mis18 complex subunits (Figure 2A). BRPF2 was recruited by M18BP1 tethering, and ING4 and ING5 were recruited by Mis18β tethering. HJURP was recruited by hMis18α tethering (Figures 2A and S2C).

We also tested whether these recruited proteins co-immunoprecipitate with KAT7 or M18BP1 (Figures 2B and S2D). BRPF2, ING4, ING5, and HJURP co-immunoprecipitated with both KAT7 and M18BP1 (Figure 2B). Co-localization of EYFP-BRPF2, -ING4, or -ING5 with Halo-M18BP1 was also observed in cytological analysis (Figure 2C). Taken together, the results suggest that BRPF2, ING4, ING5, and HJURP interact with the Mis18 complex and KAT7.

Interestingly, RSF1 was also recruited by the PCAF HAT domain tethered at the ectopic site (Figures 2A and 2D), indicating that chromatin acetylation on the alphoid^{tetO} DNA promotes RSF1 binding.

KAT7 Localizes to Centromeres in G_1 Phase

Next, we asked whether tetR-EYFP-KAT7 and Halo-fused BRPF2, ING4, and ING5 co-localize with CENP-A at centromeres. The MYST proteins were distributed broadly throughout chromatin but, in a subpopulation of cells, showed some co-localization with CENP-A (Figure S3A). We then tested whether these proteins localize at centromeres in a cell-cycle-dependent manner.

HeLa-Int-03 cells stably expressing EYFP-KAT7 (Int-03 + KAT7) were created and co-immunostained with anti-CENP-A antibody for centromeres and anti-cyclin B antibody as a cell-cycle marker. EYFP-KAT7 localized at centromeres in G_1 phase (Figure 3A). Centromere localization of BRPF2, ING4, and ING5 in G_1 phase was also observed in transient protein expression experiments (Figures 3A and S3B).

Indeed, KAT7 and CENP-A co-localized more frequently in early G_1 cells having midbodies (29%) than in bulk G_1 cells (20%) (Figures 3A and 3B). KAT7 also co-localized with newly assembled SNAP-CENP-A and Halo-HJURP (Figure 3C). These results suggest an involvement of KAT7 in the CENP-A assembly pathway.

KAT7 Positively Regulates CENP-A Localization Level at Centromeres

We tested whether Mis18-KAT7 complex subunits were necessary for Halo-CENP-A localization at canonical centromeres using small interfering RNA (siRNA)-based protein depletion. Consistent with previous reports, M18BP1, hMis18α, hMis18β, and HJURP depletion substantially reduced Halo-CENP-A localization at the centromeres. Depletion of KAT7, BRPF2, ING4, and ING5 also reduced Halo-CENP-A localization at centromeres. Although these reductions were significant compared with the negative control, the effects were significantly weaker than those seen after HJURP depletion (Figure S4A).

To further investigate human KAT7 function in centromeres, we knocked out KAT7 in HeLa-Int-03 cells using the CRISPR/Cas system and isolated cell lines lacking KAT7 expression (KAT7KO; Figure 4A). Although KAT7 is essential for mouse post-gastrulation development, KAT7-knockout embryonic and immortalized fibroblasts are viable. A typical feature of mouse KAT7-knockout cells is reduced acetylation of the histone H3-K14 (H3K14ac) (Kueh et al., 2011). We checked H3K14ac levels by immunostaining and confirmed a strong reduction of nuclear H3K14ac signals in comparison with the parental HeLa-Int-03 cells in mixed cultures (Figures 4B, 4C, and S4B).

In chromatin immunoprecipitation (ChIP) assays, a strong reduction of H3K14ac levels was detected on chromosome X and 21 centromeric alphoid DNAs, ribosomal 5S DNA, and pericentromeric satellite DNA (Sat2) in KAT7KO cells. Levels of H3K14ac were rescued by *KAT7* expression in KAT7KO cells (Figure 4D). These results, together with the previous reports indicating that centromeric H3K14ac levels are low in fission yeast, fly, and human (Mellone et al., 2003; Sullivan and Karpen, 2004), suggest that H3K14 acetylating activity might be temporally regulated. To test this hypothesis we carried out ChIP with synchronized cells, and confirmed that H3K14ac levels on alphoid DNA are higher in early G_1 phase (Figure S4D).

We quantitated and compared centromeric CENP-A localization levels between KAT7KO cells and the parental HeLa cells in mixed culture. CENP-A levels were reduced by half at the endogenous centromeres in KAT7KO cells (Figures 4B, 4C, S4B, and S4C). The reduction of CENP-A levels on the chromosome 21 alphoid DNA was rescued by *KAT7* overexpression. The timing of new CENP-A localization at centromeres was similar in KAT7KO and HeLa-Int-03 cells (Figure S4E). However, the relative intensity of CENP-A at centromeres was significantly reduced (Figure S4F). These results suggest that KAT7 positively controls CENP-A assembly at centromeres.

Such a reduction of CENP-A level at centromeres might cause chromosome instability. We therefore evaluated chromosome instability by counting the frequencies of lagging and misaligned chromosomes in mitotic cells. Compared with HeLa-Int-03 cells, lagging and misaligned chromosomes were significantly (3.8-fold) increased in KAT7KO cells (Figure 4E). This instability was suppressed by expressing exogenous KAT7 (KAT7KO + KAT7 cells).

Figure 3. KAT7 and Other MYST Subunits Localize to the Centromere

(A) KAT7 and CENP-A co-localization. Fluorescence images were obtained with DAPI (DNA), EYFP (green), anti-CENP-A antibody (red), and anti-cyclin B antibody. Cell cycles were distinguished by images of DAPI and cyclin B staining, and these distributions are shown in the upper right panel. The observed frequency of KAT7 centromere localization positive cells (i.e., co-localization of KAT7 and CENP-A) is plotted in the bottom right panel. EYFP-BRPF2, -ING4, and -ING5 were also analyzed. See also Figure S3B.

(B) EYFP-KAT7 localized to centromeres at an earlier stage of G_1 phase, using the presence of an intercellular bridge of tubulin as a marker for early G_1 cells. Fluorescence images were obtained with DAPI (DNA), EYFP (green), anti-CENP-A antibody (red), and anti-tubulin antibody (blue).

(C) KAT7 co-localization with new CENP-A and Halo-HJURP in G_1 cells. (Left) Scheme for a quench chase pulse labeling of SNAP-CENP-A in G_1 phase or G_1 cell observation is shown on the left. Fluorescence images were obtained with DAPI (DNA), EYFP (green), TMR ligand (red; TMR-Star or Halo-TMR), and anti-CENP-A antibody. Data in this figure are presented as mean ± SD. (n = 3, >50 cells counted in each observation).

Scale bars, 5 μm.

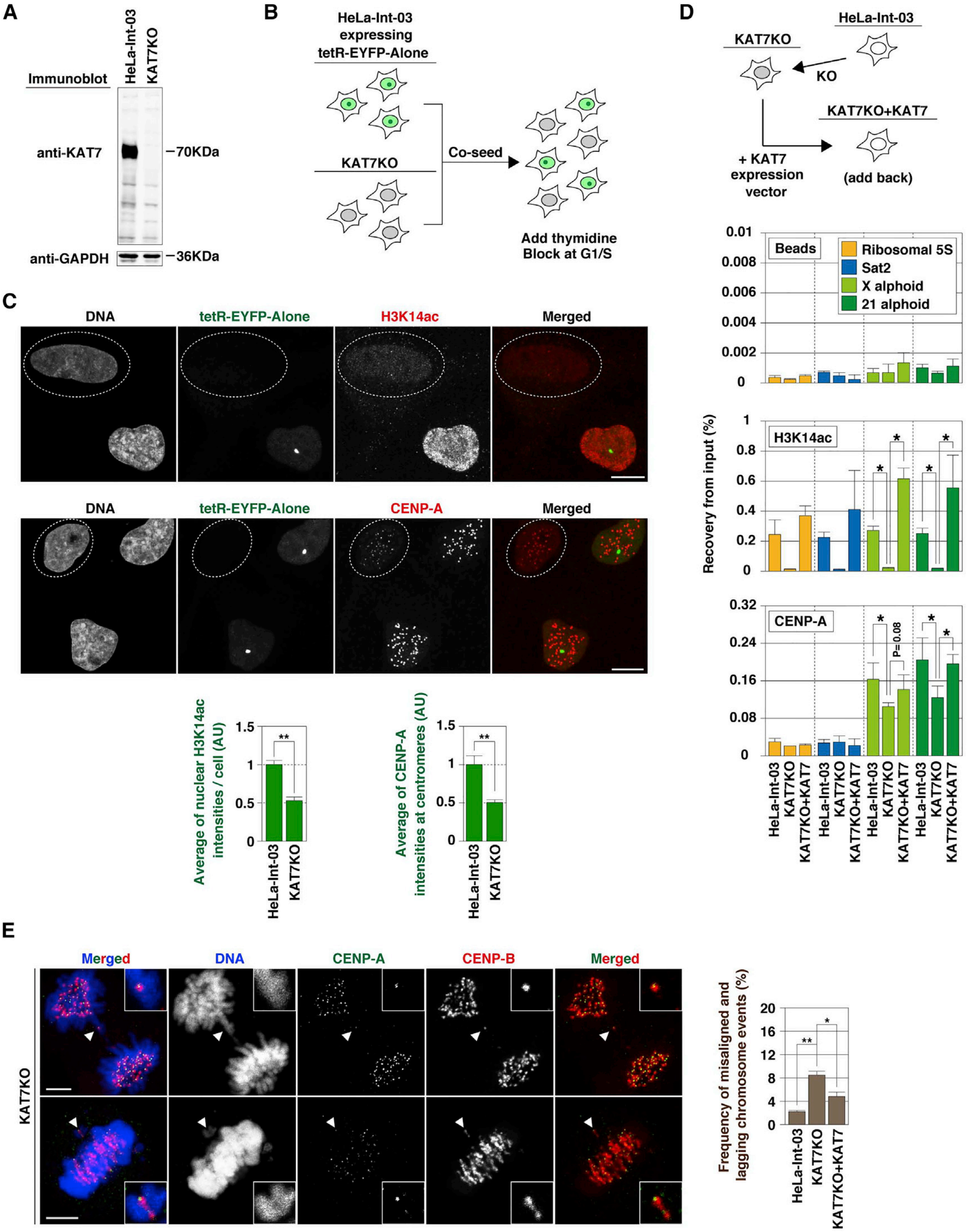

Figure 4. Centromeric CENP-A Assembly Was Reduced in KAT7-Knockout Cells

(A) Total cell lysate was applied for KAT7 immunoblotting. GAPDH was a loading control.

(B) Scheme of co-seeding. HeLa-Int-03 cells expressing tetR-EYFP alone were co-seeded with KAT7KO cells on a coverslip. The tetR-EYFP alone was used as a marker for distinguishing these cells.

(C) Fluorescent image quantification. Co-seeded cells were stained with DAPI, EYFP (green), and anti-H3K14ac or anti-CENP-A antibody (red). The KAT7KO cells are circled with white dotted lines. Scale bar, 10 μm. Signal intensities of the total nuclear H3K14ac (mean ± SE, n ≥ 50) and CENP-A at each centromere focus (mean ± SE, n ≥ 60) are shown at the bottom.

(D) ChIP analysis. ChIP was carried out with anti-H3K14ac antibody, anti-CENP-A antibody, or beads alone (no antibody). Data are presented as mean ± SD (n = 3). See also Figure S4D.

(legend continued on next page)

KAT7 Absence and Suv39h1 Overproduction Synthetically Induce Centromere Dysfunction

Centromeres are flanked by heterochromatin, and we previously showed that excess heterochromatin is incompatible with centromere maintenance and CENP-A assembly (Nakano et al., 2008; Cardinale et al., 2009). We therefore investigated whether KAT7 helps centromeres to resist invasion by heterochromatin by creating HeLa-Int-03 and KAT7KO cells overexpressing Suv39h1 (Figures 5A, 5B, and S5A). Suv39h1 tethering to the alphoid^{tetO}-HAC destabilized its centromere function, resulting in a high HAC loss rate (~22% per cell division) (Ohzeki et al., 2012). Suv39h1 is responsible for pericentric H3K9me3 modification in mice (Peters et al., 2001) and enhances H3K9me3 levels on alphoid DNAs in human HT1080 cells (Ohzeki et al., 2012). Therefore, we anticipated a possible synthetic effect of the absence of KAT7 and Suv39h1 overproduction in centromere function.

We quantitated chromosome stability by counting the frequencies of lagging and misaligned chromosomes in mitotic cells. Suv39h1 overproduction (Int-03 + Suv39h1 cells) also increased misaligned chromosomes by 3.6-fold. Strikingly, in KAT7KO + Suv39h1 cells, misaligned chromosomes were observed 10-fold more frequently, and some of the misaligned or lagging chromosomes were negative for centromeric CENP-A (Figure 5C).

Missegregated chromosomes often form micronuclei. The frequency of micronucleus formation correlated with that of misaligned chromosomes, and both loss of KAT7 and Suv39h1 overproduction synthetically increased micronucleus formation. In KAT7KO, Int-03 + Suv39h1, and KAT7KO + Suv39h1 cells, micronucleus formation was increased by 3.7-, 4.4-, or 11-fold, respectively, compared with HeLa-Int-03 cells (Figure 5D).

Micronucleus formation also occurs subsequent to DNA breakage, particularly if the breakage produces an acentric chromosome fragment. KAT7 depletion could lead to DNA breakage, as KAT7 also contributes to DNA replication, and increased Suv39h1 protein levels harm DNA repair in the heterochromatin (Liu et al., 2013). Therefore, to investigate whether micronucleus formation was caused by a loss of centromere DNA or function, we determined the frequency of CENP-B-positive and -negative micronuclei. CENP-B can bind both active and inactive centromeres on alphoid DNA (Earnshaw et al., 1989) through the CENP-B box (Masumoto et al., 1989). Therefore, CENP-B-positive micronuclei would be formed by events in which a centromere was present but had become inactivated, and thus presumably not as a result of chromosome breakage. In HeLa-Int-03 cells, most micronuclei were negative for CENP-B. In KAT7KO and Int-03 + Suv39h1 cells, most micronuclei were CENP-B negative (3.2-fold and 3.9-fold increased versus HeLa-Int-03, respectively), but a significant population of CENP-B-positive micronuclei was also observed (13-fold [$p < 0.005$] and 12-fold [$p < 0.005$] increase for KAT7KO and Int-03 + Suv39h1 versus HeLa-Int-03, respectively). Interestingly, in KAT7KO + Suv39h1 cells, CENP-B-positive, but not -negative micronuclei were significantly increased compared with levels seen after KAT7KO (positive micronuclei, 9.3-fold increase [$p < 0.005$]; negative micronuclei, 1.3-fold increase [$p = 0.2$]) and Int-03 + Suv39h1 cells (positive micronuclei, 10-fold increase [$p < 0.005$]; negative micronuclei, 1.0-fold increase [$p = 0.83$]) (Figure 5D). We confirmed the reduction of centromeric CENP-A intensity (>74% reduction) at the CENP-B-positive micronuclei in all of these cases (Figure 5E).

Thus, either the absence of KAT7 or Suv39h1 overproduction predominantly results in formation of CENP-B-negative micronuclei, presumably due to defects in DNA replication or repair. However, the combination of KAT7 depletion and Suv39h1 overproduction increases CENP-B-positive micronuclei, presumably reflecting a loss of centromere function due to heterochromatin spreading. We detected a 1.2- to 1.3-fold increase in H3K9me3 levels on alphoid DNAs in KAT7KO cells (Figure S5B). Thus, one function of KAT7 may be to antagonize the spread of heterochromatin.

KAT7 and RSF1 Antagonize H3K9me3 Modification and Contribute to Centromere Stability

A combination of KAT7 absence and Suv39h1 overproduction synthetically affects chromosome instability (Figure 5). This could be explained by the reduction in centromeric CENP-A assembly caused by the absence of KAT7, if that increases centromere sensitivity to Suv39h1-mediated heterochromatin invasion. We therefore decided to test whether KAT7 and its associated proteins can antagonize H3K9 trimethylation. We focused on the non-centromeric ectopic alphoid^{tetO} DNA integration site because it has high levels of H3K9me3 (Ohzeki et al., 2012).

Tethering tetR-EYFP alone did not affect the bright H3K9me3 staining pattern at the ectopic alphoid^{tetO} site. In almost all observed cells (99%), the tetR-EYFP spot clearly overlapped with the H3K9me3 signal. In contrast, tethering tetR-EYFP-KAT7 reduced the H3K9me3 signal in 19% of tetR-EYFP spots (Figures 6A and 6B). In controls, tethering the tetR-EYFP-KAT7 G485A acetyltransferase catalytic domain mutant was less effective than the normal KAT7 (4.7%). No significant difference was found in the frequencies between 2 hr of tethering in cells synchronized at G_1 phase and 24 hr of continuous tethering in asynchronously cultured cells (Figure 6C). Thus, the reduction in H3K9me3 appears to be independent of effects on DNA replication.

We counted the number of cells exhibiting reduced H3K9me3 staining following tethering of several tetR-EYFP-fusion proteins in G_1 phase. Tethered BRPF2, ING4, or ING5 were less potent than tethered KAT7. M18BP1 domain IV tethering reduced H3K9me3 levels to an extent similar to that seen after tethering KAT7 (Figure 6D). Thus, the reduction in H3K9me3 was possibly due to endogenous KAT7 recruitment. Interestingly, RSF1 was the most potent factor at reducing H3K9 methylation (by 40%) (Figure 6D). The H3K9me3 reduction by KAT7 and RSF1 was also confirmed by ChIP-qPCR analysis (Figure S6A). We also

(E) Chromosome missegregation. Mitotic cells were stained with DAPI (DNA; blue), anti-CENP-A antibody (green), and anti-CENP-B antibody (red). White arrowheads indicate lagging or misaligned chromosomes. Scale bar, 5 μm. Frequencies of misaligned and lagging chromosome events were plotted (>100 metaphase cells were counted in each cell line). Data are presented as mean ± SE. (n = 6).
*$p < 0.05$, **$p < 0.005$ (t test).

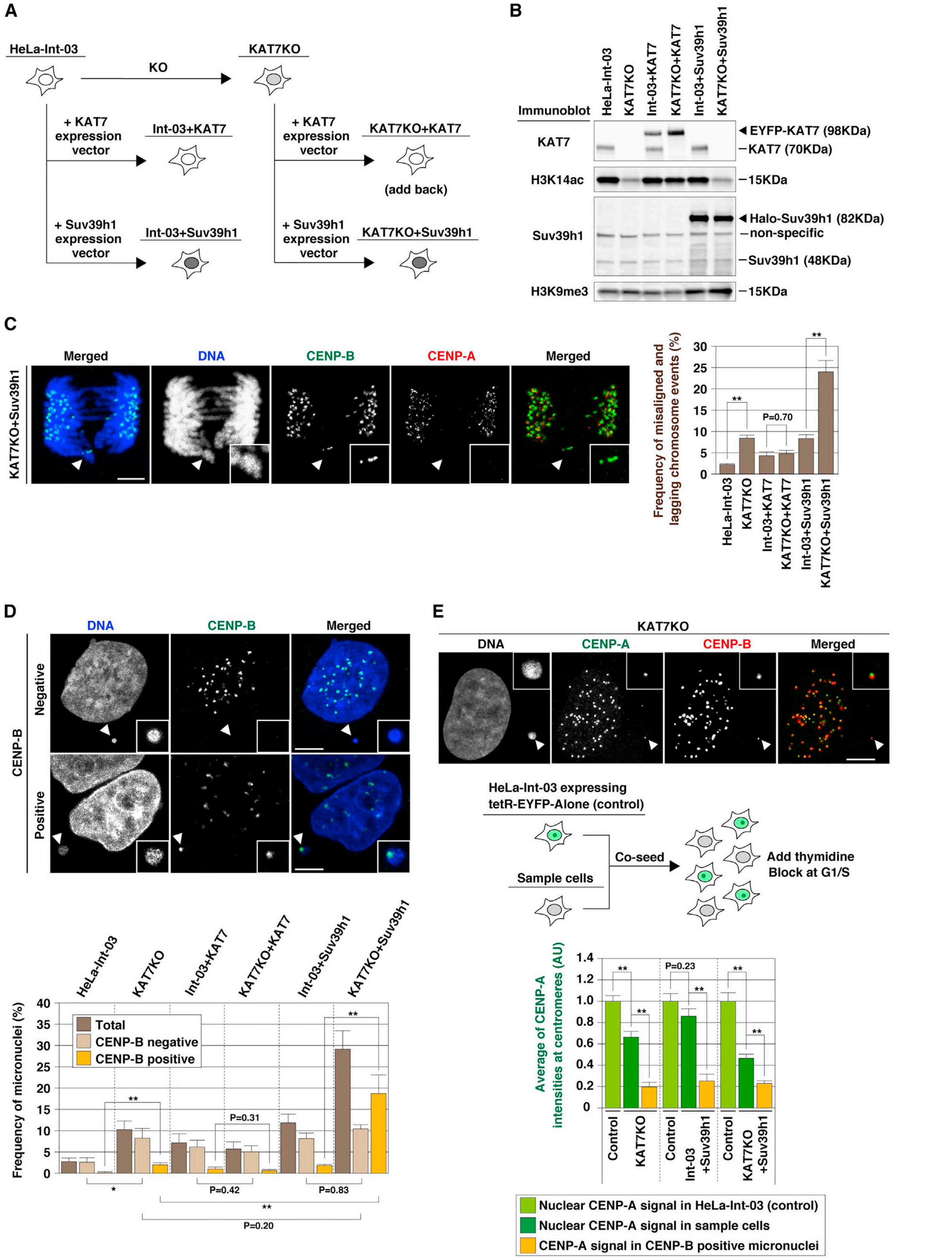

(legend on next page)

tethered tetR-EYFP-JMJD2B, a H3K9me3 demethylase, to the alphoid^{tetO} DNA integration site. As expected, JMJD2B reduced H3K9 trimethylation (Figures 6B and 6D).

We next investigated the role of RSF1 as a factor reducing H3K9me3 using cells overexpressing Suv39h1 to induce heterochromatin spreading, and measuring CENP-B-positive micronuclei as a measure of loss of centromere function. Comparing HeLa-Int-03 and Int-03 + Suv39h1 cells, RSF1 depletion significantly increased CENP-B-positive micronucleus formation (2.6-fold, $p < 0.05$), but not CENP-B-negative micronuclei (1.3-fold increase, $p = 0.12$) (Figure 6E). Next, centromeric CENP-A signal intensities both in nuclei and CENP-B-positive micronuclei were quantitated. RSF1 depletion and Suv39h1 overproduction synergistically reduced normal centromeric CENP-A levels in nuclei (45% reduction), and CENP-A reduction was even more pronounced in the CENP-B-positive micronuclei (81% reduction) (Figure S6B). These data suggest that RSF1 depletion synthetically destabilizes centromere function in the presence of Suv39h1 overproduction. A similar synthetic destabilization was confirmed following KAT7 depletion (Figure 6E). On the other hand, M18BP1 depletion, which disrupts the CENP-A assembly machinery (Figure S4A), destabilized centromere function at a similarly high level independent of Suv39h1 overproduction (Figures 6E and S6B).

KAT7 and RSF1 Promote Histone Exchange/Turnover

The observed H3K9me3 reduction could be explained by demethylation at the H3-K9 residue or histone H3 eviction by a histone turnover mechanism. To detect histone H3 turnover, we focused on the histone H3 variant, H3.3, because its deposition is independent of DNA replication (Szenker et al., 2011). We also tested whether newly expressed Halo-histone H3.3 assembles at the alphoid^{tetO} DNA integration site following tetR-EYFP-KAT7 or tetR-EYFP-RSF1 tethering (Figures 7A–7C). Halo-histone H3.3 efficiently assembled after both KAT7 and RSF1 tethering in G_1 phase (Figure 7C). When Halo-CENP-A was expressed, it was similarly assembled to both KAT7 and RSF1 tethering sites (Figure 7D). Although KAT7 or RSF1 tethering promoted CENP-A and histone H3.3 assembly, neither histone H3.3 nor CENP-A assembly was induced solely by the reduction of H3K9 trimethylation (i.e., JMJD2B tethering) (Figures 7C and 7D). These observations suggest that KAT7 or RSF1 tethering at the alphoid^{tetO} DNA integration site promoted histone turnover/exchange, resulting in H3.3 and CENP-A assembly (see also Shono et al., 2015).

Together, these results suggest that KAT7 and RSF1 prevent Suv39h1-induced heterochromatin invasion, possibly via a histone turnover/exchange mechanism.

DISCUSSION

In this study, we identified KAT7 as a HAT that interacts with M18BP1 to positively regulate centromeric CENP-A assembly and prevent Suv39h1-mediated centromere inactivation via a mechanism that involves increased histone turnover/exchange mediated by the Mis18 complex interacting with other factors.

The Mis18 complex is critical for epigenetic centromere maintenance, especially in CENP-A replenishment. In this study, we show that the M18BP1 amino-terminal region recruited hMis18α and hMis18β while its C-terminal region recruited KAT7 and BRPF2. Both hMis18α and hMis18β are involved in the efficient HJURP-CENP-A assembly pathway. Tethering KAT7 to the alphoid^{tetO} DNA resulted in histone turnover/exchange activity. In KAT7KO cells, centromeric CENP-A assembly levels decreased by half. Thus, coordinated assembly of HJURP and KAT7 on the same M18BP1 scaffold via other interacting proteins may be necessary for normal CENP-A deposition at centromeres. Interestingly, tethered KAT7 recruited RSF1 to the acetylated chromatin. RSF1 reportedly co-purifies with CENP-A nucleosomes and contributes to stable CENP-A assembly (Perpelescu et al., 2009). RSF1 recruited by KAT7 may also regulate centromeric CENP-A assembly during Mis18 complex formation, as RSF1 tethering strongly promotes H3.3 and CENP-A turnover/exchange.

PEV around pericentric heterochromatin is thought to result from a stochastic balance between heterochromatin spreading and its containment. In fission yeast, the cluster of tRNA genes between the centromere core (cnt) and pericentric (dg and dh) DNA functions as a chromatin boundary to prevent such heterochromatin spread. Deletion of those tRNA genes causes chromosomal instability, resulting from reduced CENP-A^{Cnp1} assembly (Scott et al., 2006). In *D. melanogaster*, GAGA factor and FACT achieve transcription-coupled histone H3.3 turnover at their binding sites located between the white gene and pericentric heterochromatin. This H3.3 replacement counteracts spreading of the H3-K9-methylated chromatin (Nakayama et al., 2007). Thus, heterochromatin spreading can be prevented by transcriptional activity or associated histone turnover.

Although no obvious boundary DNA sequence element akin to fission yeast tRNA genes has been identified between the

Figure 5. Combination of KAT7 Absence and Suv39h1 Overproduction Synthetically Destabilizes Chromosome Segregation

(A) Graphic explanation of the cell lines.

(B) Total cell lysate was applied for immunoblotting.

(C) Frequencies of misaligned and lagging chromosomes. Cells were stained with DAPI (DNA; blue), anti-CENP-B antibody (green), and anti-CENP-A antibody (red). White arrowheads indicate a lagging chromosome. Frequencies of the misaligned and lagging chromosomes were plotted (>100 metaphase cells were counted in each cell line). Data are presented as mean ± SE ($n \geq 3$).

(D) Frequencies of the CENP-B-positive and -negative micronuclei. Cells were stained with DAPI (DNA; blue) and anti-CENP-B antibody (green). White arrowheads indicate the locations of micronuclei. Frequencies of the micronuclei were plotted (>200 cells were counted in each cell line). Data are presented as mean ± SD (n = 3).

(E) CENP-A intensity at centromeres, including CENP-B-positive micronuclei. Cells were stained with DAPI, anti-CENP-A antibody (green), and anti-CENP-B antibody (red). White arrowheads indicate the locations of micronuclei. Control (HeLa-Int-03 expressing tetR-EYFP) and target cells were co-seeded on coverslips, and arrested at G_1/S with thymidine. CENP-A signal at centromeres including the CENP-B-positive micronuclei was quantitated and plotted in the bottom panel. Data are presented as mean ± SE ($n \geq 10$ micronuclei).

*$p < 0.05$, **$p < 0.005$ (t test). Scale bars, 5 μm.

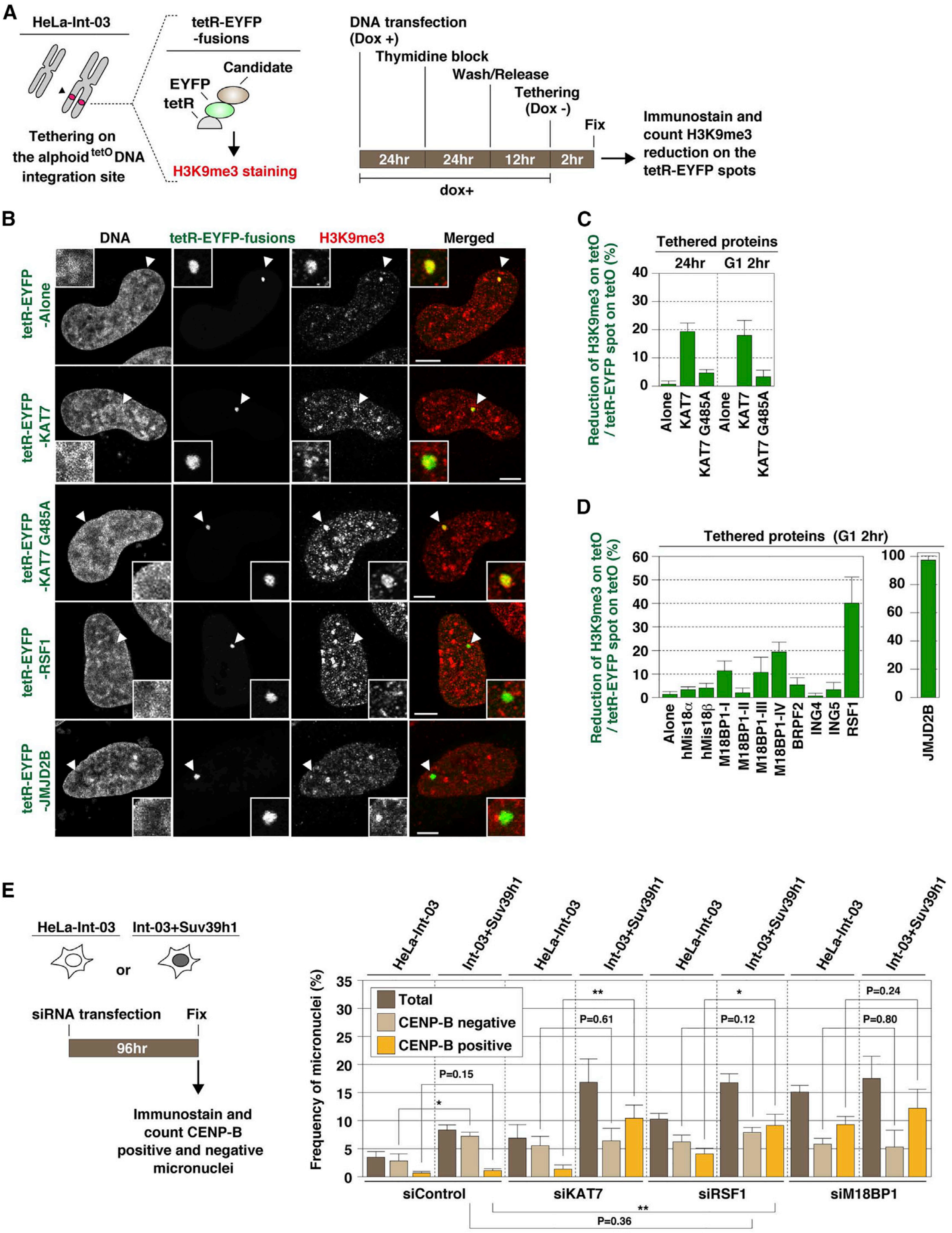

Figure 6. KAT7 and RSF1 Tetherings Reduce H3K9me3 Modification

(A) Scheme of candidate protein tethering and H3K9me3 immunostaining in G_1 phase.

(B) Examples of H3K9me3 staining. Fluorescence images were obtained with DAPI (DNA), EYFP (green), and anti-H3K9me3 antibody (red). Arrowheads indicate loci of tetR-EYFP-fusion protein spots on the alphoid^{tetO} DNA integration site. Scale bars, 5 μm.

(C and D) Observed H3K9me3 reduction frequency was plotted (>50 tetR-EYFP spots were counted in each sample). Data are presented as mean ± SD (n = 3).

(E) Scheme of micronuclei formation combined with siRNA transfection (left). Cells were stained and observed as shown in Figure 5D. The frequencies of the CENP-B-positive and -negative micronuclei were plotted (>200 cells were counted in each cell line). Data are presented as mean ± SD (n = 3). *p < 0.05, **p < 0.005 (t test).

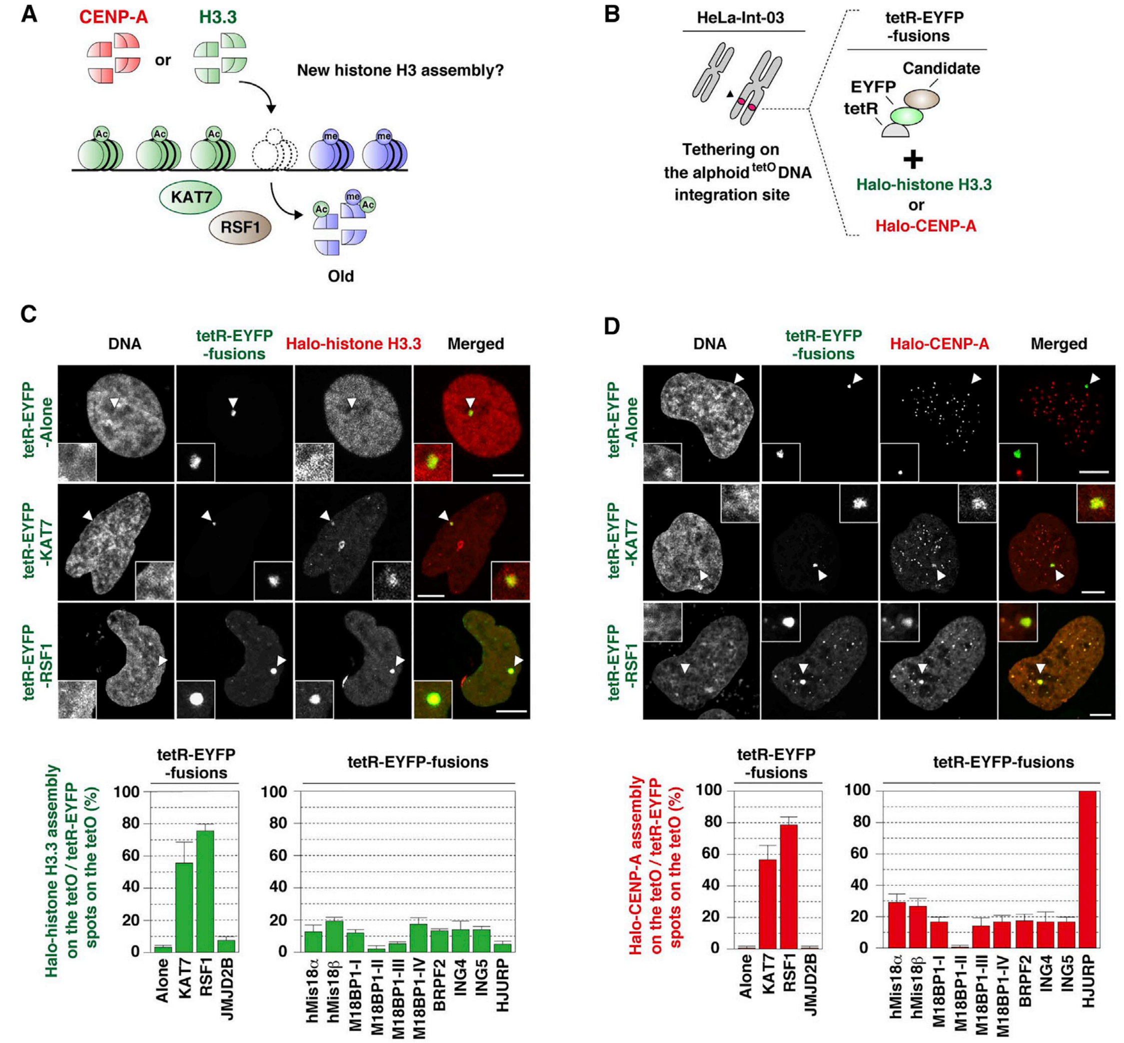

Figure 7. KAT7 and RSF1 Promote Histone Exchange/Turnover

(A) A model for histone turnover. Note that KAT7 acetylates not only histone H3 but also H4 (Miotto and Struhl, 2010).

(B) Scheme for experiments.

(C and D) Fluorescence images were obtained with DAPI (DNA), EYFP (green), and Halo-TMR ligand (red). The Halo-histone H3.3 (C) or -CENP-A (D) assembly frequency was plotted (>50 tetR-EYFP spots were counted in each sample). Data are presented as mean ± SD (n = 3). Arrowheads indicate loci of tetR-EYFP-fusion protein spots on the alphoid^{tetO} DNA integration site. Scale bars, 5 μm.

centromere and heterochromatin on the long alphoid repetitive arrays in human cells, we found that KAT7 acetyltransferase (in the MYST2 HAT complex) was recruited by M18BP1. Tethering KAT7 or RSF1 to an ectopic non-centromeric alphoid^{tetO} DNA array was sufficient to reduce the level of H3K9me3 modification, and this reaction was coupled with new H3.3 or CENP-A assembly. Depletion of KAT7 or RSF1 combined with Suv39h1 overproduction resulted in centromere dysfunction.

These results strongly suggest that M18BP1 recruits KAT7 to the centromere to prevent Suv39h1-mediated heterochromatin spreading both via acetylation and as a result of removal of H3K9me3 nucleosomes by histone turnover/exchange mechanisms associated with RSF1. Interestingly, fission yeast KAT7^{Mst2} has been shown to antagonize pericentric heterochromatin nucleation by promoting transcription (Reddy et al., 2011) and Suv39h1^{Clr4}-mediated gene silencing (Wang et al., 2015). In human mesenchymal stem cells, loss of Suv39h1 methyltransferase activity increases the amount of alphoid DNA transcripts (Zhang et al., 2015). In addition, RSF1 enhances transcription on acetylated chromatin but not heterochromatic templates in vitro (Loyola et al., 2001). Tethering KAT7 or RSF1 to the alphoid^{tetO} array indeed enhanced its transcription level by 1.7- to 2.1-fold (Figure S7B). Transcriptional events and/or KAT7 assembly on alphoid DNAs could stimulate histone turnover. Interestingly, transcription-coupled CENP-A incorporation events in *D. melanogaster* and *S. pombe* have recently been reported (Chen et al., 2015; Catania et al., 2015).

Simultaneous absence of KAT7 and overproduction of Suv39h1 caused a synthetic chromosome missegregation phenotype. Possible mechanisms for H3K9me3-mediated centromere inactivation have been suggested by previous studies. Tethering H3-K9 trimethylases or HP1 induces the sequential disassembly of

centromere proteins. CENP-H is lost, followed by CENP-C and CENP-A (Nakano et al., 2008; Cardinale et al., 2009). Heterochromatin invasion of the centromere chromatin apparently destabilizes these centromere proteins and may eventually inactivate the kinetochore assembly and/or CENP-A replenishment pathway. Although H3K9me3 can promote centromere inactivation, it is observed by super-resolution microscopy between CENP-A-containing nucleosomes, even in a functional centromere (Ribeiro et al., 2010). Possibly, isolated patches of H3K9me3 may be permissive for centromere function unless HP1 assembles at this site (Martins et al., 2015). KAT7 or RSF1 could remove the H3K9me3 marks before HP1 assembly and/or CENP-A replenishment.

In the competition between centromere and heterochromatin assembly, alphoid DNA sequences may also play important roles. KAT7 and RSF1 tethering assembled CENP-A on the alphoid^{tetO} DNA but not on a synthetic non-alphoid DNA^{tetO} array, RF322^{tetO} (Ohzeki et al., 2002) (Figure S7C). Some properties of alphoid DNA, such as the CENP-B box/CENP-B interaction, are necessary for this CENP-A assembly activity (Okada et al., 2007). CENP-B stabilizes preassembled CENP-A, and recruits CENP-C for CENP-A and kinetochore assembly (Suzuki et al., 2004; Fachinetti et al., 2013, 2015; Fujita et al., 2015).

On the other hand, CENP-B can also efficiently heterochromatinize the ectopically integrated alphoid DNA in mouse chromosomes via interactions with Suv39h1 (Okada et al., 2007). Disrupting heterochromatin by H3K9 acetylation is required for de novo CENP-A and HAC assembly on satellite DNAs in heterochromatin-rich cells (Ohzeki et al., 2012). Acetylation activity on alphoid DNA-associated chromatin might bypass the requirement for hMis18α in CENP-A accumulation. As previously reported, TSA treatment suppresses CENP-A centromere localization defects in cells lacking hMis18α (Fujita et al., 2007), and we confirmed that KAT7 overproduction also bypasses the requirement for hMis18α (Figure S7D). Although it is not known how acetylation bypasses the requirement for hMis18α, alphoid DNA and CENP-B might function in acetylation-induced CENP-A assembly. It is also interesting to speculate whether the acetylation and histone turnover/exchange can switch between the heterochromatin-antagonizing and heterochromatin-promoting properties of CENP-B.

Centromere DNA sequences are widely divergent between species. Although once established the centromere is epigenetically maintained through CENP-A replenishment mechanisms, it is not known how current centromeres were originally formed at their present genomic loci de novo. We recently found that CENP-C (or CENP-I) tethering is sufficient for de novo endogenous CENP-A assembly on the ectopically integrated alphoid^{tetO} array. This de novo endogenous CENP-A assembly pathway requires M18BP1. However, tethering of CENP-A replenishment factors (M18BP1, hMis18α, hMis18β, KAT7, or RSF1) was insufficient to promote de novo endogenous CENP-A assembly (Shono et al., 2015), even though those replenishment factors were sufficient for de novo assembly of overexpressed CENP-A at the ectopic site. Interestingly, in those experiments HJURP tethering could promote endogenous CENP-A assembly, indicating that the CENP-A pool size was not a limiting factor for de novo assembly. Taken together, these results suggest that for de novo endogenous CENP-A assembly, some properties of CENP-C (or CENP-I) as an epigenetic and a functional centromere mark are also necessary in addition to the recruitment of CENP-A replenishing factors by M18BP1, although CENP-A overproduction or forced DNA binding of HJURP via tetR can bypass this constraint. Further studies are required to determine how centromere integrity is established and maintained by these CENP-A de novo assembly and replenishing factors.

EXPERIMENTAL PROCEDURES

Cell Culture, Transfection and Staining

Human cultured cells, 293T, HeLa, HeLa-Int03, and HeLa-Int-03 derivative cells were grown in a DMEM Glutamax I (Invitrogen), supplemented with 10% fetal bovine serum at 37°C in a 5% CO_2 atmosphere. For transfections, Lipofectamine 2000 (Invitrogen) or FuGENE HD (Promega) transfection reagent was used for siRNA or plasmid vector DNA, respectively. Used siRNA sequences and plasmid vectors are provided in Tables S1 and S2. The cell lines used in this study are provided in Table S3. Specifically, HeLa-Int-03 derived cell lines expressing EYFP-KAT7 or Halo-Suv39h1 were produced with the Jump-in integration system (Life Technologies) using pJ3-EYFP-KAT7 or pJ4IB-Halo7-Suv39h1 gene-expression vector by co-transfection with pJTI PhiC31 Int vector (expression vector for Phi-C31 integrase; Life Technologies). *KAT7* gene knockout was carried out with a CRISPR/Cas9-mediated gene deletion method by transient co-transfection of plasmids pJ4IB-Cas9 and pU6CR-KAT7 (expression vector for Cas9 protein and guide RNA against *KAT7* gene, respectively). The targeted sequence of *KAT7* gene was 5′-GACATGTCCCTGAAGGACTCAGG. In isolated single clones, *KAT7* gene deletions with frameshift mutations at all the alleles were confirmed by sequencing of genomic PCR products using an MiSeq sequencer (Illumina) (more than 9,000 reads were obtained in each sample). The PCR primer set used for *KAT7* gene locus was 5′-CCATGATTAGCAACTCAGGAGAAATACAG and 5′-CAGGGTCTCATGTGTACTCCTAATTTC, respectively. For Halo-tag labeling, Halo-TMR ligand (Promega, G8251) was added to the cell-culture medium (1 nM). For SNAP-tag labeling, 3 μM of SNAP-Cell Block reagent (NEB) was used for blocking of pre-existing SNAP-fused protein, and 1 μM of SNAP-Cell TMR-Star reagent (NEB) was used for newly expressed SNAP-fused protein staining.

ChIP and qPCR

The main ChIP procedure was described previously (Ohzeki et al., 2002; 2012), but in this study Protein G Dynabeads (Life Technologies, 1004D) were used instead of Protein G Sepharose. The antibodies used are provided in Table S4. Immunoprecipitated DNAs or harvested total RNA was quantified by real-time PCR. The primer sequences for quantification are provided in Table S5.

Protein Immunoprecipitation and Immunoblotting

HeLa or 293T cells were transfected with protein expression vectors and harvested 2 days after transfection. Cells were suspended in an extraction buffer (10 mM Tris [pH 7.4], 150 mM NaCl, 0.05 mM spermidine, 0.125 mM spermine, 0.5 mM EDTA, 0.5 mM DTT, and 0.1% digitonin) with a protease inhibitor cocktail (Sigma, P8340), incubated on ice for 5 min, and centrifuged at 16,100 × *g* for 5 min. The supernatant was harvested and used for immunoprecipitation with the Protein G Dynabeads and antibodies. SDS-PAGE was carried out using 4%–15% Mini-PROTEAN TGX precast gels (Bio-Rad), and proteins were transferred onto membrane. Membrane was blocked with 1% BSA and then used for immunoblotting. Antibodies used in immunoprecipitation and immunoblotting are shown in Table S4.

Microscopy and Image Quantification

Cell images were acquired on an Axio Observer.Z1 (Zeiss) equipped with an LSM700 scanning module and an Objective Plan-Apochromat 63×/1.46 oil lens (Zeiss) using ZEN 2009 software (Zeiss). For image quantification, z-stack images were acquired with a spacing of 0.22 μm. Maximum-intensity projection of obtained slices was produced with the ZEN software, and ImageJ (NIH) software was used for image quantification.

SUPPLEMENTAL INFORMATION

Supplemental Information includes seven figures and five tables and can be found with this article online at http://dx.doi.org/10.1016/j.devcel.2016.05.006.

AUTHOR CONTRIBUTIONS

J.O., N.S., K.O., N.M.C.M., and K.K. conducted experiments; H.K. and T.N. produced antibodies and DNA clones, respectively; J.O., V.L., W.C.E., and H.M. wrote the manuscript.

ACKNOWLEDGMENTS

We would like to thank Hiroko Shinohara, Tomoe Hayashi, and Kazuko Yamada for their technical assistance, Koei Okazaki for discussion, and Naohito Nozaki for producing the antibodies. This work was supported by MEXT KAKENHI grants 23247030, 23114008, 16H04747, and 16H01414, as well as the Kazusa DNA Research Institute Foundation (H.M.). Work in the W.C.E. laboratory is funded by the Wellcome Trust, of which W.C.E. is a Principal Research Fellow (grant no. 073915). Additional experiments were supported by grants-in-aid from the Japan Society for the Promotion of Science and the Ministry of Education, Culture, Sports, Science and Technology of Japan (to H.K.) and the intramural research program of the NIH, National Cancer Institute, and Center for Cancer Research (to V.L.).

Received: October 9, 2015
Revised: April 8, 2016
Accepted: May 9, 2016
Published: June 6, 2016

REFERENCES

Allis, C.D., Berger, S.L., Cote, J., Dent, S., Jenuwien, T., Kouzarides, T., Pillus, L., Reinberg, D., Shi, Y., Shiekhattar, R., et al. (2007). New nomenclature for chromatin-modifying enzymes. Cell *131*, 633–636.

Allshire, R.C., and Karpen, G.H. (2008). Epigenetic regulation of centromeric chromatin: old dogs, new tricks? Nat. Rev. Genet. *9*, 923–937.

Allshire, R.C., Javerzat, J.P., Redhead, N.J., and Cranston, G. (1994). Position effect variegation at fission yeast centromeres. Cell *76*, 157–169.

Basilico, F., Maffini, S., Weir, J.R., Prumbaum, D., Rojas, A.M., Zimniak, T., De Antoni, A., Jeganathan, S., Voss, B., van Gerwen, S., et al. (2014). The pseudo GTPase CENP-M drives human kinetochore assembly. Elife *3*, e02978.

Bergmann, J.H., Martins, N.M., Larionov, V., Masumoto, H., and Earnshaw, W.C. (2012). HACking the centromere chromatin code: insights from human artificial chromosomes. Chromosome Res. *20*, 505–519.

Black, B.E., and Cleveland, D.W. (2011). Epigenetic centromere propagation and the nature of CENP-a nucleosomes. Cell *144*, 471–479.

Cardinale, S., Bergmann, J.H., Kelly, D., Nakano, M., Valdivia, M.M., Kimura, H., Masumoto, H., Larionov, V., and Earnshaw, W.C. (2009). Hierarchical inactivation of a synthetic human kinetochore by a chromatin modifier. Mol. Biol. Cell *20*, 4194–4204.

Carroll, C.W., Milks, K.J., and Straight, A.F. (2010). Dual recognition of CENP-A nucleosomes is required for centromere assembly. J. Cell Biol. *189*, 1143–1155.

Catania, S., Pidoux, A.L., and Allshire, R.C. (2015). Sequence features and transcriptional stalling within centromere DNA promote establishment of CENP-A chromatin. PLoS Genet. *11*, e1004986.

Cheeseman, I.M., and Desai, A. (2008). Molecular architecture of the kinetochore-microtubule interface. Nat. Rev. Mol. Cell Biol. *9*, 33–46.

Chen, C.C., Bowers, S., Lipinszki, Z., Palladino, J., Trusiak, S., Bettini, E., Rosin, L., Przewloka, M.R., Glover, D.M., O'Neill, R.J., and Mellone, B.G. (2015). Establishment of centromeric chromatin by the CENP-A assembly factor CAL1 requires FACT-mediated transcription. Dev. Cell *34*, 73–84.

Cleveland, D.W., Mao, Y., and Sullivan, K.F. (2003). Centromeres and kinetochores: from epigenetics to mitotic checkpoint signaling. Cell *112*, 407–421.

Dambacher, S., Deng, W., Hahn, M., Sadic, D., Fröhlich, J., Nuber, A., Hoischen, C., Diekmann, S., Leonhardt, H., and Schotta, G. (2012). CENP-C facilitates the recruitment of M18BP1 to centromeric chromatin. Nucleus *3*, 101–110.

Doyon, Y., Cayrou, C., Ullah, M., Landry, A.J., Côté, V., Selleck, W., Lane, W.S., Tan, S., Yang, X.J., and Côté, J. (2006). ING tumor suppressor proteins are critical regulators of chromatin acetylation required for genome expression and perpetuation. Mol. Cell *21*, 51–64.

Dunleavy, E.M., Roche, D., Tagami, H., Lacoste, N., Ray-Gallet, D., Nakamura, Y., Daigo, Y., Nakatani, Y., and Almouzni-Pettinotti, G. (2009). HJURP is a cell-cycle-dependent maintenance and deposition factor of CENP-A at centromeres. Cell *137*, 485–497.

Earnshaw, W.C., and Rothfield, N. (1985). Identification of a family of human centromere proteins using autoimmune sera from patients with scleroderma. Chromosoma *91*, 313–321.

Earnshaw, W.C., Ratrie, H., 3rd, and Stetten, G. (1989). Visualization of centromere proteins CENP-B and CENP-C on a stable dicentric chromosome in cytological spreads. Chromosoma *98*, 1–12.

Fachinetti, D., Folco, H.D., Nechemia-Arbely, Y., Valente, L.P., Nguyen, K., Wong, A.J., Zhu, Q., Holland, A.J., Desai, A., Jansen, L.E., et al. (2013). A two-step mechanism for epigenetic specification of centromere identity and function. Nat. Cell Biol. *15*, 1056–1066.

Fachinetti, D., Han, J.S., McMahon, M.A., Ly, P., Abdullah, A., Wong, A.J., and Cleveland, D.W. (2015). DNA sequence-specific binding of CENP-B enhances the fidelity of human centromere function. Dev. Cell *33*, 314–327.

Foltz, D.R., Jansen, L.E., Bailey, A.O., Yates, J.R., 3rd, Bassett, E.A., Wood, S., Black, B.E., and Cleveland, D.W. (2009). Centromere-specific assembly of CENP-a nucleosomes is mediated by HJURP. Cell *137*, 472–484.

Fujita, Y., Hayashi, T., Kiyomitsu, T., Toyoda, Y., Kokubu, A., Obuse, C., and Yanagida, M. (2007). Priming of centromere for CENP-A recruitment by human hMis18alpha, hMis18beta, and M18BP1. Dev. Cell *12*, 17–30.

Fujita, R., Otake, K., Arimura, Y., Horikoshi, N., Miya, Y., Shiga, T., Osakabe, A., Tachiwana, H., Ohzeki, J., Larionov, V., et al. (2015). Stable complex formation of CENP-B with the CENP-A nucleosome. Nucleic Acids Res. *43*, 4909–4922.

Fukagawa, T., and Earnshaw, W.C. (2014). The centromere: chromatin foundation for the kinetochore machinery. Dev. Cell *30*, 496–508.

Grewal, S.I., and Jia, S. (2007). Heterochromatin revisited. Nat. Rev. Genet. *8*, 35–46.

Harrington, J.J., Van Bokkelen, G., Mays, R.W., Gustashaw, K., and Willard, H.F. (1997). Formation of de novo centromeres and construction of first-generation human artificial microchromosomes. Nat. Genet. *15*, 345–355.

Iizuka, M., and Stillman, B. (1999). Histone acetyltransferase HBO1 interacts with the ORC1 subunit of the human initiator protein. J. Biol. Chem. *274*, 23027–23034.

Ikeno, M., Grimes, B., Okazaki, T., Nakano, M., Saitoh, K., Hoshino, H., McGill, N.I., Cooke, H., and Masumoto, H. (1998). Construction of YAC-based mammalian artificial chromosomes. Nat. Biotechnol. *16*, 431–439.

Jansen, L.E., Black, B.E., Foltz, D.R., and Cleveland, D.W. (2007). Propagation of centromeric chromatin requires exit from mitosis. J. Cell Biol. *176*, 795–805.

Kueh, A.J., Dixon, M.P., Voss, A.K., and Thomas, T. (2011). HBO1 is required for H3K14 acetylation and normal transcriptional activity during embryonic development. Mol. Cell Biol. *31*, 845–860.

Liu, B., Wang, Z., Zhang, L., Ghosh, S., Zheng, H., and Zhou, Z. (2013). Depleting the methyltransferase Suv39h1 improves DNA repair and extends lifespan in a progeria mouse model. Nat. Commun. *4*, 1868.

Loyola, A., LeRoy, G., Wang, Y.H., and Reinberg, D. (2001). Reconstitution of recombinant chromatin establishes a requirement for histone-tail modifications during chromatin assembly and transcription. Genes Dev. *15*, 2837–2851.

Maddox, P.S., Hyndman, F., Monen, J., Oegema, K., and Desai, A. (2007). Functional genomics identifies a Myb domain-containing protein family required for assembly of CENP-A chromatin. J. Cell Biol. *176*, 757–763.

Masumoto, H., Masukata, H., Muro, Y., Nozaki, N., and Okazaki, T. (1989). A human centromere antigen (CENP-B) interacts with a short specific sequence in alphoid DNA, a human centromeric satellite. J. Cell Biol. *109*, 1963–1973.

Martins, N.M., Bergmann, J.H., Shono, N., Kimura, H., Larionov, V., Masumoto, H., and Earnshaw, W.C. (2015). Epigenetic engineering shows that a human centromere resists silencing mediated by H3K27me3/K9me3. Mol. Biol. Cell *27*, 177–196.

McKinley, K.L., and Cheeseman, I.M. (2014). Polo-like kinase 1 licenses CENP-A deposition at centromeres. Cell *158*, 397–411.

Mellone, B.G., Ball, L., Suka, N., Grunstein, M.R., Partridge, J.F., and Allshire, R.C. (2003). Centromere silencing and function in fission yeast is governed by the amino terminus of histone H3. Curr. Biol. *13*, 1748–1757.

Miotto, B., and Struhl, K. (2008). HBO1 histone acetylase is a coactivator of the replication licensing factor Cdt1. Genes Dev. *22*, 2633–2638.

Miotto, B., and Struhl, K. (2010). HBO1 histone acetylase activity is essential for DNA replication licensing and inhibited by Geminin. Mol. Cell *37*, 57–66.

Moree, B., Meyer, C.B., Fuller, C.J., and Straight, A.F. (2011). CENP-C recruits M18BP1 to centromeres to promote CENP-A chromatin assembly. J. Cell Biol. *194*, 855–871.

Muller, H.J. (1930). Types of visible variations induced by X-rays in Drosophila. J. Genet. *22*, 299–344.

Nakano, M., Cardinale, S., Noskov, V.N., Gassmann, R., Vagnarelli, P., Kandels-Lewis, S., Larionov, V., Earnshaw, W.C., and Masumoto, H. (2008). Inactivation of a human kinetochore by specific targeting of chromatin modifiers. Dev. Cell *14*, 507–522.

Nakayama, T., Nishioka, K., Dong, Y.X., Shimojima, T., and Hirose, S. (2007). *Drosophila* GAGA factor directs histone H3.3 replacement that prevents the heterochromatin spreading. Genes Dev. *21*, 552–561.

Ohzeki, J., Nakano, M., Okada, T., and Masumoto, H. (2002). CENP-B box is required for de novo centromere chromatin assembly on human alphoid DNA. J. Cell Biol. *159*, 765–775.

Ohzeki, J., Bergmann, J.H., Kouprina, N., Noskov, V.N., Nakano, M., Kimura, H., Earnshaw, W.C., Larionov, V., and Masumoto, H. (2012). Breaking the HAC barrier: histone H3K9 acetyl/methyl balance regulates CENP-A assembly. EMBO J. *31*, 2391–2402.

Ohzeki, J., Larionov, V., Earnshaw, W.C., and Masumoto, H. (2015). Genetic and epigenetic regulation of centromeres: a look at HAC formation. Chromosome Res. *23*, 87–103.

Okada, T., Ohzeki, J., Nakano, M., Yoda, K., Brinkley, W.R., Larionov, V., and Masumoto, H. (2007). CENP-B controls centromere formation depending on the chromatin context. Cell *131*, 1287–1300.

Perpelescu, M., Nozaki, N., Obuse, C., Yang, H., and Yoda, K. (2009). Active establishment of centromeric CENP-A chromatin by RSF complex. J. Cell Biol. *185*, 397–407.

Peters, A.H., O'Carroll, D., Scherthan, H., Mechtler, K., Sauer, S., Schöfer, C., Weipoltshammer, K., Pagani, M., Lachner, M., Kohlmaier, A., et al. (2001). Loss of the Suv39h histone methyltransferases impairs mammalian heterochromatin and genome stability. Cell *107*, 323–337.

Reddy, B.D., Wang, Y., Niu, L., Higuchi, E.C., Marguerat, S.B., Bähler, J., Smith, G.R., and Jia, S. (2011). Elimination of a specific histone H3K14 acetyltransferase complex bypasses the RNAi pathway to regulate pericentric heterochromatin functions. Genes Dev. *25*, 214–219.

Ribeiro, S.A., Vagnarelli, P., Dong, Y., Hori, T., McEwen, B.F., Fukagawa, T., Flors, C., and Earnshaw, W.C. (2010). A super-resolution map of the vertebrate kinetochore. Proc. Natl. Acad. Sci. USA *107*, 10484–10489.

Scott, K.C., Merrett, S.L., and Willard, H.F. (2006). A heterochromatin barrier partitions the fission yeast centromere into discrete chromatin domains. Curr. Biol. *16*, 119–129.

Shono, N., Ohzeki, J., Otake, K., Martins, N.M., Nagase, T., Kimura, H., Larionov, V., Earnshaw, W.C., and Masumoto, H. (2015). CENP-C and CENP-I are key connecting factors for kinetochore and CENP-A assembly. J. Cell Sci. *128*, 4572–4587.

Sullivan, B.A., and Karpen, G.H. (2004). Centromeric chromatin exhibits a histone modification pattern that is distinct from both euchromatin and heterochromatin. Nat. Struct. Mol. Biol. *11*, 1076–1083.

Suzuki, N., Nakano, M., Nozaki, N., Egashira, S., Okazaki, T., and Masumoto, H. (2004). CENP-B interacts with CENP-C domains containing Mif2 regions responsible for centromere localization. J. Biol. Chem. *279*, 5934–5946.

Szenker, E., Ray-Gallet, D., and Almouzni, G. (2011). The double face of the histone variant H3.3. Cell Res. *21*, 421–434.

Talbert, P.B., and Henikoff, S. (2006). Spreading of silent chromatin: inaction at a distance. Nat. Rev. Genet. *7*, 793–803.

Wang, J., Reddy, B.D., and Jia, S. (2015). Rapid epigenetic adaptation to uncontrolled heterochromatin spreading. Elife *4*, e06179.

Willard, H.F., and Waye, J.S. (1987). Hierarchical order in chromosome-specific human alpha satellite DNA. Trends Genet. *3*, 192–198.

Zhang, W., Li, J., Suzuki, K., Qu, J., Wang, P., Zhou, J., Liu, X., Ren, R., Xu, X., Ocampo, A., et al. (2015). Aging stem cells. A Werner syndrome stem cell model unveils heterochromatin alterations as a driver of human aging. Science *348*, 1160–1163.

Developmental Cell, Volume *37*

Supplemental Information

KAT7/HBO1/MYST2 Regulates CENP-A Chromatin Assembly by Antagonizing Suv39h1-Mediated Centromere Inactivation

Jun-ichirou Ohzeki, Nobuaki Shono, Koichiro Otake, Nuno M.C. Martins, Kazuto Kugou, Hiroshi Kimura, Takahiro Nagase, Vladimir Larionov, William C. Earnshaw, and Hiroshi Masumoto

Supplemental Inventory

Supplemental Figure S1, Related to Figure 1.

Supplemental Figure S2, Related to Figure 2.

Supplemental Figure S3, Related to Figure 3.

Supplemental Figure S4, Related to Figure 4.

Supplemental Figure S5, Related to Figure 5.

Supplemental Figure S6, Related to Figure 6.

Supplemental Figure S7, Related to Figure 7.

Supplemental Figure legends

Supplemental Table S1, Related to Experimental Procedure.

Supplemental Table S2, Related to Experimental Procedure.

Supplemental Table S3, Related to Experimental Procedure.

Supplemental Table S4, Related to Experimental Procedure.

Supplemental Table S5, Related to Experimental Procedure.

Supplemental References

Figure S1

A

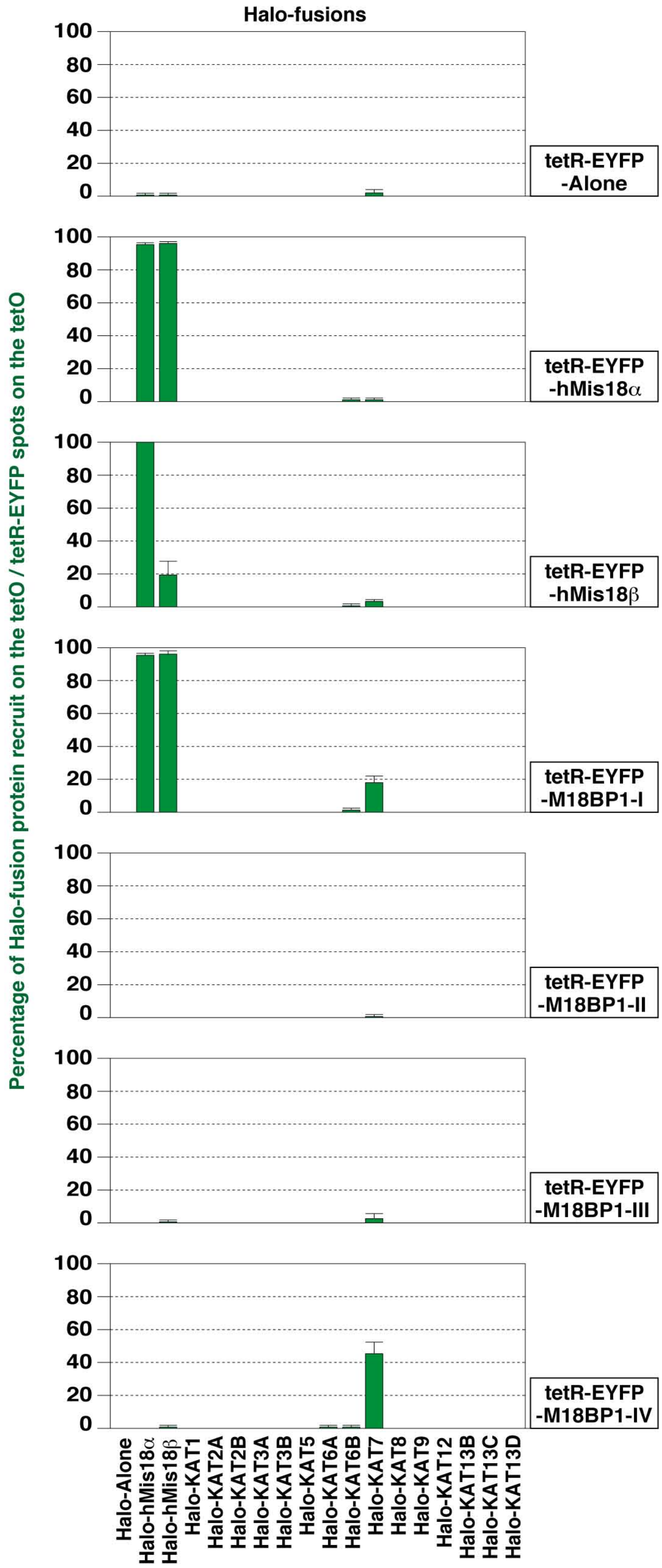

B

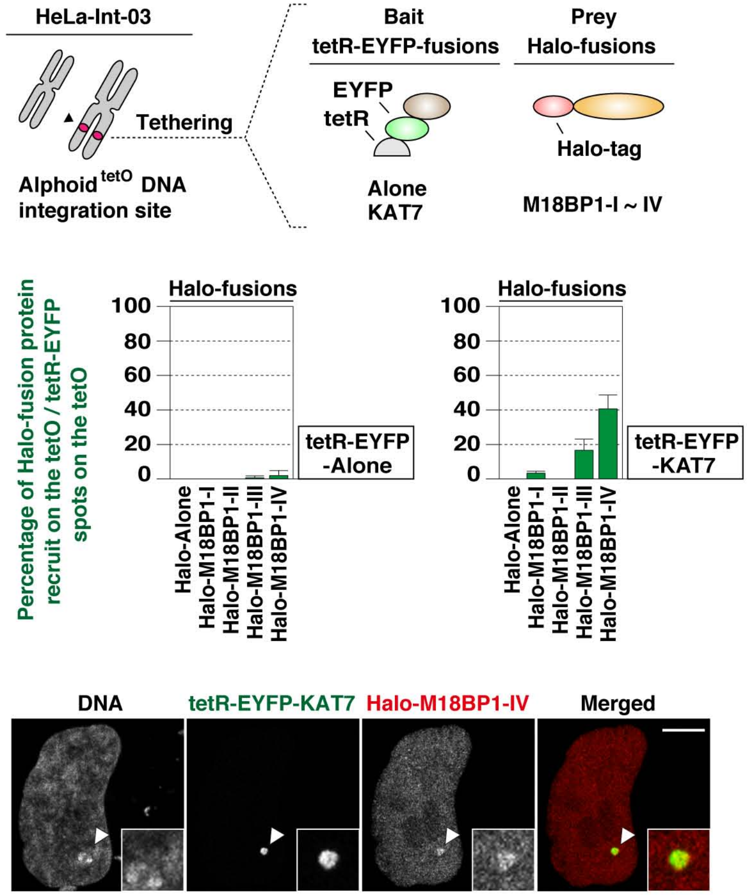

C

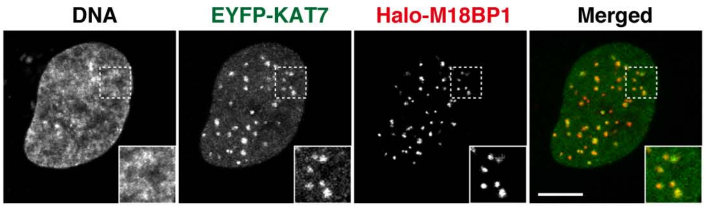

Figure S2

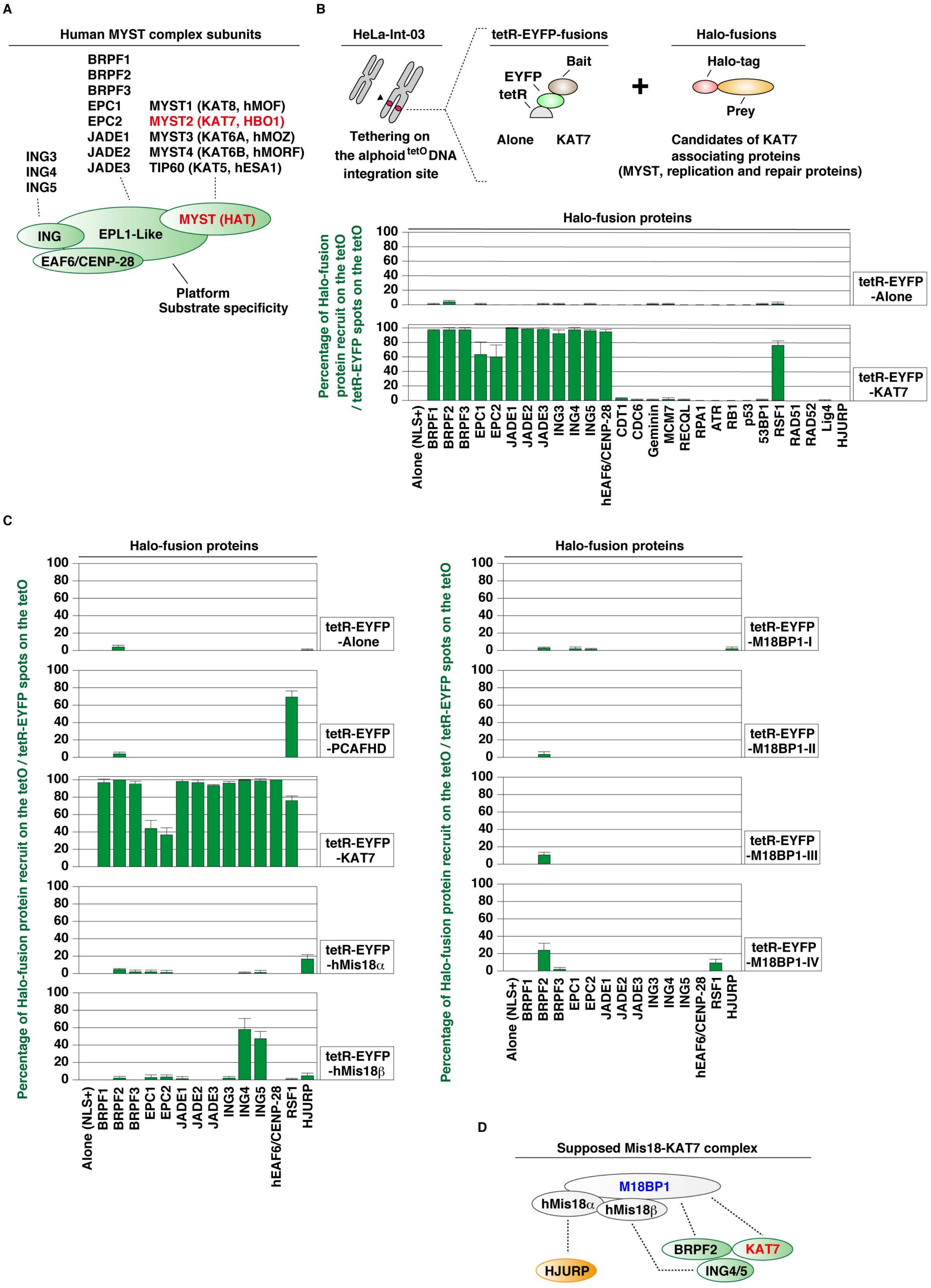

A
Human MYST complex subunits
BRPF1
BRPF2
BRPF3
EPC1
EPC2
JADE1
JADE2
JADE3
MYST1 (KAT8, hMOF)
MYST2 (KAT7, HBO1)
MYST3 (KAT6A, hMOZ)
MYST4 (KAT6B, hMORF)
TIP60 (KAT5, hESA1)
ING3
ING4
ING5
MYST (HAT)
ING
EPL1-Like
EAF6/CENP-28
Platform
Substrate specificity
B
HeLa-Int-03
Tethering on the alphoid tetO DNA integration site
tetR-EYFP-fusions
Bait
EYFP
tetR
Alone
KAT7
Halo-fusions
Halo-tag
Prey
Candidates of KAT7 associating proteins
(MYST, replication and repair proteins)
Halo-fusion proteins
Percentage of Halo-fusion protein recruit on the tetO / tetR-EYFP spots on the tetO
tetR-EYFP -Alone
tetR-EYFP -KAT7
Alone (NLS+)
BRPF1
BRPF2
BRPF3
EPC1
EPC2
JADE1
JADE2
JADE3
ING3
ING4
ING5
hEAF6/CENP-28
CDT1
CDC6
Geminin
MCM7
RECQL
RPA1
ATR
RB1
p53
53BP1
RSF1
RAD51
RAD52
Lig4
HJURP
C
Halo-fusion proteins
Percentage of Halo-fusion protein recruit on the tetO / tetR-EYFP spots on the tetO
tetR-EYFP -Alone
tetR-EYFP -PCAFHD
tetR-EYFP -KAT7
tetR-EYFP -hMis18α
tetR-EYFP -hMis18β
tetR-EYFP -M18BP1-I
tetR-EYFP -M18BP1-II
tetR-EYFP -M18BP1-III
tetR-EYFP -M18BP1-IV
Alone (NLS+)
BRPF1
BRPF2
BRPF3
EPC1
EPC2
JADE1
JADE2
JADE3
ING3
ING4
ING5
hEAF6/CENP-28
RSF1
HJURP
D
Supposed Mis18-KAT7 complex
M18BP1
hMis18α
hMis18β
BRPF2
KAT7
HJURP
ING4/5

Figure S3

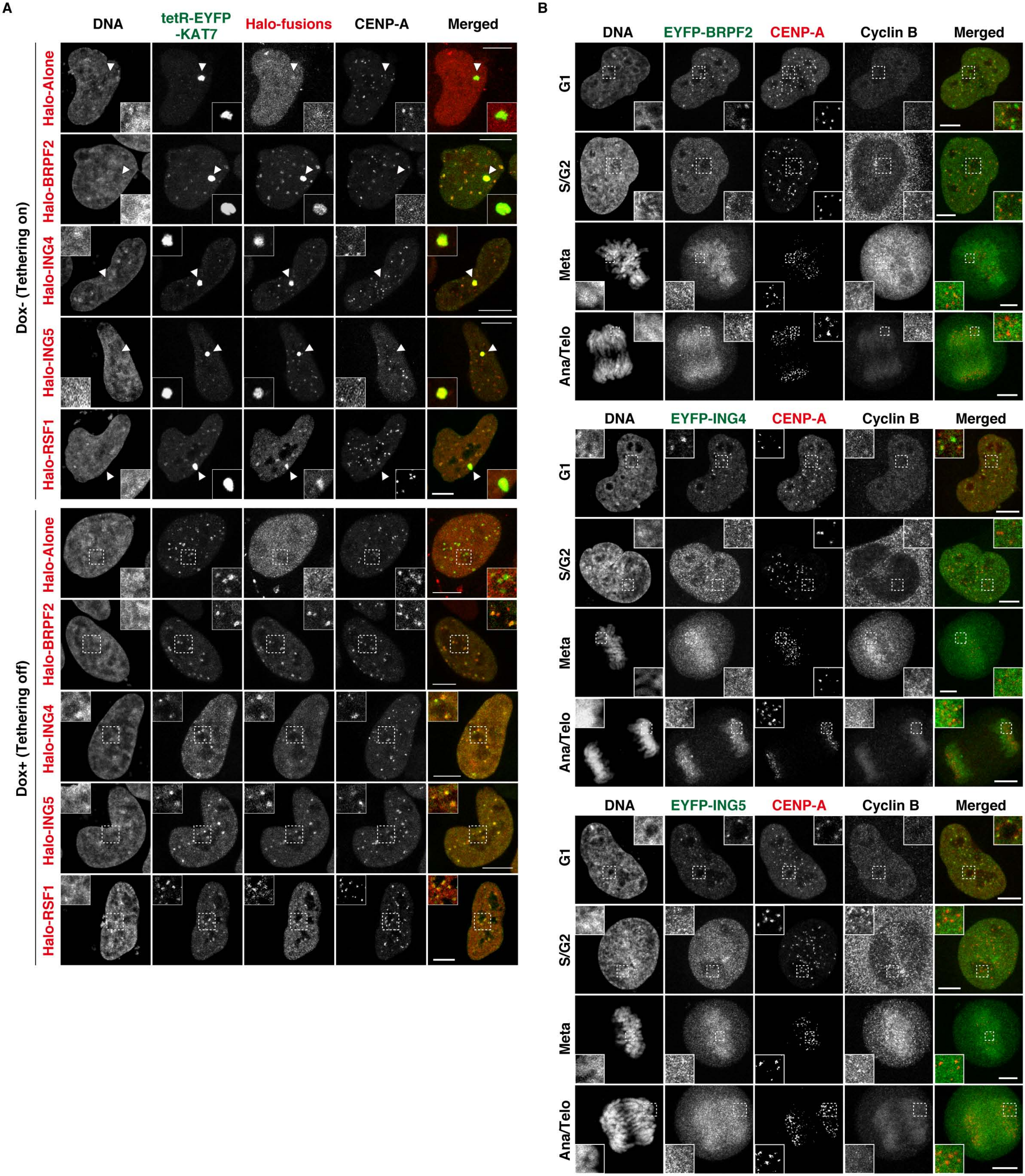

A
DNA
tetR-EYFP -KAT7
Halo-fusions
CENP-A
Merged
Dox- (Tethering on)
Halo-Alone
Halo-BRPF2
Halo-ING4
Halo-ING5
Halo-RSF1
Dox+ (Tethering off)
Halo-Alone
Halo-BRPF2
Halo-ING4
Halo-ING5
Halo-RSF1
B
DNA
EYFP-BRPF2
CENP-A
Cyclin B
Merged
G1
S/G2
Meta
Ana/Telo
DNA
EYFP-ING4
CENP-A
Cyclin B
Merged
G1
S/G2
Meta
Ana/Telo
DNA
EYFP-ING5
CENP-A
Cyclin B
Merged
G1
S/G2
Meta
Ana/Telo

Figure S4

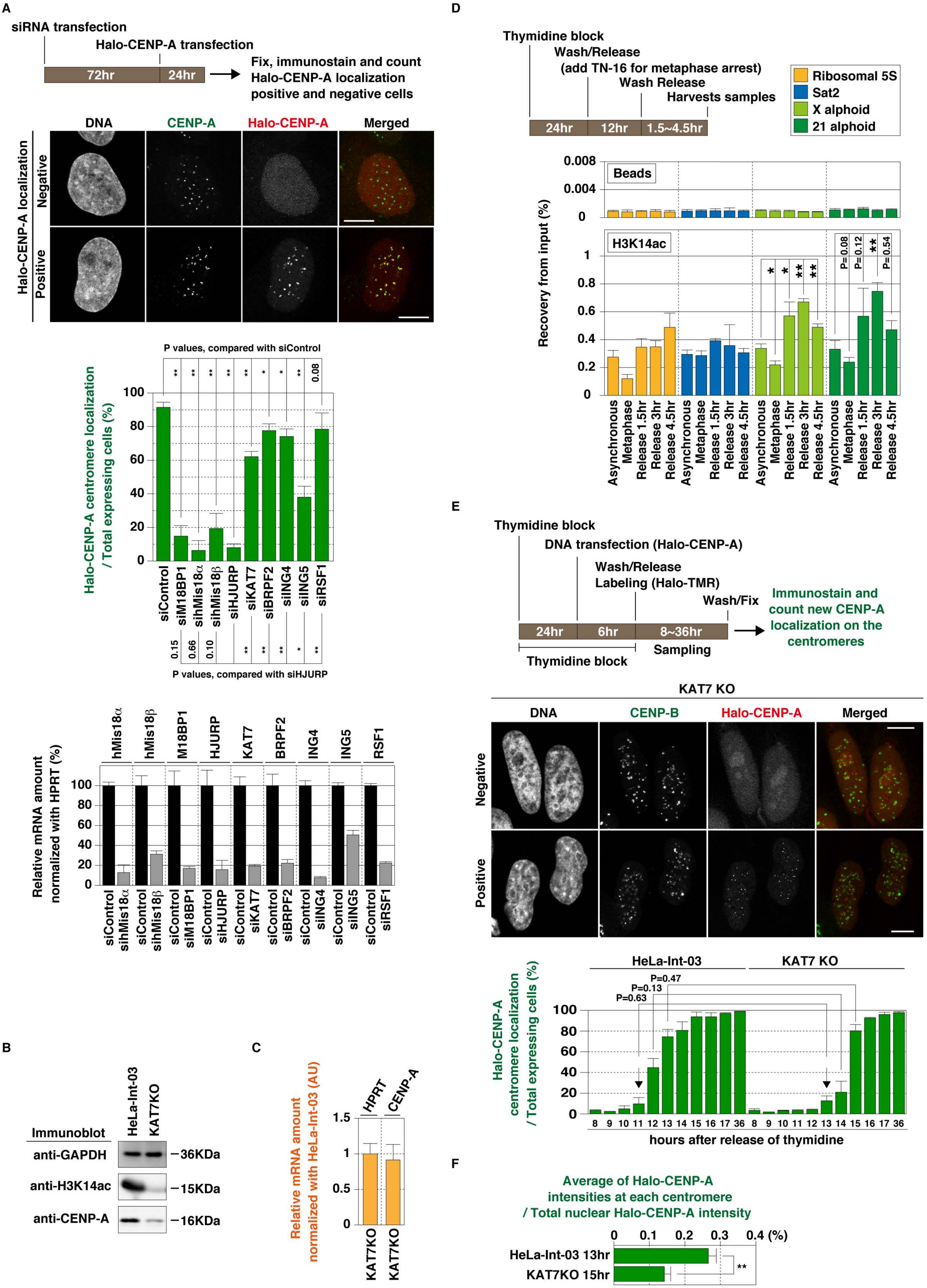
A
siRNA transfection
Halo-CENP-A transfection
72hr
24hr
Fix, immunostain and count Halo-CENP-A localization positive and negative cells
DNA
CENP-A
Halo-CENP-A
Merged
Halo-CENP-A localization
Negative
Positive
P values, compared with siControl
Halo-CENP-A centromere localization / Total expressing cells (%)
siControl
siM18BP1
sihMis18α
sihMis18β
siHJURP
siKAT7
siBRPF2
siING4
siING5
siRSF1
P values, compared with siHJURP
Relative mRNA amount normalized with HPRT (%)
hMis18α
hMis18β
M18BP1
HJURP
KAT7
BRPF2
ING4
ING5
RSF1
B
HeLa-Int-03
KAT7KO
Immunoblot
anti-GAPDH
anti-H3K14ac
anti-CENP-A
−36KDa
−15KDa
−16KDa
C
Relative mRNA amount normalized with HeLa-Int-03 (AU)
HPRT
CENP-A
KAT7KO
D
Thymidine block
Wash/Release
(add TN-16 for metaphase arrest)
Wash Release
Harvests samples
24hr
12hr
1.5~4.5hr
Ribosomal 5S
Sat2
X alphoid
21 alphoid
Beads
H3K14ac
Recovery from input (%)
P=0.08
P=0.12
P=0.54
Asynchronous
Metaphase
Release 1.5hr
Release 3hr
Release 4.5hr
E
Thymidine block
DNA transfection (Halo-CENP-A)
Wash/Release
Labeling (Halo-TMR)
Wash/Fix
24hr
6hr
8~36hr
Thymidine block
Sampling
Immunostain and count new CENP-A localization on the centromeres
KAT7 KO
DNA
CENP-B
Halo-CENP-A
Merged
Negative
Positive
HeLa-Int-03
KAT7 KO
P=0.47
P=0.13
P=0.63
Halo-CENP-A centromere localization / Total expressing cells (%)
hours after release of thymidine
F
Average of Halo-CENP-A intensities at each centromere / Total nuclear Halo-CENP-A intensity
0 0.1 0.2 0.3 0.4 (%)
HeLa-Int-03 13hr
KAT7KO 15hr

Figure S5

A

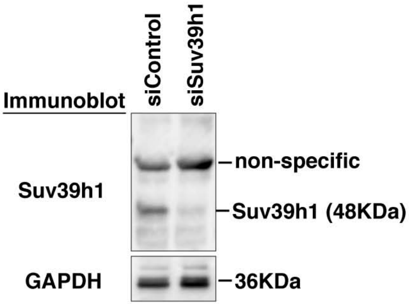

Immunoblot
siControl
siSuv39h1
Suv39h1
non-specific
Suv39h1 (48KDa)
GAPDH
36KDa

B

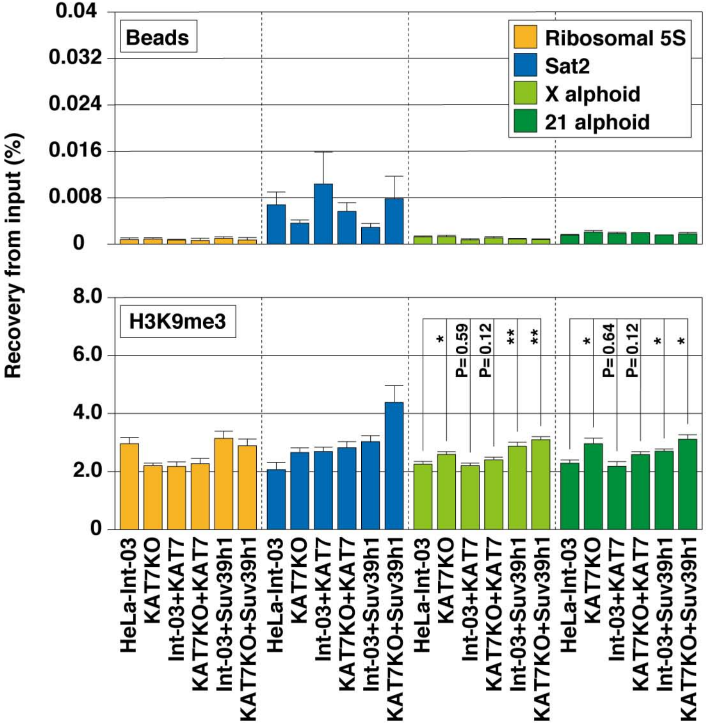

Recovery from input (%)
Beads
Ribosomal 5S
Sat2
X alphoid
21 alphoid
0.04
0.032
0.024
0.016
0.008
0
H3K9me3
8.0
6.0
4.0
2.0
0
*
P=0.59
P=0.12
**
**
*
P=0.64
P=0.12
*
*
HeLa-Int-03
KAT7KO
Int-03+KAT7
KAT7KO+KAT7
Int-03+Suv39h1
KAT7KO+Suv39h1

Figure S6

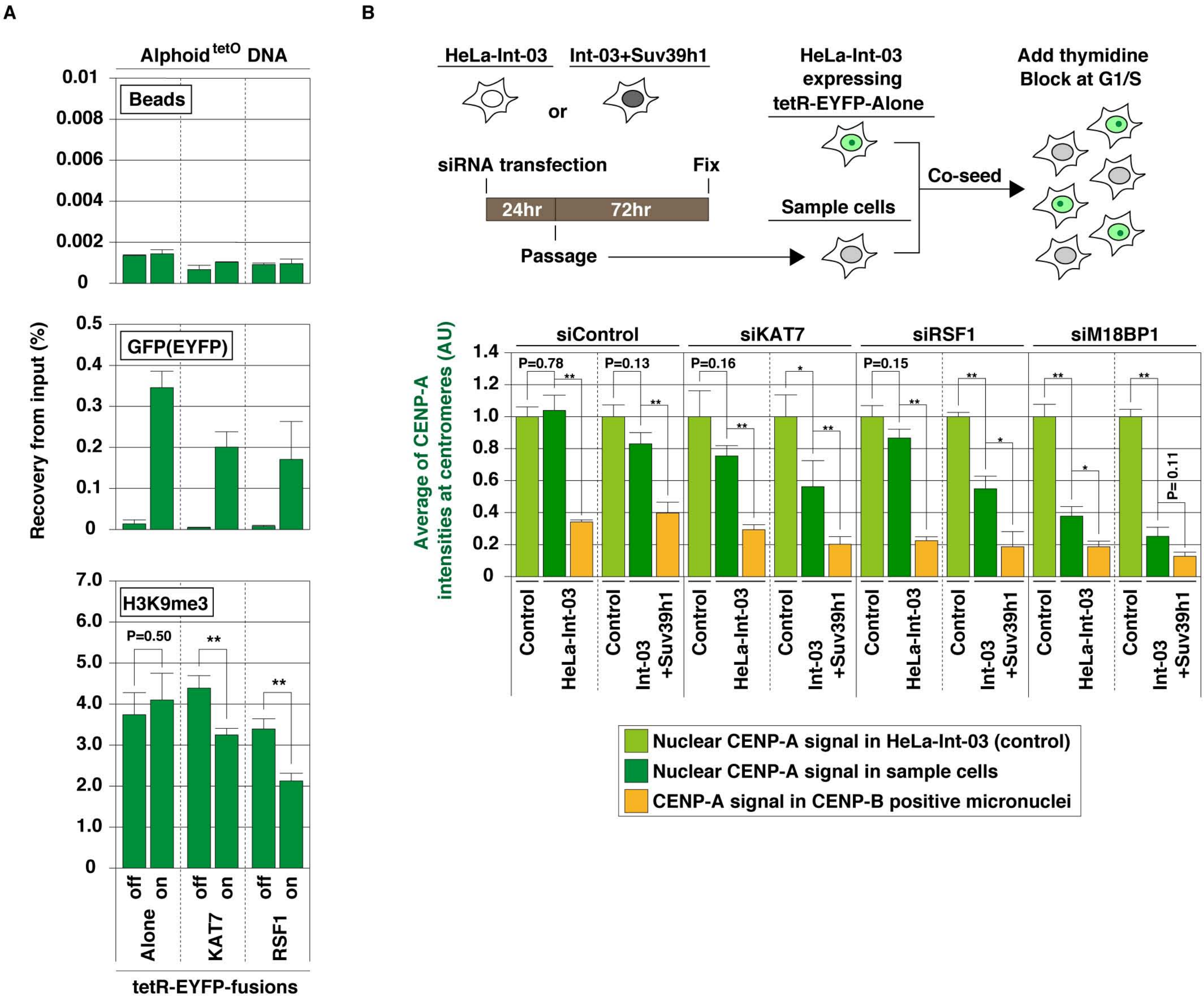
A
Alphoid tetO DNA
Beads
GFP(EYFP)
H3K9me3
Recovery from input (%)
P=0.50
**
off
on
Alone
KAT7
RSF1
tetR-EYFP-fusions
B
HeLa-Int-03
or
Int-03+Suv39h1
siRNA transfection
Fix
24hr
72hr
Passage
HeLa-Int-03 expressing tetR-EYFP-Alone
Sample cells
Co-seed
Add thymidine Block at G1/S
siControl
siKAT7
siRSF1
siM18BP1
Average of CENP-A intensities at centromeres (AU)
P=0.78
P=0.13
P=0.16
P=0.15
P=0.11
Control
HeLa-Int-03
Int-03 +Suv39h1
Nuclear CENP-A signal in HeLa-Int-03 (control)
Nuclear CENP-A signal in sample cells
CENP-A signal in CENP-B positive micronuclei

Figure S7

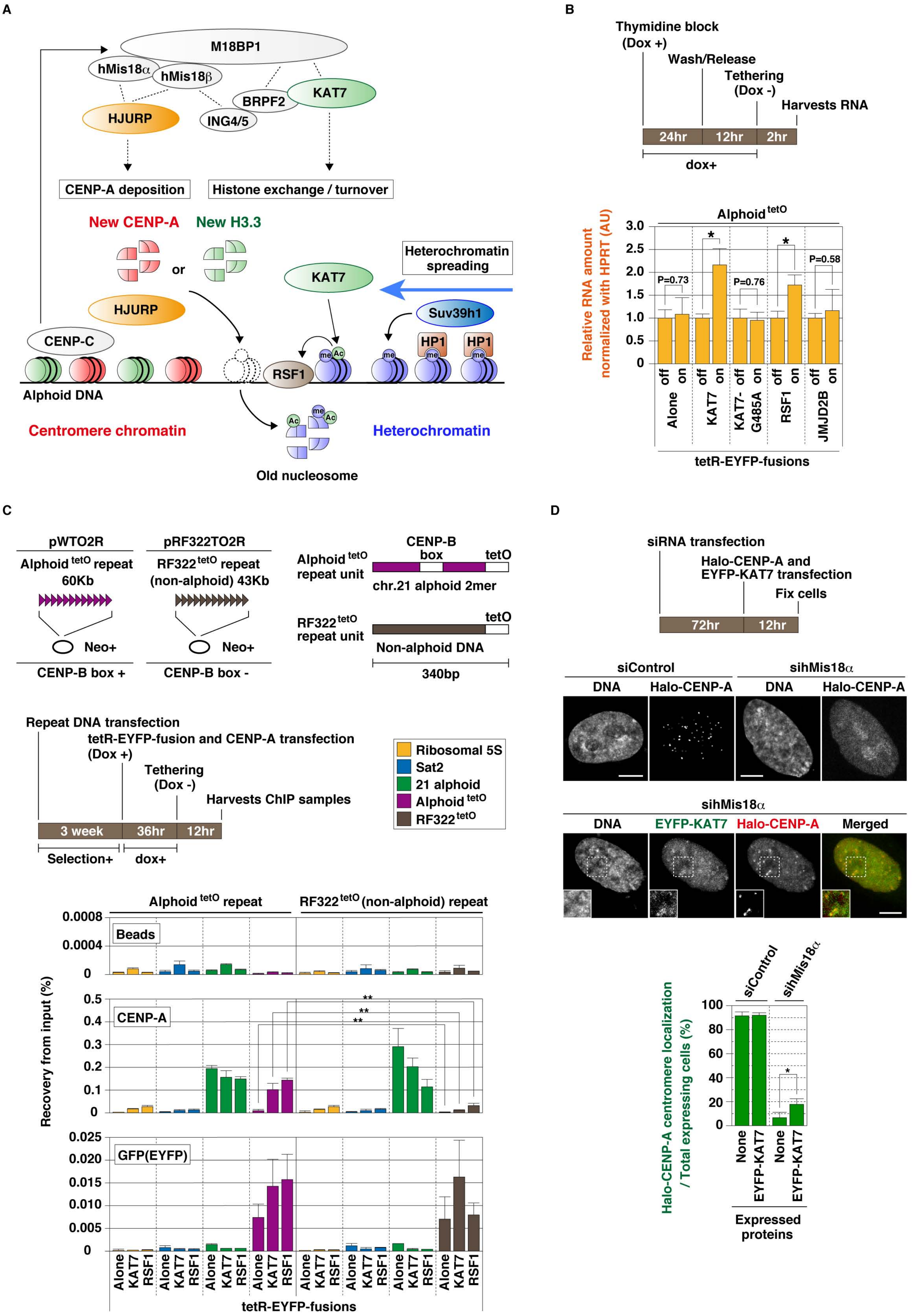
A
M18BP1
hMis18α
hMis18β
BRPF2
KAT7
HJURP
ING4/5
CENP-A deposition
Histone exchange / turnover
New CENP-A
New H3.3
or
Heterochromatin spreading
KAT7
HJURP
Suv39h1
CENP-C
HP1
HP1
me
Ac
RSF1
Alphoid DNA
Centromere chromatin
Heterochromatin
Old nucleosome
B
Thymidine block (Dox +)
Wash/Release
Tethering (Dox -)
Harvests RNA
24hr
12hr
2hr
dox+
Alphoid tetO
Relative RNA amount normalized with HPRT (AU)
P=0.73
P=0.76
P=0.58
off on
Alone
KAT7
KAT7-G485A
RSF1
JMJD2B
tetR-EYFP-fusions
C
pWTO2R
pRF322TO2R
Alphoid tetO repeat 60Kb
RF322 tetO repeat (non-alphoid) 43Kb
Neo+
CENP-B box +
CENP-B box -
Alphoid tetO repeat unit
CENP-B box
tetO
chr.21 alphoid 2mer
RF322 tetO repeat unit
Non-alphoid DNA
340bp
Repeat DNA transfection
tetR-EYFP-fusion and CENP-A transfection (Dox +)
Tethering (Dox -)
Harvests ChIP samples
3 week
36hr
12hr
Selection+
dox+
Ribosomal 5S
Sat2
21 alphoid
Alphoid tetO
RF322 tetO
Alphoid tetO repeat
RF322 tetO (non-alphoid) repeat
Beads
CENP-A
GFP(EYFP)
Recovery from input (%)
Alone KAT7 RSF1
tetR-EYFP-fusions
D
siRNA transfection
Halo-CENP-A and EYFP-KAT7 transfection
Fix cells
72hr
12hr
siControl
sihMis18α
DNA
Halo-CENP-A
EYFP-KAT7
Merged
Halo-CENP-A centromere localization / Total expressing cells (%)
None
EYFP-KAT7
Expressed proteins

Supplemental Figure Legends

Figure S1. Supplemental information for Figure 1, Related to Figure 1

(A) Graphical representation of the data in Figure 1C. Data are presented as mean + s.d. (n=3). (B) M18BP1 recruitment with KAT7 tethering. (Top) Schema for the experiments. (Middle) The observed frequency of recruitment of Halo-fusion proteins by tetR-EYFP-fusion protein tethering (% of cells that have a detectable signal) was plotted (>50 tetR-EYFP spots were counted in each sample). Data are presented as mean + s.d. (n=3). (Bottom) Fluorescence images were obtained by DAPI staining (DNA), EYFP (green), and Halo-TMR-ligand (red). White arrowheads indicate the loci of tetR-EYFP-fusion protein spots on the alphoid^{tetO} DNA integration site. (C) Co-localization of EYFP-KAT7 and Halo-M18BP1. Fluorescence images were obtained by DAPI staining (DNA), EYFP (green), and Halo-TMR-ligand (red). Scale bars = 5 μm.

Figure S2. Screening of KAT7-associating proteins, Related to Figure 2

(A) Scheme of the human MYST complexes formed on the EPL1-like motif-containing platform proteins. The MYST domain-containing HAT proteins interact with this EPL1-like motif. Inhibitor of growth (ING) subunits and EAF6/CENP-28 proteins are also components of the MYST complexes (Doyon et al., 2006; Ohta et al., 2010; Avvakumov et al., 2012). Most of the known MYST complexes are heterotetramers of the MYST domain HAT, ING subunit, EAF6/CENP-28, and EPL1-like platform proteins. In addition, KAT7 and BRPF2 heterodimer has been reported (Mishima et al., 2011). (B) Scheme of screening KAT7-associating proteins. TetR-EYFP-Alone or -KAT7 proteins were tethered to the alphoid^{tetO} DNA integration site as "bait" and recruitment of Halo-tag-fused "prey" proteins tested. The Halo-fused genes were related to known

KAT7 functions, DNA replication, repair, and putative KAT7-interacting proteins. The frequency of recruitment of Halo-fusion proteins by tetR-EYFP-fusion protein tethering (% of cells that have a detectable signal) was plotted. Data are presented as mean + s.d. (n≥2). (C) A graphical representation of the data in Figure 2A heat map. Data are presented as mean + s.d. (n≥2). (D) A conceptual diagram of a hypothetical Mis18-KAT7 protein complex assumed from the results in Figure 2A.

Figure S3. Localizations of Mis18-KAT7 relating proteins, Related to Figure 3

(A) Localizations of tetR-EYFP-KAT7 and Halo-fused proteins in the presence or absence of doxycycline. Fluorescence images were obtained with DAPI (DNA), EYFP (green) and Halo-TMR-ligand (red). White arrowheads indicate the presence of tetR-EYFP-fusion protein spots on the alphoid^{tetO} DNA integration site. (B) Localization of EYFP-BRPF2, -ING4 and -ING5 during the cell cycle. Fluorescence images were obtained with DAPI (DNA), EYFP (green), anti-CENP-A antibody (red), and anti-cyclin B antibody (far red: shown in gray). Cell cycles were distinguished by images of DAPI and anti-cyclin B staining. Scale bars = 5 μm. See also Figure 3A.

Figure S4. Supplemental information for Figure 4, Related to Figure 4

(A) Halo-CENP-A localization at the centromere following depletion of Mis18 and KAT7-related proteins. (Top) Scheme of the depletion experiment and examples of Halo-CENP-A localization (siING5). Fluorescence images were obtained with DAPI (DNA), anti-CENP-A antibody (green), and Halo-TMR-Ligand (red). (Middle) The centromere localization frequency of Halo-CENP-A is plotted (>200 cells were counted in each sample). Data are presented as mean + s.d. (n=3). *P<0.05, **P<0.005 (t-test). (Bottom) Relative mRNA levels. Cells were transfected with siRNA and total RNA was

purified after 3 days. The total RNA was reverse transcribed and quantitated by qPCR. Quantitated target mRNA amounts were normalized to *HPRT* mRNA and plotted. The primer sets used are indicated at the top of the graph. The transfected siRNAs are indicated at the bottom of the graph. Data are presented as mean + s.d. (n=3). (B) H3K14ac and CENP-A immunoblotting using total cell lysate. GAPDH was used as a loading control. (C) RT-PCR quantitation of mRNA levels. Data are presented as mean + s.d. (n=3). (D) Time course ChIP analysis. Cells were harvested at each time point and fixed for ChIP analysis. ChIP was carried out with anti-H3K14ac antibody or beads alone (no antibody) as a control. Data are presented as mean + s.d. (n=3). *P<0.05, **P<0.005 (t-test). (E) (Top) Scheme of detecting a new CENP-A localization at the centromere. Cells were synchronized at G1/S using thymidine and then transfected with the vector expressing Halo-CENP-A. Cells were fixed at each time point after release of the thymidine block. (Middle) Fluorescence images were obtained with DAPI (DNA), anti-CENP-B antibody (green), and Halo-TMR-ligand (red). Scale bars = 5 μm. (Bottom) Centromere localization frequency of Halo-CENP-A (>200 cells were counted in each sample). Black arrows indicate a time point when >50% of cells exit mitosis. Data are presented as mean + s.d. (n=2). (F) Average of Halo-CENP-A intensity at each centromere. From the panel E experiments, cell samples from a time point when >50% of cells had new CENP-A localization (i.e. HeLa-Int-03 13hr and KAT7KO 15hr) were selected, and used for image quantitation. Total nuclear Halo-CENP-A intensity and centromere-localized Halo-CENP-A intensities were quantitated. The average Halo-CENP-A intensity at each centromere was normalized to the total nuclear Halo-CENP-A intensity of each cell. Data are presented as mean + s.e. (n≥10 cells, >20 centromeres were analyzed in each cell). **P<0.005 (t-test).

Figure S5. Supplemental information for Figure 5, Related to Figure 5

(A) Suv39h1 immunoblotting. Total cell lysates of control and Suv39h1 depleted cells were analyzed. See also Figure 5B. (B) ChIP analysis. ChIP was carried out with anti-H3K9me3 antibody or beads alone (no antibody) as a control. Data are presented as mean + s.e. ($n \geq 3$). *$P<0.05$, **$P<0.005$ (t-test).

Figure S6. Supplemental information for Figure 6, Related to Figure 6

(A) Confirmation of H3K9me3 reduction by ChIP. ChIP was carried out with anti-GFP antibody, anti-H3K9me3 antibody or beads alone (no antibody) as a control. Data are presented as mean + s.d. ($n=3$). **$P<0.005$ (t-test). (B) CENP-A intensity quantitation of siRNA transfected cells in Figure 6E. Control (HeLa-Int-03 expressing tetR-EYFP) and target cells were co-seeded on coverslips, and arrested at G1/S with thymidine. The cells were fixed, stained with CENP-A and CENP-B antibody and then used for microscopy. The CENP-A signal at centromeres including CENP-B positive micronuclei was quantitated and plotted in the right panel. Data are presented as mean + s.e. ($n \geq 5$ micronuclei). *$P<0.05$, **$P<0.005$ (t-test). See also Figure 6E.

Figure S7. A models and Supplemental information about KAT7 functions, Related to Figure 7

(A) Models of KAT7 function. KAT7 acetylates not only histone H3 but also H4 (Miotto et al., 2010). CENP-C interacts with HJURP, and may have an additional essential role not shared by the Mis18-KAT7 complex in the *de novo* endogenous CENP-A assembly pathway (Zasadzińska et al., 2013; Müller et al., 2014; Tachiwana et al., 2015; Shono et al., 2015). See also the Discussion. (B) Quantitation of alphoid^{tetO} RNA levels. (Top) Schema of time course. TetR-EYFP-fusion protein-expressing cells were synchronized

at G1/S with thymidine. Cells were then released and cultured for 12 hour (at this time point, most of cells were in G1 phase) in the presence of doxycycline. Doxycycline was washed out 2 hour before RNA harvesting. The amount of RNA was quantified by quantitative RT-PCR. (Bottom) Alphoid^{tetO} RNA levels were normalized with *HPRT* and plotted in the graph. Data are presented as mean + s.d. (n=3). *P<0.05 (t-test). (C) ChIP analysis combined with transient transfection using a non-alphoid DNA sequence. (Top) Plasmids used for this study. Plasmid pWTO2R contains 60kb of alphoid^{tetO} repeat DNA insert (Ohzeki et al., 2012). Plasmid pRF322TO2R contains a synthetic repeat of pBR322 restriction fragment (Ohzeki et al., 2002) combined with the tetO site. These plasmids contain a Neomycin resistant gene cassette. Plasmid pWTO2R or pRF322TO2R, was respectively transfected into HeLa cells and selected with Geneticin (selective drug for Neomycin) for 3 weeks. Next, vectors expressing tetR-EYFP-fusion proteins and Halo-CENP-A were transfected to the cells. After 12 hours of tethering, cells were harvested for ChIP. (Bottom) ChIP analysis. ChIP was carried out with anti-CENP-A antibody, anti-GFP antibody or beads alone (no antibody) as a control. Data are presented as mean + s.d. (n=3). **P<0.005 (t-test). (D) KAT7 overproduction bypasses the absence of hMis18α. (Top) Scheme for hMis18α depletion and KAT7 overproduction. (Middle) Examples of staining. Fluorescence images were obtained by DAPI staining (DNA), EYFP (green), and Halo-TMR-ligand (red). Scale bars = 5 μm. (Bottom) Data are presented as mean + s.d. (n=3, >200 cells were counted in each sample). *P<0.05 (t-test).

Table S1. siRNAs Used in this Study, Related to Experimental Procedure

Target gene	Sequence	Reference
Control (siNegative)	No information	Ambion, AM4611
hMis18α	5'-CAGAAGCUAUCCAAACGUGTT-3'	Fujita et al., 2007
hMis18β	5'-CAAUCGCUUAAAAUCACUAtt-3'	Ambion, s22367
M18BP1	5'-GAAGUCUGGUGUUAGGAAATT-3'	Fujita et al., 2007
HJURP	5'-CUACUGGGCUCAACUGCAAUU-3'	Dunleavy et al., 2009
KAT7	5'-GGAUGCCCACUGUAUCAUAtt-3'	Ambion, s253
BRPF2	5'-GAAACUAUAGACAAGUUAAtt-3'	Ambion, s24388
ING4	5'-UGCUCGUGCUCGUUCCAAAtt-3'	Ambion, s27553
ING5	5'-CCUUACCACGAAACCCAAAtt-3'	Ambion, s38829
RSF1	5'-GGAUAUCAAGUCGGAAAAAtt-3'	Ambion, s28648
Suv39h1	5'-AGAACAGCUUCGUCAUGGAtt-3'	Ambion, s13658

Table S2. Plasmid Vectors Used in this Study, Related to Experimental Procedure

Name	Backbone	Cloned genes	Reference
pJETY3		tetR-EYFP-alone	Ohzeki et al., 2012
pJETY3-hMis18α	pJETY3	tetR-EYFP-hMis18α	Ohzeki et al., 2012
pJETY3-hMis18β	pJETY3	tetR-EYFP-hMis18β	This study
pJETY3-M18BP1-I	pJETY3	tetR-EYFP-M18BP1-I	This study
pJETY3-M18BP1-II	pJETY3	tetR-EYFP-M18BP1-II	This study
pJETY3-M18BP1-III	pJETY3	tetR-EYFP-M18BP1-III	This study
pJETY3-M18BP1-IV	pJETY3	tetR-EYFP-M18BP1-IV	This study
pJETY3-KAT7	pJETY3	tetR-EYFP-KAT7	This study
pJETY3-KAT7 G485A	pJETY3	tetR-EYFP-KAT7 G485A	This study
pJETY3-PCAFHD	pJETY3	tetR-EYFP-PCAFHD	Ohzeki et al., 2012
pJETY3-BRPF2	pJETY3	tetR-EYFP-BRPF2	This study
pJETY3-ING4	pJETY3	tetR-EYFP-ING4	This study
pJETY3-ING5	pJETY3	tetR-EYFP-ING5	This study
pJETY3-RSF1	pJETY3	tetR-EYFP-RSF1	This study
pJETY3-JMJD2B	pJETY3	tetR-EYFP-JMJD2B	This study
pJETB3		tetR-BFP-alone	This study
pJETB3-hMis18α	pJETB3	tetR-BFP-hMis18α	This study
pJETB3-hMis18β	pJETB3	tetR-BFP-hMis18β	This study
pJ3-EYFP		EYFP	This study
pJ3-EYFP-KAT7	pJ3-EYFP	EYFP-KAT7	This study
pJ3-EYFP-BRPF2	pJ3-EYFP	EYFP-BRPF2	This study
pJ3-EYFP-ING4	pJ3-EYFP	EYFP-ING4	This study
pJ3-EYFP-ING5	pJ3-EYFP	EYFP-ING5	This study
pJ4IB-3HA-M18BP1-I	pJ4IB-3HA	3xHA-M18BP1-I	This study
pJ4IB-3HA-M18BP1-IV	pJ4IB-3HA	3xHA-M18BP1-IV	This study
pJ4IB-3HA-hMis18β	pJ4IB-3HA	3xHA-hMis18β	This study
pJ4IB-3HA-ING4	pJ4IB-3HA	3xHA-ING4	This study
pJ4IB-3FL-KAT7	pJ4IB-3FL	3xFLAG-KAT7	This study
pJ4IB-3FL-hMis18α	pJ4IB-3FL	3xFLAG-hMis18α	This study
pJ4IB-3FL-ING5	pJ4IB-3FL	3xFLAG-ING5	This study
pJ4-Halo7		Halo-tag	
pJ4IB-Halo7-Suv39h1	pJ4-Halo7	Halo-Suv39h1	This study
pJ4IB-Halo7-M18BP1-I	pJ4-Halo7	Halo-M18BP1-I	This study
pJ4IB-Halo7-M18BP1-II	pJ4-Halo7	Halo-M18BP1-II	This study
pJ4IB-Halo7-M18BP1-III	pJ4-Halo7	Halo-M18BP1-III	This study
pJ4IB-Halo7-M18BP1-IV	pJ4-Halo7	Halo-M18BP1-IV	This study
pJTI PhiC31 Int		PhiC31 integrase	Life Technologies, A10894
pX260		Cas9, gRNA	Cong et al., 2013
pJ4IB-Cas9		Cas9	This study
pU6CR-KAT7		gRNA for KAT7 KO	This study
pWTO2R	pBAC108L	tetO alphoid DNA	Ohzeki et al., 2012
pRF322TO2R	pBAC108L	synthetic repeat DNA	This study

Name	Backbone	Cloned genes	Reference
pFN21A		Halo-tag	Promega, G2821
pFN21A-Halo-NLS	pFN21A	Halo-NLS	This study
pFN21A-Halo-hMis18α	pFN21A	Halo-hMis18α	FHC27152
pFN21A-Halo-hMis18β	pFN21A	Halo-hMis18β	FHC25353
pFN21A-Halo-M18BP1	pFN21A	Halo-M18BP1	FHC11044
pFN21A-Halo-KAT1	pFN21A	Halo-KAT1	FHC21224
pFN21A-Halo-KAT2A	pFN21A	Halo-KAT2A	FHC03762
pFN21A-Halo-KAT2B	pFN21A	Halo-KAT2B	FHC11610
pFN21A-Halo-KAT3A	pFN21A	Halo-KAT3A	FHC11778
pFN21A-Halo-KAT3B	pFN21A	Halo-KAT3B	FHC01787
pFN21A-Halo-KAT5	pFN21A	Halo-KAT5	FHC02643
pFN21A-Halo-KAT6A	pFN21A	Halo-KAT6A	FHC03163
pFN21A-Halo-KAT6B	pFN21A	Halo-KAT6B	FHC00523
pFN21A-Halo-KAT7	pFN21A	Halo-KAT7	FHC06090
pFN21A-Halo-KAT8	pFN21A	Halo-KAT8	FHC02573
pFN21A-Halo-KAT9	pFN21A	Halo-KAT9	FHC02650
pFN21A-Halo-KAT12	pFN21A	Halo-KAT12	FHC11853
pFN21A-Halo-KAT13B	pFN21A	Halo-KAT13B	FHC01529
pFN21A-Halo-KAT13C	pFN21A	Halo-KAT13C	FHC01199
pFN21A-Halo-KAT13D	pFN21A	Halo-KAT13D	FHC00509
pFN21A-Halo-Suv39h1	pFN21A	Halo-Suv39h1	FHC09879
pFN21A-Halo-BRPF1	pFN21A	Halo-BRPF1	FHC01228
pFN21A-Halo-BRPF2	pFN21A	Halo-BRPF2	FHC02120
pFN21A-Halo-BRPF3	pFN21A	Halo-BRPF3	FHC01683
pFN21A-Halo-EPC1	pFN21A	Halo-EPC1	FHC11123
pFN21A-Halo-EPC2	pFN21A	Halo-EPC2	FHC30908
pFN21A-Halo-JADE1	pFN21A	Halo-JADE1	FHC09414
pFN21A-Halo-JADE2	pFN21A	Halo-JADE2	FHC00474
pFN21A-Halo-JADE3	pFN21A	Halo-JADE3	FHC00463
pFN21A-Halo-ING3	pFN21A	Halo-ING3	FHC02414
pFN21A-Halo-ING4	pFN21A	Halo-ING4	FHC02495
pFN21A-Halo-ING5	pFN21A	Halo-ING5	FHC03674
pFN21A-Halo-CENP-28	pFN21A	Halo-CENP-28	FHC04343
pFN21A-Halo-RSF1	pFN21A	Halo-RSF1	FHC11761
pFN21A-Halo-HJURP	pFN21A	Halo-HJURP	FHC28790
pFN21A-Halo-CENP-A	pFN21A	Halo-CENP-A	FHC20935
pFN21A-Halo-hisone H3.3	pFN21A	Halo-hisone H3.3	This study
pFN21A-Halo-CDT1	pFN21A	Halo-CDT1	This study
pFN21A-Halo-CDC6	pFN21A	Halo-CDC6	FHC10183
pFN21A-Halo-Geminin	pFN21A	Halo-Geminin	FHC10644
pFN21A-Halo-MCM7	pFN21A	Halo-MCM7	FHC05950
pFN21A-Halo-RPA1	pFN21A	Halo-RPA1	FHC01462
pFN21A-Halo-ATR	pFN21A	Halo-ATR	FHC11858
pFN21A-Halo-RB1	pFN21A	Halo-RB1	FHC01227
pFN21A-Halo-p53	pFN21A	Halo-p53	FHC10374
pFN21A-Halo-53BP1	pFN21A	Halo-53BP1	FHC00998
pFN21A-Halo-RAD51	pFN21A	Halo-RAD51	FHC10574
pFN21A-Halo-RAD52	pFN21A	Halo-RAD52	FHC10450
pFN21A-Halo-Lig4	pFN21A	Halo-Lig4	FHC10347

Note; Halo-tag fused genes are available at Kazusa DNA Research Institute Human cDNA/ORF Clone distribution (http://www.kazusa.or.jp/kop/dsearch-e/).

Table S3. Cell Lines Used in this Study, Related to Experimental Procedure

Name	Host cell	Transfected alphoid DNA	Event	Modified genes	Expressing genes	Reference
HeLa-Int-03	HeLa	pWTO2R (Chr. 21-based tetO-Alphoid DNA repeat)	Integration	None	None	Ohzeki et al., 2012
Int-03+KAT7	HeLa	pWTO2R	Integration	None	EYFP-KAT7	This study
Int-03+Suv39h1	HeLa	pWTO2R	Integration	None	Halo-Suv39h1	This study
Int-03+tR-alone	HeLa	pWTO2R	Integration	None	tetR-EYFP-alone	This study
KAT7KO	HeLa	pWTO2R	Integration	KAT7 knockout	None	This study
KAT7KO+KAT7	HeLa	pWTO2R	Integration	KAT7 knockout	EYFP-KAT7	This study
KAT7KO+Suv39h1	HeLa	pWTO2R	Integration	KAT7 knockout	Halo-Suv39h1	This study

Table S4. Antibodies Used in this Study, Related to Experimental Procedure

Antibodies	Catalog number	Antibody produced in	Usage	Reference
anti-CENP-A (A1)	None	Mouse	Immunostain, Immunoblot, ChIP	Ohzeki et al., 2002
anti-CENP-A (6F2)	None	Rat	Immunostain	Kind gift from Kinya Yoda
anti-CENP-B (5E6C1)	None	Mouse	Immunostain	Ohzeki et al., 2002
anti-KAT7	abcam, ab70183	Rabbit	Immunostain, Immunoblot, IP	
anti-Suv39h1	SIGMA, S8316	Mouse	Immunoblot	
anti-M18BP1	NOVUS, NBP1-47290	Rabbit	Immunoblot, IP	
anti-HJURP (27D1)	None	Mouse	Immunoblot	This study
anti-RSF1 (EPR3749)	GeneTex, GTX62703	Rabbit	Immunoblot	
anti-H3K14ac (7G8)	None	Mouse	Immunostain, Immunoblot, ChIP	This study
anti-H3K9me3	WAKO, MABI 0308	Mouse	ChIP	
anti-H3K9me3	abcam, ab8898	Rabbit	Immunostain	
anti-Cyclin B	SIGMA, C-8831	Rabbit	Immunostain	
anti-GFP	MBL, 598	Rabbit	Immunoblot	
anti-Halo-tag	Promega, G928A	Rabbit	Immunoblot	
anti-HA-7	SIGMA, H3663	Mouse	Immunoblot	
anti-FLAGM2	SIGMA, F3165	Mouse	Immunoblot	
anti-tubulin	abcam, ab6160	Rat	Immunostain	

Table S5. Primer DNA Sequences Used for Quantitative PCR, Related to Experimental Procedure

Name	primer 1 (5' to 3')	primer 2 (5' to 3')	Reference
5S Ribosomal DNA	ACGCTGGGTTCCCTGCCGTT	TGGCTGGCGTCTGTGGCACCCGCT	Ohzeki et al., 2012
Satellite 2	TCGCATAGAATCGAATGGAA	GCA TTCGAGTCCGTGGA	Ohzeki et al., 2012
X alphoid	AGA TTTGGACCGCTTTGAGGC	CCGTTCAGTTATGGAAGTTGA	Ohzeki et al., 2012
21-I alphoid	CTAGACAGAAGCCCTCTCAG	GGGAAGACA TTCCCTTTTTCACC	Ohzeki et al., 2012
tetO alphoid	GTGGAATCTGCAAGTGGATATTTGAC	CTGATAGGGAGAGCTCTGCTGCTAG	Ohzeki et al., 2012
RF322T(synthetic repeat)	TGGCCGGCATCACCGGCGCCAGAGGTC	TGATATCGCCCAACAGTCCCCCGGCCA	This study
HPRT mRNA	GGACCCCACGAAGTGTTGG	CTGGCGATGTCAATAGGACTCCAG	Ohzeki et al., 2012
CENP-A mRNA	AAAGCTTCAGAAGAGCACACACCTCTTG	CATGTAAGGTGAGGAGATAGGCGTCCTC	This study
hMis18α mRNA	CATCCTGCTTCGCTGTGTTTCCTG	TTGAGTGAGCACCCCGCGCAGCAC	Ohzeki et al., 2012
hMis18β mRNA	GGTGCACCTCGCCTGGGACCTGTCGCGG	AACTAGGAAGGGCGCTTCCAAAACGACG	This study
M18BP1 mRNA	ACTAAGAGCCTCAGTCCAAGGAGTTCCTC	GGCACTGAATCTTTTGTTTGACCGGGAGG	This study
HJURP mRNA	GCCATCATCACCCCTGGGGTGCAG	GCAACACATGTAGATGAAGGAGCCTC	Ohzeki et al., 2012
KAT7 mRNA	TCTCCGCTACCTGCATAATTTTCAAGGC	TTGGAGTTGGACCTTTTGGCCTCTTTGG	This study
BRPF2 mRNA	CAGTCTCAGCGAAGCTCACAGCAGAGAG	CCGCAGCTCCATGGCGACCTGCTCCACC	This study
ING4 mRNA	AACTTTCAGCTCATGAGGGACCTAGACC	TTTTCCTCGGAGCTCAGGCTGCGGGCAC	This study
ING5 mRNA	AACTTCCAGCTGATGCGAGAGCTGGACC	TGGAGACAGCGTCTTCACCGTGGAGATG	This study
RSF1 mRNA	CTGCAGACAGATGGGAAAAATATTTGATC	GACACTCACAGAGGTACTTTAAGAGTGC	This study

Supplemental references

Avvakumov, N., Lalonde, M.E., Saksouk, N., Paquet, E., Glass, K.C., Landry, A.J., Doyon, Y., Cayrou, C., Robitaille, G.A., Richard, D.E., et al. (2012). Conserved molecular interactions within the HBO1 acetyltransferase complexes regulate cell proliferation. Mol Cell Biol. *32*, 689-703.

Mishima, Y., Miyagi, S., Saraya, A., Negishi, M., Endoh, M., Endo, T.A., Toyoda, T., Shinga, J., Katsumoto, T., Chiba, T., et al. (2011). The Hbo1-Brd1/Brpf2 complex is responsible for global acetylation of H3K14 and required for fetal liver erythropoiesis. Blood. *118*, 2443-53.

Müller, S., Montes, de Oca, R,, Lacoste, N., Dingli, F., Loew, D., Almouzni, G. (2014). Phosphorylation and DNA binding of HJURP determine its centromeric recruitment and function in CenH3(CENP-A) loading. Cell Rep. *8*, 190-203.

Ohta, S., Bukowski-Wills, J.C., Sanchez-Pulido, L., Alves. Fde. L., Wood, L., Chen, Z.A., Platani, M., Fischer, L., Hudson, D.F., Ponting, C.P., et al. (2010). The protein composition of mitotic chromosomes determined using multiclassifier combinatorial proteomics. Cell. *142*, 810-21.

Tachiwana, H., Müller, S., Blümer, J., Klare, K., Musacchio, A., Almouzni, G. (2015). HJURP involvement in de novo CenH3(CENP-A) and CENP-C recruitment. Cell Rep. *11*, 22-32.

Zasadzińska, E., Barnhart-Dailey, M.C., Kuich, P.H., Foltz, D.R. (2013). Dimerization of the CENP-A assembly factor HJURP is required for centromeric nucleosome deposition. EMBO J. *32*, 2113-24.
